# Zirconium Carboxy-Aminophosphonate Nanosheets Loaded with Nickel-Boride Nanoparticles as an Efficient and Recyclable Catalyst for Nitroarene and Alkene Hydrogenation

**DOI:** 10.1021/acsomega.6c02980

**Published:** 2026-07-07

**Authors:** Martina Pancotto, Anna Donnadio, Beatrice Muzzi, Morena Nocchetti, Oriana Piermatti

**Affiliations:** † 9309 Università degli studi di Perugia, Dipartimento di Chimica Biologia e Biotecnologie, Via Elce di Sotto 8, Perugia 06123, Italy; ‡ Università degli studi di Perugia, Dipartimento di Scienze Farmaceutiche, Via del Liceo 1, Perugia 06123, Italy; § 201843 Istituto di Chimica dei Composti Organo Metallici—C.N.R, Sesto Firentino, Firenze 50019, Italy

## Abstract

Stabilized nickel-boride (Ni_*x*_B) nanoparticles immobilized on zirconium carboxy-aminophosphonate nanosheets (**Ni@ZrPGly**) were synthesized, fully characterized, and applied as efficient and recyclable catalysts for the selective catalytic hydrogenation of nitroarenes and alkenes under very mild reaction conditions. The nanocatalysts were obtained by ion exchange of the acidic protons of exfoliated ZrPGly with nickel ions, followed by reduction, resulting in a uniform dispersion of Ni_*x*_B nanoparticles on the lamellar surface. Among the samples prepared with varying nickel loadings, **Ni@ZrPGly-1**, which possessed the highest Ni content, showed the greatest activity and selectivity in the reduction of nitroarenes using NaBH_4_ as a hydrogen source in methanol at 30 °C. The catalyst exhibited excellent recyclability, maintaining its catalytic performance over at least five consecutive cycles with negligible Ni leaching. A comparison with the completely inorganic zirconium phosphate nanocatalyst, **Ni@ZrP**, highlights the importance of the glyphosine groups in the formation and stabilization of Ni-boride nanoparticles. Moreover, **Ni@ZrPGly-1** efficiently catalyzed the hydrogenation of alkenes under similar conditions, demonstrating its versatility as a sustainable heterogeneous catalyst.

## Graphical abstract

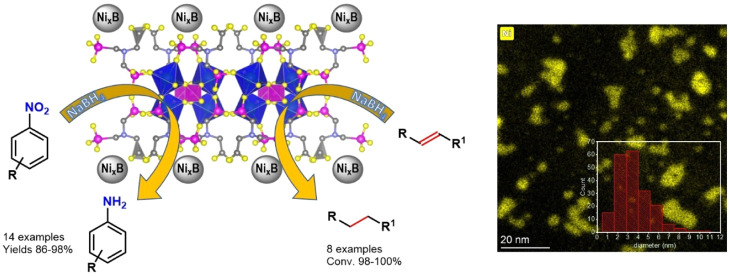

## 1 Introduction

The development of sustainable and environmentally benign chemical processes has become a central challenge in modern synthetic chemistry.^1–3^ Among the various transformations, hydrogenation of nitroarenes and alkenes plays a pivotal role in the fine and bulk chemical industries.^4–7^ The conversion of nitroarenes into anilines represents a key transformation from both environmental and synthetic perspectives, as nitro compounds are considered harmful pollutants, while functionalized anilines are important building blocks for the synthesis of pharmaceuticals, agrochemicals, dyes, polymers, surfactants, and advanced materials.^4,5,8–11^ Their wide-ranging applications underscore the importance of developing selective and efficient, environmentally sustainable, and scalable methodologies for their production. Similarly, olefin hydrogenation represents a fundamental transformation in both academic and industrial research, owing to its widespread application in the synthesis of fine chemicals, active pharmaceutical ingredients, agrochemicals, food additives, and fragrances.^6,7,12–14^

Traditional hydrogenation methods based on stoichiometric reducing agents (e.g., Fe, Zn, Sn), high temperatures, and/or high H_2_ pressure are unsustainable because of the significant waste generation, whereas catalytic hydrogenation that uses active hydrogen species produced in situ provides a greener alternative by improving atom economy and reducing toxic byproducts. Various hydrogen donors such as silane, ammonia borane, hydrazine hydrate, and sodium borohydride, among others, have been employed for the transfer hydrogenation reactions as alternatives to the traditional high-pressure hydrogenation process.^5,15–17^

Noble-metal catalysts such as Pt, Pd, Au, and Ru have demonstrated excellent activity and selectivity in hydrogenation reactions. However, their high cost, scarcity, and environmental footprint limit their widespread application.^18^ This has driven increasing interest in earth-abundant transition metals, particularly nickel, as a cost-effective and sustainable substitute.^19^ Nickel nanoparticles (Ni NPs) display remarkable catalytic performance due to their high surface-to-volume ratio, tunable morphology, and strong hydrogenation ability, making them competitive with precious metals while offering substantial economic and ecological benefits.^20–23^

Nevertheless, one of the main challenges with Ni NPs lies in their tendency to agglomerate due to high surface energy, which diminishes their catalytic performance. To overcome this limitation, the design of supported Ni-based catalysts has proven essential. The choice of support not only stabilizes nanoparticles against aggregation and sintering but also modulates metal–support interactions, thereby enhancing activity, selectivity, and recyclability.^20,21,24,25^

In this context, layered zirconium phosphates and phosphonates have emerged as promising supports for metal nanoparticles owing to their high surface accessibility, excellent chemical and thermal stability, and easily tunable structural features.^26–28^ Their layered framework allows the incorporation of various organic groups (e.g., polar, hydrophobic, or chelating groups), which enhance the efficiency of metal nanoparticle immobilization and stabilization. These materials can be readily prepared through a self-assembly approach, enabling fine control over their chemical and physical properties through a careful selection of the building blocks.^26^

Among them, zirconium phosphate bis­(phosphonomethyl)­glycine (ZrPGly), with the formula unit Zr_2_(PO_4_)­H_5_[(O_3_PCH_2_)_2_NCH_2_COO]_2_·H_2_O, obtained using glyphosine and phosphate as building blocks, has attracted considerable interest due to its ability to undergo exfoliation under mild conditions, thereby maximizing the number of easily accessible surface ionogenic groups.^29^ The exfoliated ZrPGly allows efficient interactions with metal cations and metal nanoparticles through its exposed phosphonate and carboxylic groups. When a dispersion of exfoliated ZrPGly was impregnated with a Pd­(OAc)_2_ solution for 15 days, the in situ formation of uniformly distributed Pd nanoparticles with an average size of approximately 2 nm on the nanosheet surface was achieved (Pd@ZrPGly).^30^ The resulting nanocatalyst was initially employed in the sustainable Suzuki–Miyaura coupling reaction between aryl bromides and phenylboronic acid under both batch and flow conditions using 96% ethanol as a green solvent and K_2_CO_3_ as the base.^30,31^ The reactions afforded the corresponding biaryls in excellent yields (96–98%) with negligible Pd leaching (3–5 ppm).^30^ The nanocatalyst also exhibited outstanding catalytic activity in the Heck coupling reaction, performed in a CH_3_CN/H_2_O azeotrope with triethylamine as the base at 120 °C.^31^ High yields (74–98%) were obtained across a wide range of substrates, and only 2 ppm Pd leaching was detected under flow conditions. Notably, in both Suzuki–Miyaura and Heck reactions, the catalyst preserved its activity over several consecutive reaction cycles. The high activity and the very low Pd leaching were attributed to the strong stabilization of Pd nanoparticles through interactions with the phosphate–carboxyaminophosphonate groups on the nanosheet surface.^30,31^

A limited number of studies have reported the use of ZrP-supported metal nanoparticles, such as palladium, gold, platinum, and silver, for the selective reduction of nitroarenes.^26^ In a study from our research group, Au nanoparticles were immobilized on zirconium phosphate aminoethylphosphonate, a layered material functionalized with aminoethyl groups on the surface. The resulting Au-based nanocatalyst showed excellent activity in the selective reduction of nitroarenes, allowing the selective formation of either azoxyarenes or anilines by simply changing the reaction medium (96% ethanol or absolute ethanol, respectively) in the presence of NaBH_4_ as the reducing agent. Moreover, the transfer of the process from batch to flow conditions improved catalyst efficiency and stability, while maintaining very low Au leaching, owing to the stable packing of the catalyst inside the reactor and enabling the development of a simpler and greener protocol.^32^

Moreover, Pd@ZrPGly proved to be highly effective in the partial hydrogenation of alkynes, showing excellent Z-alkene selectivity and complete conversion under 1 bar of H_2_ at room temperature in methanol. The catalyst also showed good performance in the hydrogenation of nitroarenes to anilines under an H_2_ pressure of 5 bar, achieving full chemoselectivity for 3-fluoronitrobenzene, high stability over three consecutive cycles, and negligible metal leaching. For the 3-chloro- and 4-formyl-substituted substrates, the chemoselectivity was slightly lower (84%), whereas complete dehalogenation was observed for 4-bromonitrobenzene.^33^

In light of these considerations, supported nickel-based nanoparticles immobilized on ZrPGly stand out as a promising class of catalysts for the sustainable hydrogenation of nitroarenes and alkenes, combining the advantages of earth-abundant metals with the enhanced stability and efficiency provided by advanced support systems.

In this paper, we report the synthesis and characterization of ZrPGly nanosheets loaded with nickel ions, **Ni@ZrPGly**, for the in situ preparation of immobilized amorphous nickel-boride species (Ni_*x*_B)^34–38^ and the subsequent hydrogenation of nitroarenes and alkenes using sodium borohydride as a cheap, easy-handling, and readily available hydrogen source.

## 2 Results and Discussion

### 2.1 Catalyst Preparation and Characterization

For the reader’s convenience, Figure 1 reports the synthetic procedure used to prepare **Ni@ZrPGly**, along with the ZrPGly structure. The layered ZrPGly was prepared by mixing aqueous solutions of zirconium fluorocomplexes with phosphoric acid and glyphosine as building blocks, as described previously.^29^ Exfoliation of ZrPGly with propylamine was performed to promote the separation of the layers, thereby increasing the exposed surface area and the accessibility of the ionogenic groups.^39,40^ To immobilize nickel ions on the layers, the colloidal dispersion was first treated with HCl to remove the amine while maintaining a high degree of dispersion of the ZrPGly nanosheets with exchangeable protons.^39,40^ Finally, the regenerated colloidal dispersion was equilibrated with increasing amounts of nickel acetate relative to the ion-exchange capacity (IEC) of ZrPGly. The recovered samples appeared green, confirming the successful immobilization of nickel. Table 1 reports the nominal and experimental compositions of the **Ni@ZrPGly** samples and **Ni@ZrP**, prepared for comparison.

**Figure 1:**
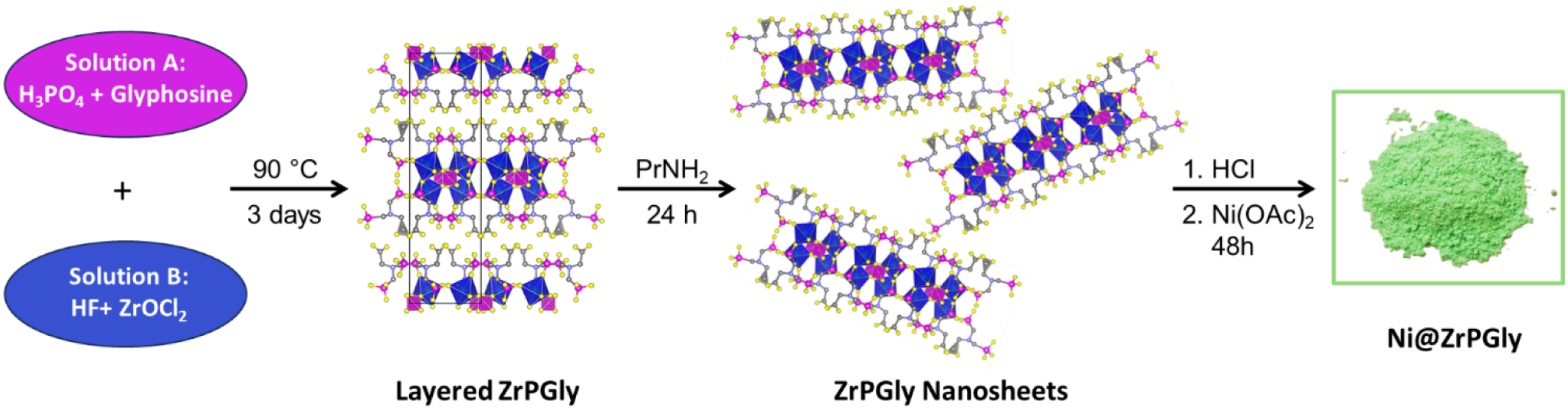
Schematic representation of the synthetic procedure for **Ni@ZrPGly** preparation, alongside ZrPGly structure.

**Table 1:** Nickel Content of the Catalysts is Expressed Both as Nominal (Ni^2+^ Added) and Experimental (Ni^2+^ Exp) Mol Percentages Relative to IEC, as Well as in Terms of Weight Percentage

sample	Ni^2+^ added (% IEC)	Ni^2+^ exp (% IEC)	Ni^2+^ exp (%w/w)
**Ni@ZrPGly-1**	200	100	12.2
**Ni@ZrPGly-2**	100	73.7	8.2
**Ni@ZrPGly-3**	50	41.6	4.6
**Ni@ZrP**	50	38.9	7.6

Figure 2A shows the XRD patterns of the nickel-supported samples compared with the non-exfoliated pristine material. All the exchanged samples display diffraction patterns indicative of partial restacking of the layers upon nickel incorporation. In the fully exchanged material, **Ni@ZrPGly-1**, the interlayer distance increases from 14.8 Å (in ZrPGly) to 18.4 Å, confirming the presence of Ni ions between the reaggregated layers. This interlayer distance depends on the nickel content: it decreases to 16.5 Å and 16 Å in samples with lower Ni loadings, such as **Ni@ZrPGly-2** and **Ni@ZrPGly-3**, respectively. The presence of the reflection associated with the (002) atomic planes, which depends on the internal layer structure, indicates that the layer structure is preserved, as expected for an ion-exchange process. The restacking and intercalation of Ni^2+^ in **Ni@ZrP** are also demonstrated by the increase in interlayer distance from 7.5 Å (in α-ZrP) to 10.3 Å (Figure S1). Insights into the interaction of Ni^2+^ ions with ZrPGly were obtained from the ATR-FTIR spectra of **Ni@ZrPGly** samples reported in Figure 2B. The pristine ZrPGly shows, in the 500–2000 cm^–1^ region, a band at 1732 cm^–1^ assigned to the C=O stretching vibration of −COOH groups, together with bands at 1227 cm^–1^ (C–O stretching) and 1420 cm^–1^ (O–H bending). The bands in the 950–1200 cm^–1^ region are related to P–O stretching vibrations. In particular, the main contribution arises from PO_4_^–3^ groups, which exhibit a ν_3_ asymmetric stretching band centered at 1011 cm^–1^. The shoulders at 1065 and 965 cm^–1^, along with the band at 850 cm^–1^, can be attributed to ν_3_ and ν_1_ modes of −PO_3_H^–^ groups of glyphosine. Below 900 cm^–1^, several bands are observed that are not straightforward to assign, as they likely arise from a combination of bending modes and Zr–O stretching vibrations, as previously reported.^41^

**Figure 2:**
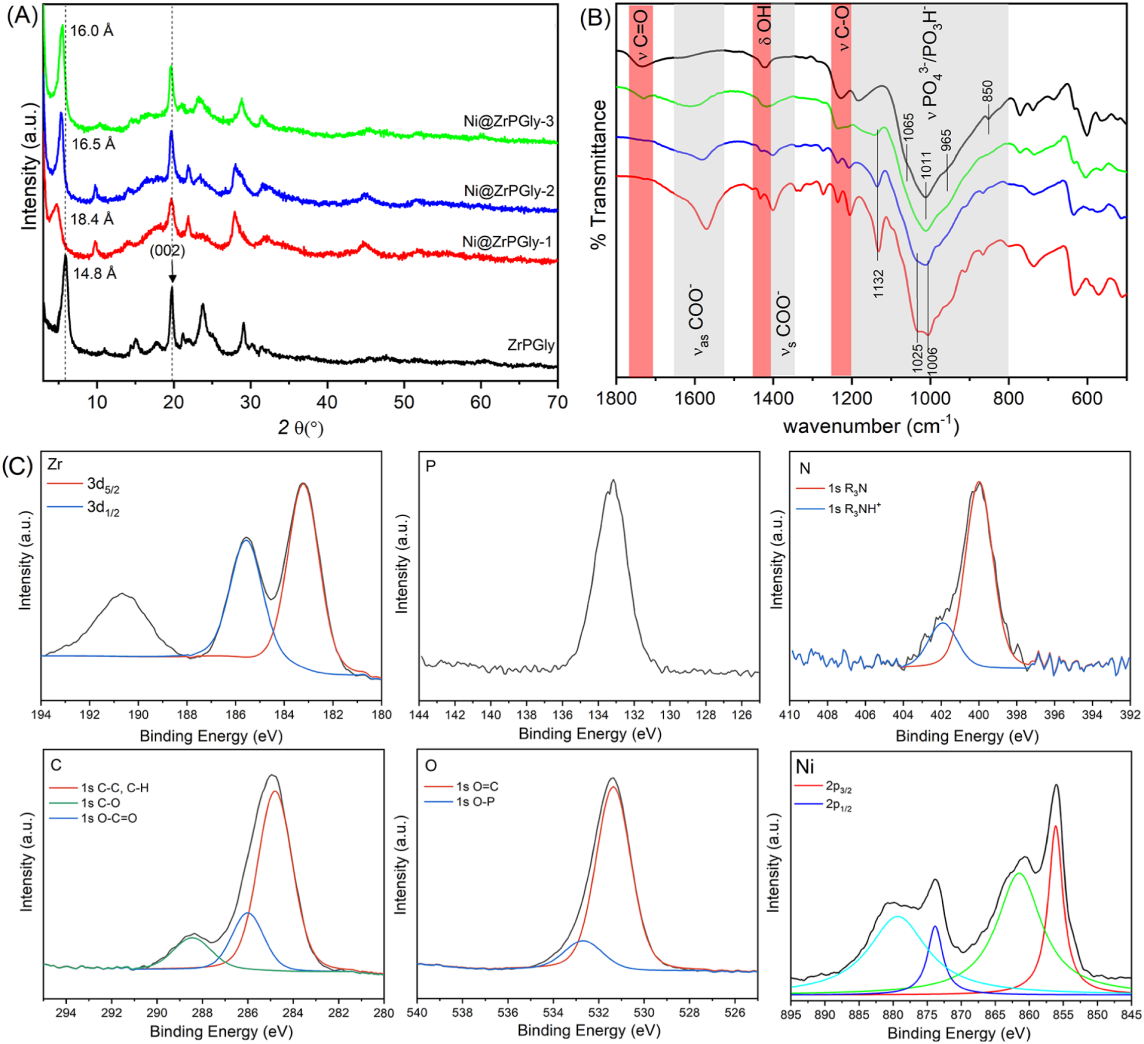
XRD patterns (A) and ATR-FTIR (B) of ZrPGly (black), **Ni@ZrPGly-1** (red), **Ni@ZrPGly-2** (blue), and **Ni@ZrPGly-3** (green). XPS of **Ni@ZrPGly-1** (C).

Upon Ni^2+^ intercalation, significant modifications of the ATR-FTIR spectra are observed. In the fully exchanged sample (**Ni@ZrPGly-1**), the absorption bands associated with −COOH groups disappear and are replaced by those of carboxylate groups (−COO^–^), with asymmetric and symmetric stretching vibrations at 1571 and 1400 cm^–1^, respectively. Furthermore, the phosphate region becomes more structured, with the appearance of bands at 1132, 1025, and 1006 cm^–1^, along with additional features below 1000 cm^–1^. These changes are consistent with a reduction in symmetry of the phosphonate groups due to coordination with Ni^2+^ ions.^42^

Overall, these findings indicate that Ni^2+^ interacts with both the carboxylic and phosphonate groups of the support. Samples with lower Ni^2+^ content show the coexistence of −COOH and −COO^–^ absorptions, with the intensity of the latter increasing as the nickel loading increases. This trend is consistent with the XRD results.

The interaction of nickel ions with the support was also investigated by XPS spectroscopy on **Ni@ZrPGly-1** (Figure 2C). In the Ni 2p XPS spectrum, the Ni 2p_3/2_ and Ni 2p_1/2_ peaks are located at 856.07 and 873.84 eV, respectively, together with the corresponding satellite peaks at 861.47 and 879.29 eV. The binding energies of the Ni 2p_3/2_ and Ni 2p_1/2_ signals are close to those reported for nickel phosphate, suggesting that the Ni^2+^ cations strongly interact with the P–O groups.^43^ The Zr 3d_5/2_ and 3d_3/2_ peaks, located at 183.20 and 185.55 eV, respectively, exhibit binding energies comparable to those reported for α-zirconium phosphonates.^44^ The P 2p_3/2_ peak at 133.27 eV is characteristic of phosphorus atoms belonging to diphosphonate groups. The N 1s XPS spectrum reveals the presence of two different chemical species: the peak at 400.00 eV is assigned to deprotonated amine groups (R_3_N), whereas the peak at 401.91 eV is attributed to protonated amine groups (R_3_NH^+^).^45^ The FTIR spectra of pristine ZrPGly showed that the amino groups are predominantly protonated.^29^ After ion exchange with nickel ions, the amount of protonated amine groups decreases significantly, suggesting coordination between Ni^2+^ and nitrogen atoms. The C 1s core-level spectrum can be deconvoluted into three components: a peak at 284.80 eV assigned to C–C and C–H bonds, a peak at 286.0 eV attributed to C–N, C–P, and C–COO bonds, and a peak at 288.44 eV associated with COO groups.^45^ In the O 1s core-level spectrum, two oxygen contributions were identified according to literature values. The first peak, centered at 531.35 eV, is attributed to carbonyl oxygen atoms (O=C), whereas the second peak, located at 532.89 eV, is assigned to O–P groups.^46^

Information on the morphology of the samples and on the degree of dispersion of Ni^2+^ was obtained by SEM–EDX analysis. Figure 3 shows that the samples are composed of nanometric particles, with diameters of approximately 100–200 nm, which tend to form very compact aggregates, making it difficult to distinguish individual particles. EDX analysis also reveals a homogeneous distribution not only of zirconium and phosphorus, which constitute the support, but also of the nickel immobilized on the surface of the lamellae.

**Figure 3:**
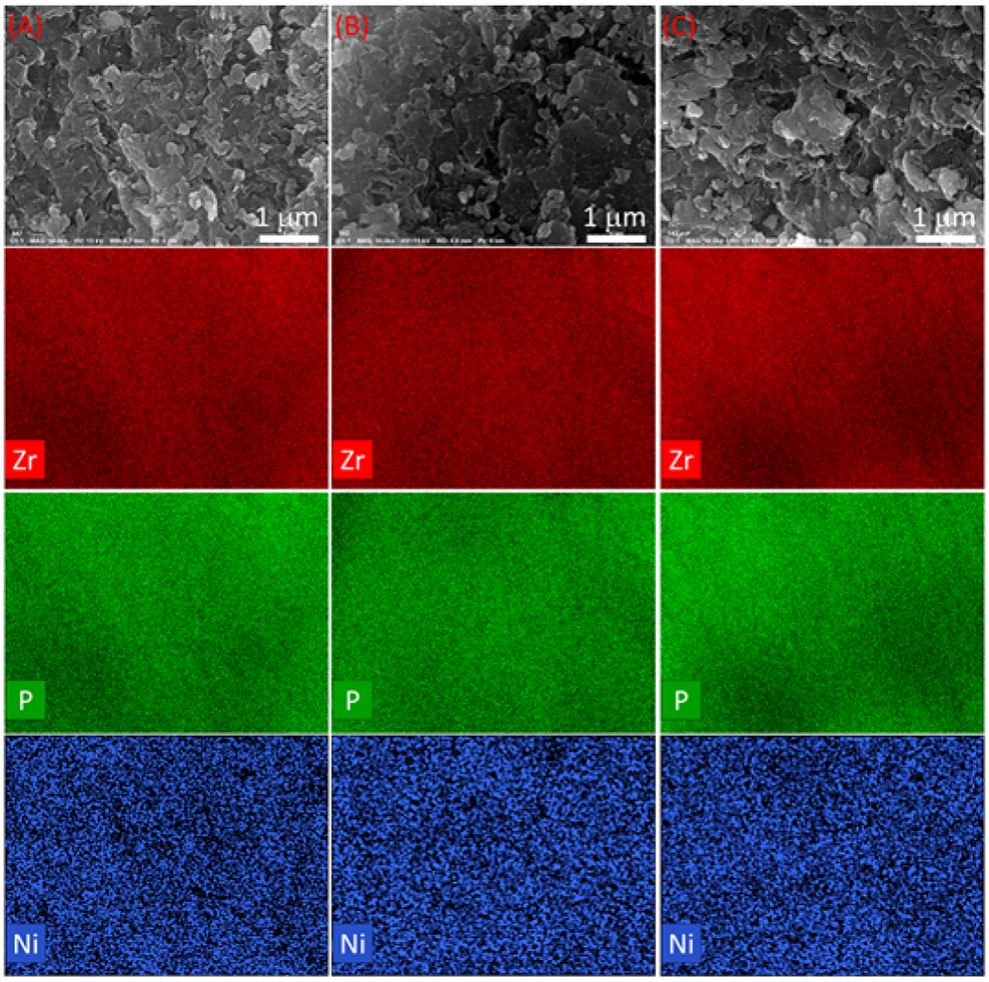
SEM and EDX analysis showing the distribution of Zr, P, and Ni of: **Ni@ZrPGly-1** (A), **Ni@ZrPGly-2** (B), and **Ni@ZrPGly-3** (C). The scale bar in the SEM micrographs applies to all corresponding EDX mapping panels.

### 2.2 Catalytic Activity

To determine the optimal reaction conditions, we began our study by evaluating the catalytic activity of **Ni@ZrPGly-1** in the reduction of 4-nitroanisole (**1a**). The reactions were carried out using N_2_H_4_ and NaBH_4_ as reducing agents and protic solvents such as EtOH, H_2_O, and MeOH for the *insitu* reduction of nickel nanocatalyst and the subsequent hydrogenation of the substrate (Table 2).

**Table 2:**
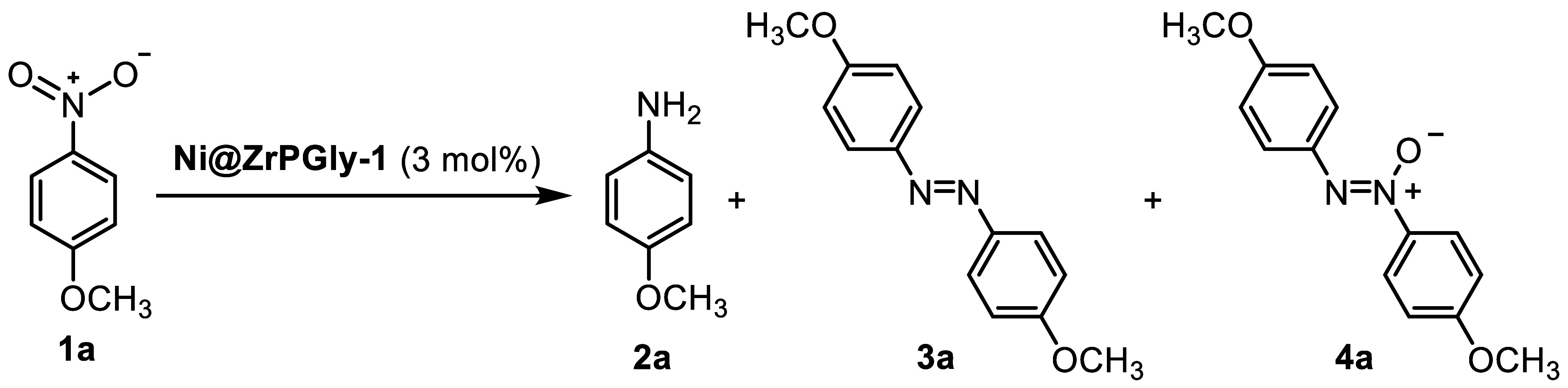
Optimization of Reaction Parameters for the Catalytic Hydrogenation of 4-Nitroanisole (**1a**) Using **Ni@ZrPGly-1**

Entry^a^	Reducing agent (eq )	Medium	T °C	t (h)	Run	Conv %^b^	**2a:3a:4a** ^b^
1	N_2_H_4_ (4 equiv)	EtOH 96%	60	20	1	0	-
2	N_2_H_4_ (4 equiv)	H_2_O	60	20	1	0	-
3	N_2_H_4_ (4 equiv)	H_2_O	80	20	1	0	-
4	NaBH_4_ (3 equiv)	EtOH 96%	30	3	1	8	37:0:63
5	NaBH_4_ (3 equiv)	EtOH abs	30	3	1	27	44:4:52
6	NaBH_4_ (3 equiv)	H_2_O	30	3	1	100	100:0:0
					2	100	100:0:0
					3	71	100:0:0
					4	51	100:0:0
7	NaBH_4_ (3 equiv)	H_2_O/EtOH (9:1)	30	3	1	100	100:0:0
					2	100	100:0:0
					3	49	100:0:0
8	NaBH_4_ (3 equiv)	H_2_O/EtOH (1:1)	30	3	1	100	100:0:0
					2	16	100:0:0
9	NaBH_4_ (3 equiv)	MeOH	30	3	1	100	100:0:0
					2	100	100:0:0
					3	100	100:0:0
					4	100	92:1:7
					5	100	80:2:18
10	NaBH_4_ (2 equiv)	MeOH	30	3	1	75	79:2:19
11^c^	NaBH_4_ (3 equiv)	MeOH	30	3	1	96	83:4:13

a Reaction conditions: **1a** (0.2 mmol), **Ni@ZrPGly-1** (3 mol %), N_2_H_2_ (4 equiv) or NaBH_4_ (3 equiv), medium (2 mL).

b Conversion and product distribution were determined by GLC analysis.

c 1 mol % of catalyst was used.

The optimization studies were carried out using 0.2 mmol of **1a** and 3 mol % of **Ni@ZrPGly-1** in a steel vial (2.5 mL) equipped with a magnetic stir bar, sealed with a steel screw cap, and filled with 2 mL of reaction medium. In addition, the recovery and recyclability of **Ni@ZrPGly-1** were also investigated under selected reaction conditions. After each catalytic run, the catalyst was isolated by centrifugation, washed with the corresponding reaction solvent to remove any residual product, and subsequently reused in the next cycle.

When N_2_H_4_ (4 equiv) was used as the hydrogen donor in either 96% EtOH or H_2_O at 60 or 80 °C for 20 h, no reduction products were observed (Table 2, entries 1–3). If NaBH_4_ (3 equiv) was used as the hydrogen donor in 96% EtOH or absolute EtOH at 30 °C for 3 h, a mixture of reaction products was obtained, with very low conversions of 8% and 27%, respectively (Table 2, entries 4 and 5).

Complete conversion and highly selective reduction of **1a** to 4-methoxyaniline (**2a**) were achieved using water as the reaction medium (Table 2, entry 6). The recovery and reuse experiment showed a total selectivity toward amine **2a** up to the fourth run, although a decrease in conversion was observed as early as in the third run (Table 2, entry 6). Complete conversion and high selectivity were also achieved using an H_2_O/EtOH (9:1) mixture, but conversion dropped significantly in the third run (Table 2, entry 7). Accordingly, the use of an H_2_O/EtOH (1:1) mixture as the reaction medium led to a complete loss of catalytic activity by the second run, with a conversion of only 16%, highlighting the negative impact of EtOH on reactivity (Table 2, entry 8). However, when methanol was used as the reaction medium, complete conversion and highly selective reduction of **1a** to 4-methoxyaniline (**2a**) were maintained up to the fifth run, with only a slight decrease in selectivity observed from the fourth run onward (Table 2, entry 9).

Reducing the amount of NaBH_4_ to 2 equiv or the amount of catalyst to 1 mol % under the same reaction conditions (30 °C for 3 h), a decrease in catalytic activity was observed (Table 2, entries 10 and 11, respectively).

To explain the differences in catalytic activity observed for **Ni@ZrPGly-1** when using water or methanol as the reaction medium, the nature of the nickel species formed upon reduction was investigated. The nanomaterial was reduced using NaBH_4_ in either MeOH or water to promote the formation of reduced Ni-based species, such as Ni or Ni_*x*_B NPs.^34–38^ During this process, the sample changed color from green to dark gray/black (Figure S2), which is consistent with the formation of both aforementioned species. Elemental analysis and SEM-EDX analyses were performed on the reduced catalysts (Table S1 and Figure S3). The presence of boron, uniformly distributed across the samples, was confirmed by EDX mapping, while ICP quantification yielded a Ni/B molar ratio of 2.23 and 2.85 for **Ni@ZrPGly-1R** reduced in MeOH and water, respectively.

XPS analysis of these samples revealed significant modification in the Ni-related core-level spectra compared to the pristine material (**Ni@ZrPGly-1**) (Figure S4, Table S1). Specifically, a low-binding energy XPS peak emerged at ∼853.5 eV and was assigned to nickel boride phases (Ni_2_B, Ni_3_B), in good agreement with literature data for nickel-boride systems.^47^ Concurrently, the component at 855.6 eV, accompanied by the characteristic shakeup satellites in the 861.0–864.0 eV range, was attributed to the spontaneous formation of a surface-oxidized phase (e.g., NiO or Ni­(OH)_2_) induced by air exposure during sample manipulation. The high reactivity of the nickel boride phase is reflected in the relatively low intensity of the peak at 853.5 eV. Unfortunately, the high-resolution XPS spectra do not exhibit any discernible photoelectron signal in the B 1s region corresponding to the B–Ni bond (typically expected at ∼188 eV binding energy). Furthermore, a definitive evaluation of the oxidized boron species remains elusive, as the B–O contribution (∼193 eV BE) severely overlaps with the spectral region of the P 2s core levels. Nonetheless, based on these findings, it can be cautiously inferred that the NaBH_4_ treatment successfully leads to the formation of Ni_*x*_B species. Specifically, the reduction in methanol tends to favor the formation of a boron-rich Ni_2_B phase, whereas reduction in water promotes the generation of a less boron-dense Ni_3_B phase. The morphology and size distribution of Ni_*x*_B nanoparticles in the reduced samples of **Ni@ZrPGly-1** were investigated by STEM (Figure 4). The STEM images of both **Ni@ZrPGly-1R** in MeOH and in water showed the presence of bright nanoparticles on the phosphonate surface, assigned to Ni species based on EDX analysis and attributed to Ni_*x*_B phases. However, reduction in water produced only a few Ni_*x*_B NPs with mean diameters of 19.8 ± 10.2 nm, whereas reduction in MeOH yielded a dense population of Ni_*x*_B nanoparticles with an average diameter of 3.5 ± 1.6 nm, appearing as isolated nanoparticles or small aggregates. Furthermore, the samples stored under wet conditions showed different macroscopic behaviors over time: the sample obtained in MeOH retained its color and texture, while the sample obtained in water quickly turned light gray with an ash-like consistency.

**Figure 4:**
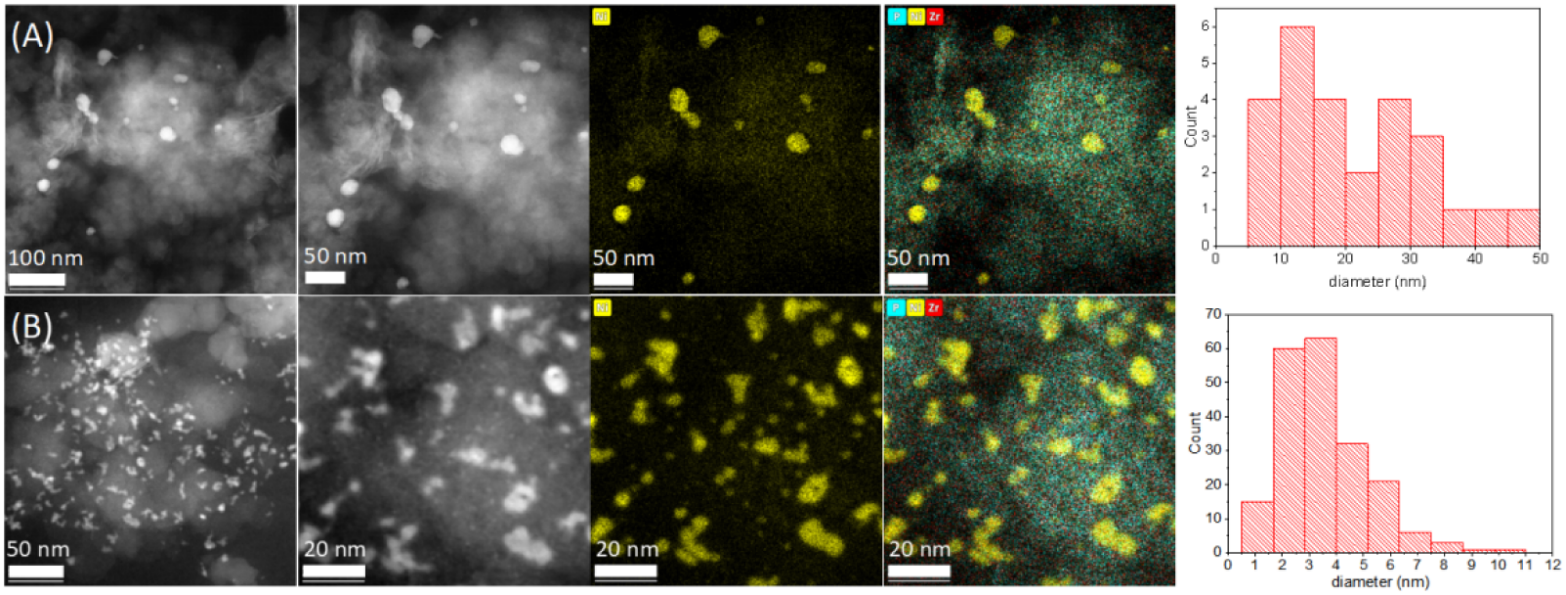
STEM, Ni (yellow), P (cyan), and Zr (red) mapping performed by EDX analysis, and Ni*_x_* B NPs size distribution of **Ni@ZrPGly-1R** reduced by NaBH_4_ in water (A), and in MeOH (B).

The structural stability of **Ni@ZrPGly-1R** reduced in methanol and in water was investigated by XRD, ATR-FTIR, TEM, and ICP-OES analyses collected on the samples recovered after five and four catalytic runs, respectively.

The XRD patterns of both recovered samples (Figure S5) still display the characteristic reflections of the ZrPGly phase, together with a very weak and broad reflection at 18.4 Å associated with the interlayer distance. This suggests that partial exfoliation of the layered structure occurs in the reaction medium and that only limited restacking takes place after catalyst recovery, particularly for the sample recovered after four runs in water. Nevertheless, the preservation of the main diffraction features indicates that the internal structure of the individual layers is substantially preserved. Consistently, the ATR-FTIR spectra of both recovered catalysts (Figure S6) retain the characteristic absorptions of the −COO^–^ and phosphate groups, confirming that the chemical structure of the support is maintained after catalysis.

The extent of nickel leaching into the reaction medium, and the Ni/Zr and P/Zr ratios in the recovered catalysts were evaluated by ICP-OES analysis. As shown in Table 3, the amount of nickel released into solution was very low (0.38–1.40 ppm), corresponding to a leaching of 0.2–0.8% in each run relative to the initial amount of Ni used. After five runs in methanol, the recovered catalyst showed only a slight increase in the Ni/Zr ratio, while the P/Zr ratio was unchanged. TEM images (Figure S7a,b) revealed a well-preserved lamellar morphology and a homogeneous distribution of Ni_*x*_B nanoparticles with average dimensions very similar to those of the fresh catalyst. Only a modest increase in nanoparticle aggregation was observed, which may account for the slight decrease in selectivity detected in the fourth and fifth runs.

**Table 3:** Ni Leaching in the Reaction Medium After Each Cycle, Ni/Zr Ratio and P/Zr Ratio After Use

Medium	run	Ni ppm in solution	Ni leaching (%)^a^	Ni/Zr^b^	P/Zr^c^
H_2_O	1	0.76	0.43		
	2	0.40	0.23		
	3	0.60	0.34		
	4	0.72	0.41	1.23	2.69
MeOH	1	1.40	0.80		
	2	0.56	0.32		
	3	0.46	0.26		
	4	1.13	0.64		
	5	0.38	0.21	1.05	2.56

a Ni leaching (%) represents the amount of nickel released into solution during each run relative to the initial Ni content of the catalyst used.

b The Ni/Zr ratio in pristine **Ni@ZrPGly-1** was 0.90.

c The P/Zr ratio in pristine **Ni@ZrPGly-1** was 2.63.

In contrast, the catalyst recovered after four runs in water exhibited a slightly greater increase in the Ni/Zr ratio and more pronounced morphological changes. TEM image (Figure S7c) confirmed the partial exfoliation of the layered support already observed in the XRD patterns, and also showed the absence of clearly distinguishable nickel-based nanoparticles. These observations suggest that delamination/exfoliation under the reaction conditions leads to a loss of support material, resulting in an apparent increase in the Ni/Zr ratio. Such structural and textural modifications may account for the decrease in catalytic activity observed from the third run onward.

Several studies^48–52^ report that the catalytic performance of nickel-based catalysts is strongly influenced by the amount of nickel immobilized on the support. The activity typically follows a bell-shaped trend, with an optimal loading between 10 and 15% w/w, at which good dispersion of nickel nanoparticles and a maximum fraction of accessible active sites are observed. At low loadings, the metal nanoparticles, although highly dispersed on the support, are largely inaccessible. At high loadings, an increase in nanoparticle size is generally observed, leading to a reduction in catalytic activity.

To verify the dependence of catalytic activity on nickel loading, the performance of **Ni@ZrPGly-1** was compared with that of **Ni@ZrPGly-2** and **Ni@ZrPGly-3**, which have different nickel loadings.

An additional comparison was made using **Ni@ZrP**, which lacks glyphosine groups; in this case, nickel is immobilized and stabilized solely by phosphate groups.

Before the catalytic tests, these reduced samples were analyzed by ICP, SEM/EDX, XPS, STEM and XRD analyses. In line with the previous observations, the ICP, SEM-EDX, and XPS results collectively confirm the formation of Ni_*x*_B species, as summarized in Table S1 and Figures S3 and S4. STEM images (Figure 5) of **Ni@ZrPGly-2R**, where the Ni loading is about 30% w/w lower than in **Ni@ZrPGly-1R**, show the formation of Ni_*x*_B NPs with a mean diameter of about 3.8 ± 2.2 nm (Figure S8), together with a relatively homogeneous distribution of nickel ions, suggesting that not all Ni^2+^ species are reduced. Conversely, in the sample with the lowest nickel content, **Ni@ZrPGly-3R**, a uniform distribution of nickel ions is visible in the STEM-EDX images; only a few Ni_*x*_B NPs with a diameter of about 10 nm were detected as reported in the STEM images of Figure S9. These findings suggest that the formation of Ni_*x*_B NPs is favored at higher nickel loadings, as the number of Ni_*x*_B NPs decreases with decreasing nickel concentration on the solid. As for **Ni@ZrP-R**, in accordance with its low Ni loading, STEM and EDX analyses revealed that nickel is mainly present as Ni^2+^, and only a few Ni_*x*_B NPs were detected with diameters of about 10–50 nm (bright particles in Figure S10).

**Figure 5:**
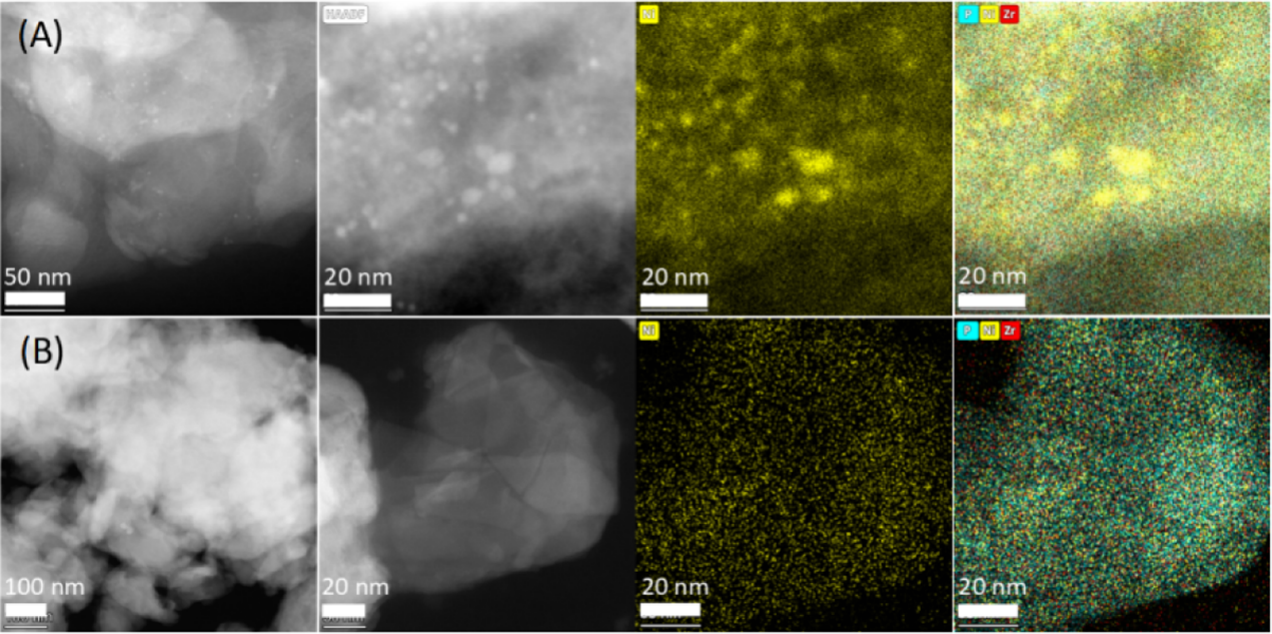
STEM and Ni, P, and Zr mapping performed by EDX analysis of **Ni@ZrPGly-2R** (A), and **Ni@ZrPGly-3R** (B), obtained by reduction with NaBH_4_ in MeOH.

The XRD patterns (Figure S11) did not show any reflections attributable to reduced nickel-based species, which is consistent with both the very small nanoparticle size in **Ni@ZrPGly-1R** and the very low density of Ni-based NPs present in **Ni@ZrPGly-2R** and **Ni@ZrPGly-3R**. Conversely, the (111) and (200) reflections characteristic of the cubic phase of metallic nickel were observed in the **Ni@ZrP-R** sample. In this specific case, a fraction of nickel was present in its metallic state with a particle size sufficiently large to be detected by XRD (Figure S11).

The results of the catalytic activity tests are reported in Table 4.

**Table 4:**
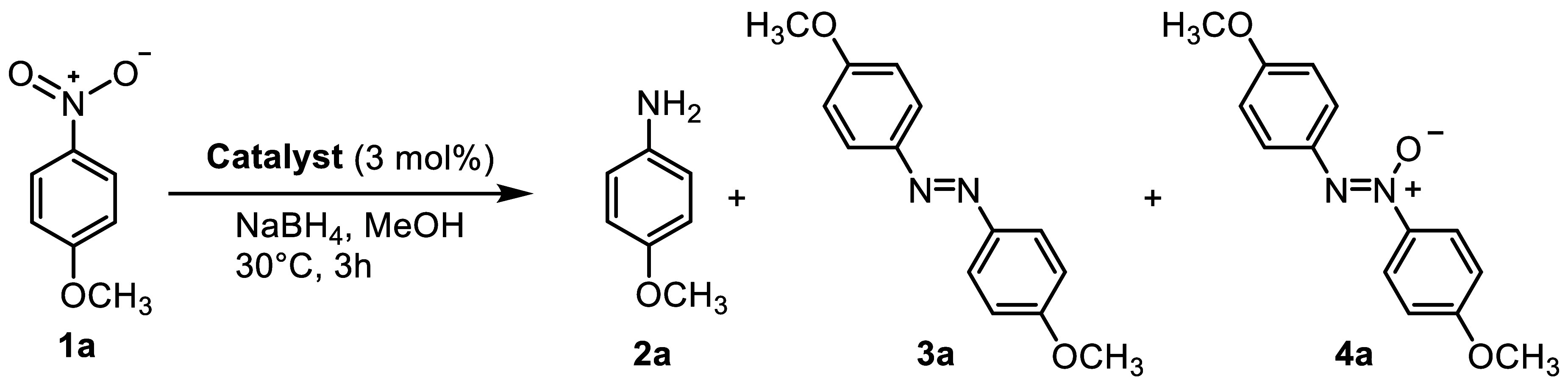
Effect of Nickel Loading and Support Structure on the Catalytic Activity of Ni-Based ZrP Materials in the Reduction of 4-Nitroanisole (**1a**)

Entry^a^	Catalyst	Ni loading w/w%	Conv %^b^	**2a:3a:4a** ^b^
1	**Ni@ZrPGly-1**	12.15	100	100:0:0
2	**Ni@ZrPGly-2**	8.22	99	74:3:23
3	**Ni@ZrPGly-3**	4.63	58	40:2:58
4	**Ni@ZrP**	7.57	18	67:0:33
5	-		8	38:0:62

a Reaction conditions: **1a** (0.2 mmol), Catalyst (3 mol %), NaBH_4_ (3 equiv), MeOH (2 mL) at 30 °C for 3 h.

b Conversion and product distribution were determined by GLC analysis.

As can be seen, both the conversion and the selectivity of the reaction decrease with decreasing nickel loading. In particular, with **Ni@ZrPGly-2** (Table 4, Entry 2), conversion is nearly complete, but the selectivity toward 4-methoxyaniline (**2a**) is only 74%. With **Ni@ZrPGly-3** (Table 4, Entry 3), the conversion drops to 58%, and the azoxy derivative (**4a**) becomes the most abundant product. These results are consistent with literature data,^48–52^ which indicate that lower nickel loading reduces the accessibility of the nickel nanoparticles and, consequently, lowers catalytic activity.

When inorganic zirconium phosphate (ZrP) was used as a support to immobilize nickel ions (**Ni@ZrP**), the catalytic activity was very low, with only 18% conversion and poor selectivity (Table 4, Entry 4). This result aligns with the larger size and agglomeration of the Ni_*x*_B NPs on this support, as shown in the TEM images in Figure S10, and highlights the important role of the organic glyphosine groups on the surface of the zirconium phosphonate lamellae in immobilizing and activating the Ni_*x*_B nanoparticles. The control experiment performed in the absence of any catalyst led to a 8% conversion with a low product selectivity (Table 4, Entry 5). Indeed, despite the evident H_2_ evolution resulting from the methanolysis of NaBH_4_, hydrogen does not react with nitroarenes in the absence of metal catalytic sites capable of activating H–H bonds and promoting hydrogen transfer.^53^

According to the available literature,^52,54–58^ the reduction of nitroaromatic compounds over Ni_*x*_B NP-based catalytic systems has been proposed to proceed via a heterogeneous surface mechanism, where NaBH_4_ generates active Ni–H species responsible for stepwise hydrogen transfer. Two mechanistic pathways^52,54–58^ were recognized: the direct route and the condensation route (Figure 6). In the direct pathway, the aniline product is formed through nitroso and hydroxylamine intermediates, whereas the condensation pathway involves the formation of diazoxy compounds through the condensation of nitroso and hydroxylamine intermediates. The diazoxy derivatives can subsequently undergo further hydrogenation to diazo, hydrazo, and finally to aniline products.

**Figure 6:**
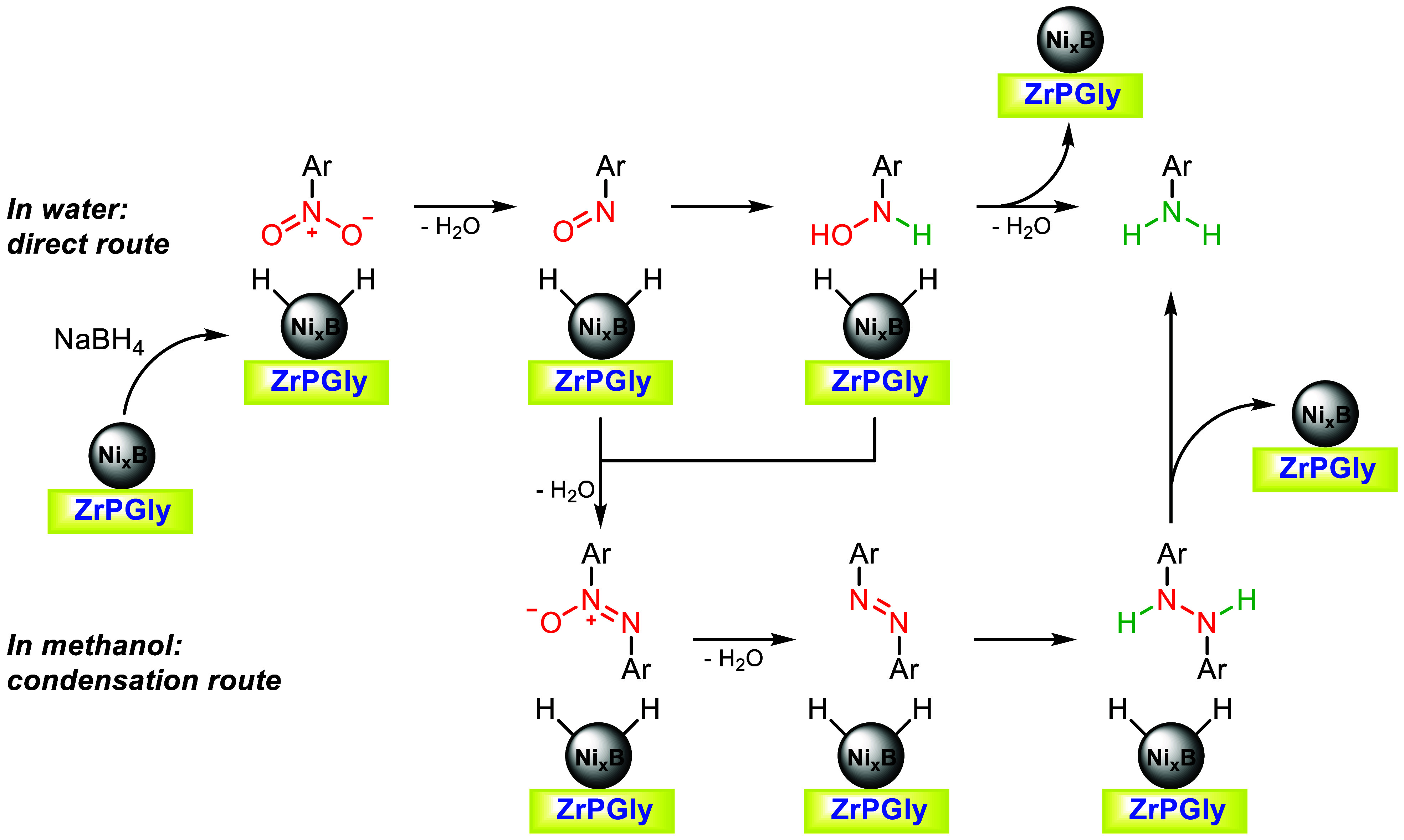
Plausible reaction pathways for reduction of nitroarenes in water (*direct route*) and methanol (*condensation route*).

Density functional studies^58^ suggested that, when water was used as the reaction medium, the direct reduction pathway is energetically more favorable than the condensation pathway, although experimental evidence reported in the literature indicates that both mechanisms may operate depending on the reaction conditions, such as the physicochemical properties of the reaction medium, the reducing agent, the support, and the nature of the catalytically active species.^52,54–57^ In the present work, the observed product distributions are consistent with these literature reports, although they do not allow definitive mechanistic conclusions to be drawn.

When water was used as reaction medium, azoxy- or azo-compounds were never detected, even when the catalytic activity decreased in recycling experiments (Table 2, Entry 6) or when less active catalysts such as **Ni@ZrPGly-2**, **Ni@ZrPGly-3**, or **Ni@ZrP** were used (see Table S1 for supplementary data). These observations are consistent with a reaction pathway predominantly involving direct reduction steps. In contrast, when methanol was used as reaction solvent, condensation products were detected particularly when the catalytic activity decreased as in the last two recycling runs (Table 2, Entry 9) or when less active catalysts were employed (Table 4), suggesting the possible involvement of condensation-type intermediates.

These different product distributions observed in the two solvents may reflect the combined effect of solvent properties and differences in the composition of the catalytically active nanoparticles. Indeed, TEM-EDX and ICP analyses suggest the formation of nickel boride phases with different compositions depending on the reduction medium, with Ni/B ratios consistent with Ni_2_B-type species for the catalyst reduced in methanol and Ni_3_B-type species for that reduced in water.

To further investigate the nature of the catalytic process, a filtration test was performed using the reduction of 4-nitroanisole (**1a**) with the Ni@ZrPGly-1/NaBH_4_ system in MeOH as a model reaction (see Supporting Information). The heterogeneous catalyst was removed by filtration after 15 min (22% conversion; **1a:2a:3a:4a** = 78:6:2:15), and 2 equiv of NaBH_4_ were subsequently added to the filtrate. After further stirring for 3 h, under the optimized reaction conditions, only a limited increase in conversion was observed (28%). An increase in the percentage of azo- and azoxy-compounds was observed, whereas the amount of aniline **2a** did not increase (**1a:2a:3a:4a** = 72:5:3:25). These results together with the reactivity observed in the absence of catalyst (Table 4, entry 5), and the very low Ni leaching into solution (Table 3), support the heterogeneous nature of the catalytic process and are consistent with the possible involvement of a surface-mediated condensation mechanism.

The optimized reaction conditions for the selective reduction of nitroarene **1a** using **Ni@ZrPGly-1** (3 mol %) and NaBH_4_ (4 equiv) in MeOH at 30 °C were then applied to a wide variety of nitroarenes (Table 5). A series of anilines (**2a–2n**) was obtained in high yield and with high selectivity within 1–6 h.

**Table 5:**
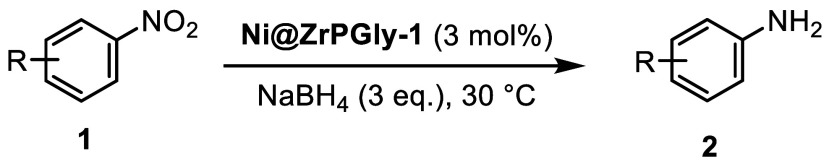

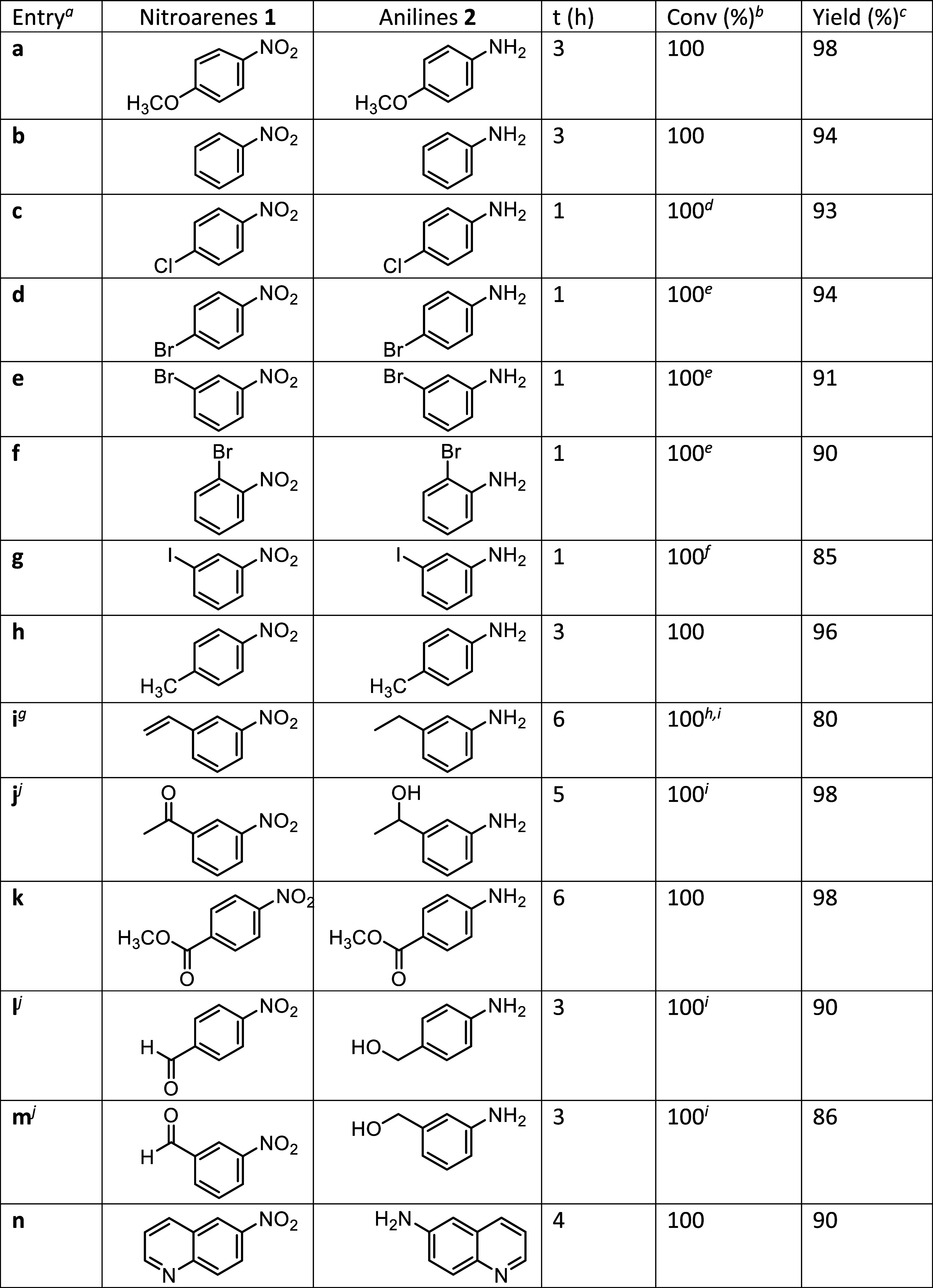
Substrate Scope for the Preparation of Anilines **2a**–**2n** in the Presence of **Ni@ZrPGly-1**

a Reaction conditions: 1 (0.2 mmol), **Ni@ZrPGly-1** (3 mol %), NaBH_4_ (3 equiv), MeOH (2 mL).

b Conversion and product distribution were determined by GLC analysis.

c Yield of isolated products **2**.

d 2% of dehalogenated aniline **2b** was observed.

e 3–4% of dehalogenated aniline **2b** was observed.

f 9% of dehalogenated aniline **2b** was observed.

g 5 eq. of NaBH_4_ were used.

h 5% of diazo-compound **3i** was observed.

i Reduction of vinyl and carbonyl substituent group was observed.

j 4 eq. of NaBH_4_ was used.

It has been reported that the dehalogenation of halogen-substituted substrates can occur during the hydrogenation reaction.^25,32,33,59,60^ For 4-chloronitrobenzene (**1c**) and 2-, 3-, and 4-bromonitrobenzene (**1d**, **1e**, **1f**), only traces (2–4%) of the dehalogenated product was observed, while 4-iodonitrobenzene underwent 9% dehalogenation. As observed in previous studies,^25,32,54^ the presence of sodium borohydride also promoted the reduction of carbonyl groups. In particular, the reduction of 3-nitroacetophenone (**1j**), 4- and 3-nitrobenzaldehyde (**1l**, **1m**) yielded the corresponding 1-hydroxyethyl aniline (**2j**) and hydroxymethyl anilines (**2l**, **2m**), resulting from the reduction of both nitro and carbonyl groups. Remarkably, the reduction of 3-nitrostyrene (**1i**) led to the formation of the corresponding 3-ethylaniline (**2i**), indicating that, together with the reduction of the nitro group, the hydrogenation of the vinyl group also occurs.

As is well-known, the catalytic hydrogenation of alkenes is an important chemical transformation in industrial organic synthesis. High-pressure hydrogen gas is generally used that requires an expensive experimental setup.^19^ Although the use of NaBH_4_ as a reducing agent in the catalytic hydrogenation of alkenes is rather limited, its mild reaction conditions, low cost, and environmentally friendly profile make this approach highly attractive for sustainable chemical transformations.^53,61^

In this context, the unexpected reactivity of **Ni@ZrPGly-1** in reducing the vinyl group in 3-nitrostyrene (**1i**, Table 5) led us to test its catalytic activity in the hydrogenation of alkenes (Table 6). When styrene **5a** was used, hydrogenation of the vinyl group occurred in 4 h at 30 °C using 3 mol % of **Ni@ZrPGly-1** and 3 eq. of NaBH_4_. The solid catalyst was recovered by centrifugation and recycled up to five times without significant loss of catalytic performance (Table 6, Entry **a**). The optimized catalytic protocol was then applied to a variety of alkene substrates. In all cases, the hydrogenation reactions were complete in 4 h, except for dimethoxystilbene (**5d**), which required 40 h at 40 °C due to the poor solubility of the reagent. For 4-chlorostyrene (**5b**), 8% of the dehalogenated product was observed, while hydrogenation of 4-acetoxystyrene (**5c**) yielded the corresponding 4-ethylphenol due to the hydrolysis of the acetoxy group. Additionally, a nonconjugated alkene such as cyclooctene (**5g**) was hydrogenated to the corresponding alkane.

**Table 6:**
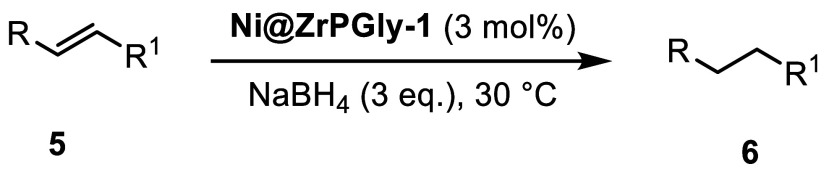

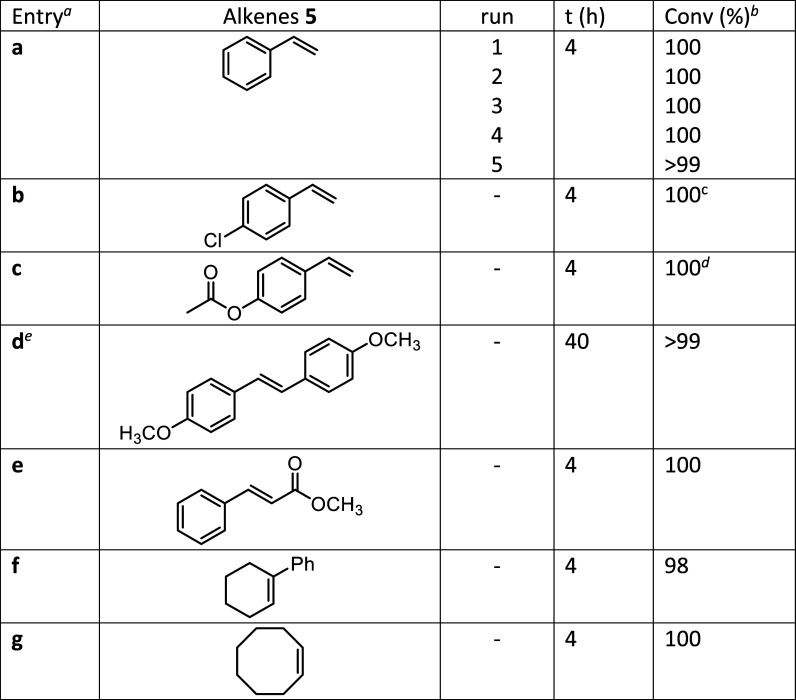
Hydrogenation of Alkenes **5** with NaBH_4_ in the Presence of **Ni@ZrPGly-1**

a Reaction conditions: **5** (0.2 mmol), **Ni@ZrPGly-1** (3 mol %), NaBH_4_ (3 equiv), MeOH (2 mL).

b Conversion and product distribution were determined by GLC and CG-MS analyses.

c 8% of dehalogenated product was observed.

d 4-ethylphenol was obtained.

e At 40 °C.

Tables S2 and S3 report a comparison of the catalytic performance of the present catalyst with commercial Raney Ni or Ni/C and other recently reported Ni-based catalysts for the hydrogenation of nitroarenes and alkenes, respectively. The reaction conditions employed in this work offer significant advantages, including milder operating conditions, lower reaction temperatures, the use of NaBH_4_ as a safe hydrogen source, and comparable or, in several cases, higher product yields. In addition, the very low residual nickel content detected in the reaction solutions and the stability over multiple catalytic runs highlight the great potential of the synthesized **Ni@ZrPGly-1** catalyst for the sustainable catalytic hydrogenation of nitroarenes and alkenes.

## 3 Conclusion

Exfoliated zirconium phosphate carboxy-aminophosphonate (ZrPGly) proved to be an effective support for the immobilization and stabilization of amorphous nickel-based nanoparticles, leading to highly efficient and recyclable catalytic systems for the selective hydrogenation of nitroarenes and alkenes under mild reaction conditions. Among the prepared materials, **Ni@ZrPGly-1**, bearing the highest nickel loading, displayed the best catalytic performance, achieving full conversion and excellent chemoselectivity in the reduction of a broad range of nitroarenes and alkenes using NaBH_4_ as a convenient and sustainable hydrogen source in methanol at 30 °C.

Additional characterization results obtained by ICP, SEM-EDX, TEM, and XPS analyses suggest that the active phase generated during the reduction process consists of Ni_*x*_B-based nanoparticles. Moreover, the results indicate that the composition of the reduced Ni-based phase depends on the reaction medium, with Ni/B ratios consistent with Ni_2_B-type species for catalysts reduced in methanol and Ni_3_B-type species for those reduced in water. These differences, together with the distinct physicochemical properties of the solvents, may contribute to the different catalytic performance observed.

Recycling tests demonstrated the robust nature of the catalyst in methanol, showing high stability of both the support and the Ni-based catalytic species, negligible metal leaching, and sustained catalytic activity over at least five consecutive runs. In contrast, the reduced catalytic activity in aqueous media highlighted the role of the solvent in determining the reactivity and stability of the Ni-based catalytic system. Comparative studies using materials with lower Ni loadings and conventional zirconium phosphate support (**Ni@ZrP**) highlighted the importance of both the high dispersion of Ni_*x*_B nanoparticles and the presence of glyphosine groups in achieving optimal catalytic performance.

Overall, this study demonstrates that **Ni@ZrPGly** nanomaterials represent a cost-effective, reusable, and environmentally benign alternative to noble-metal catalysts for hydrogenation processes, combining the advantages of earth-abundant Ni-based active species with the structural stability and tunability of layered zirconium phosphonate supports. The excellent catalytic results obtained under mild operating conditions underscore the potential of these hybrid nanomaterials for sustainable applications in reductive transformations of synthetic and industrial relevance.

## 4 Experimental Section

### 4.1 Materials

All chemicals were supplied by Sigma-Aldrich and used without additional purification. For the preparation of N,N-bis­(phosphonomethyl)­glycine, the protocol developed by Moedritzer and Irani was followed,^62^ which is detailed in the Supporting Information.

XRD patterns were recorded using a Philips X’Pert PRO MPD diffractometer operating at 40 kV and 40 mA, with a step size of 0.0334° 2θ and a counting time of 40 s per step, using CuKα radiation and an X’Celerator detector. To avoid preferred orientation effects, the powdered samples were packed into a glass holder using a side-loading technique.

An ICP Varian Liberty inductively coupled plasma-optical emission spectrometer (ICP-OES) with axial injection was used to determine the content of Zr, P, Ni, and B. To dissolve the solid samples without nickel, a 3 M HF solution was applied. For the nickel-bearing solids, the matrix was first treated with 3 M HF, followed by the addition of a suitable volume of aqua regia to achieve complete dissolution. For the determination of the Ni/B molar ratio, samples were digested with aqua regia and the resulting solution were filtered before the ICP-OES analysis.

A TALOS F200X G2 instrument (Thermo-Fisher Scientific) was utilized to collect High-Angle Annular Dark Field (HAADF) images via Scanning Transmission Electron Microscopy (STEM). Operating with a high-brightness Field Emission Gun (X-FEG, 80–200 keV), this microscope features four in-column SDD Super-X detectors. These detectors enabled energy-dispersive X-ray spectroscopy (EDX) to map the elemental composition of the NPs. Lastly, the average diameter and size distribution of the NPs were calculated by statistical analysis of STEM images using the ImageJ software package.

A 400 MHz Bruker DRX-ADVANCE spectrometer was used to record ^1^H NMR spectra.

Morphological and elemental investigations via SEM and EDX were carried out at the LUNA Laboratory (Department of Physics and Geology, University of Perugia) employing a Zeiss LEO 1525 FE SEM. This system features an Angle selective Backscattered (AsB) detector designed for high-energy backscattered electron imaging. To prepare the specimens, powder samples were deposited on aluminum stub precoated with double-sided conductive carbon tape.

Attenuated total reflection-Fourier transform infrared (ATR-FTIR) spectra were recorded using a JASCO FT/IR-4X spectrometer. The spectra were collected in the range 500–4000 cm^–1^ with a resolution of 4 cm^–1^ and 400 accumulated scans.

The X-ray photoelectron spectrometry (XPS) analysis was performed using a Thermo Fisher Nexsa G1 spectrometer equipped with a monochromatic Al Kα source (1486.6 eV). All spectra were collected at a takeoff angle of 90°, with an analyzed spot area of approximately 0.4 mm. Survey spectra were acquired with a step size of 1.0 eV and an analyzer pass energy of 200 eV, whereas high-resolution spectra were acquired with a step size of 0.1 eV (0.05 eV for C 1s and O 1s) and a pass energy of 20 eV. The binding energy scale was calibrated by setting the C 1s peak corresponding to C–C/C–H bonds at 284.8 eV. A charge neutralizer was employed to compensate for surface charging effects during the measurements. Peak fitting was performed using CasaXPS software after Shirley background subtraction and employing a LA(1.643) peak shape.

GLC analyses were performed using a Hewlett-Packard HP 5890A gas chromatograph equipped with a DB-35MS capillary column (30 m × 0.53 mm), a flame ionization detector (FID), and hydrogen as the carrier gas. Gas chromatography–electron impact mass spectrometry (GC-EIMS) measurements were performed using a Hewlett-Packard HP 6890N Network GC system coupled with a 5975 Mass Selective Detector equipped with an electron impact ionization source operating at 70 eV. Thin-layer chromatography (TLC) analyses were performed on silica gel 60 F_254_ aluminum plates (Fluka).

Purification of the products was carried out by column chromatography on silica gel (230–400 mesh) using hexane/ethyl acetate mixtures (98:2–70:30) as eluents. Anilines **2a–n** and alkanes **6a–g** are known compounds (see Supporting Information).

### 4.2 Preparation of Ni Nanomaterial

#### 4.2.1 Synthesis of ZrPGly

The ZrPGly sample, with composition Zr_2_(PO_4_)­H_5_[(O_3_PCH_2_)_2_NCH_2_COO]_2_·H_2_O, was synthesized according to the procedure described previously.^29^ Two precursor solutions were prepared. Solution A was obtained by dissolving *N*,*N*-bis­(phosphonomethyl)­glycine (9 mmol) in 93 mL of water, followed by the addition of 6 mL of 1 M phosphoric acid (molar ratio = 1.5). Solution B was prepared by dissolving zirconium oxychloride octahydrate (1.93 g, 5.9 mmol) in 20.4 mL of 2.9 M hydrofluoric acid (59 mmol; HF/Zr^4+^ molar ratio = 10). The two solutions were combined in a 500 mL Teflon bottle (final P/Zr molar ratio = 4) and heated at 90 °C. After 3 days, the resulting solid was collected by filtration, washed with deionized water, and dried at 60 °C for 24 h. The empirical formula was Zr_2_P_5_O_21_C_8_N_2_H_19_ (FW = 816, IEC = 3.67 mmol/g)

#### 4.2.2 Preparation of ZrPGly Dispersion

250 mg of ZPGly (0.3 mmol) was suspended in 25 mL of deionized water and then 9.3 mL of n-propylamine 0.1 M (0.93 mmol corresponding to 100% of IEC) were added under vigorous magnetic agitation. The resulting colloidal dispersion was maintained at room temperature under stirring for 24 h. Then it was acidified with 1 M HCl solution until pH 2 and stirred for an additional 2 h to remove the amine and regenerate the fully protonated solid. The resulting gelatinous solid (gZrPGly) was collected by ultracentrifugation (13000 rpm, 10 min) and washed three times with deionized water until pH 6. Water (5 mL) was added and a homogeneous dispersion of gZrPGly was obtained.

#### 4.2.3 Preparation of Ni@ZrPGly-1

To the dispersion of gZrPGly in 5 mL of H_2_O, a solution of Ni­(OAc)_2_·4H_2_O (229.7 mg, 0.92 mmol, 200% IEC) in 5 mL of deionized water was added slowly drop by drop under stirring. The dispersion was left under continuous stirring for 48 h at ambient temperature to ensure ion exchange. The Ni-exchanged solid was recovered by ultracentrifugation (13000 rpm, 10 min), washed with water (2 × 5 mL) and dried at 60 °C for 24 h.

#### 4.2.4 Preparation of Ni@ZrPGly-2

To the dispersion of gZrPGly in 5 mL of H_2_O, a solution of Ni­(OAc)_2_·4H_2_O (114.85 mg, 0.46 mmol, 100% IEC) in 5 mL of deionized water was added slowly drop by drop under stirring. The dispersion was left under continuous stirring for 48 h at ambient temperature to ensure ion exchange. The Ni-exchanged solid was recovered by ultracentrifugation (13000 rpm, 10 min), washed with water (2 × 5 mL) and dried at 60 °C for 24 h.

#### 4.2.5 Preparation of Ni@ZrPGly-3

To the dispersion of ZrPGly nanosheets in 5 mL of H_2_O, a solution of Ni­(OAc)_2_·4H_2_O (57.42 mg, 0.23 mmol, 50% IEC) in 5 mL of deionized water was added slowly drop by drop under stirring. The dispersion was left under continuous stirring for 48 h at ambient temperature to ensure ion exchange. The Ni-exchanged solid was recovered by ultracentrifugation (13000 rpm, 10 min), washed with water (2 × 5 mL) and dried at 60 °C for 24 h.

#### 4.2.6 Synthesis of αZrP

α-ZrP, of formula α-Zr­(HPO_4_)_2_H_2_O, was prepared by using the direct precipitation method in the presence of oxalic acid (FW = 301, IEC = 6.64 mmol/g).^63^

#### 4.2.7 Preparation of Ni@ZrP

250 mg of ZrP (0.83 mmol) was suspended in 20 mL of deionized water and then 8.3 mL of n-propylamine 0.1 M (0.83 mmol corresponding to 50% of IEC) were added under vigorous magnetic stirring. The dispersion was maintained at room temperature under stirring for 24 h. To the dispersion of ZrP nanosheets, a solution of Ni­(OAc)_2_·4H_2_O (103.27 mg, 0.415 mmol, 50% IEC) in 10 mL of deionized water was added slowly drop by drop under stirring. The dispersion was left under continuous stirring for 48 h at ambient temperature to ensure ion exchange. The Ni-exchanged solid was recovered by ultracentrifugation, washed with water (2 × 5 mL) and dried at 60 °C for 24 h.

#### 4.2.8 Reduction of Ni­(II)-Exchanged Nanocatalyst

To a suspension of Ni­(II)-exchanged nanocatalyst (50 mg) in methanol (1 mL), NaBH_4_ (4 equiv. relative to Ni content) was added under magnetic stirring. After complete addition, the mixture was stirred for 1 h at room temperature. The resulting solids (hereafter **Ni@ZPGly-1R**, **-2R**, **-3R**, and **Ni@ZrP-R**) were recovered by ultracentrifugation, washed with methanol (2 × 0.5 mL), and stored in methanol for subsequent characterization.

### 4.3 Catalytic Application

#### 4.3.1 General Procedure for the Catalytic Hydrogenation of Nitroarenes

In a 2.5 mL steel vial, **Ni@ZrPGly-1** catalyst (3 mol %), nitroarene **1** (0.2 mmol), NaBH_4_ (3–5 equiv), and MeOH (2 mL) were placed. The resulting mixture was stirred at 30 °C for 1–6 h (Table 5). Upon completion of the reaction, the catalyst was recovered by centrifugation, washed with MeOH (2 × 1 mL), and reused as such in the subsequent catalytic cycle. The methanol solution was concentrated under reduced pressure, and the resulting residue was dissolved in ethyl acetate (5 mL). The organic phase was washed with water (2 × 2 mL), dried over anhydrous sodium sulfate, and concentrated under reduced pressure.

#### 4.3.2 General Procedure for the Catalytic Hydrogenation of Alkenes

In a 2.5 mL steel vial, **Ni@ZrPGly-1** catalyst (3 mol %), alkene **5** (0.2 mmol), NaBH_4_ (3–4 equiv), and MeOH (2 mL) were placed. The resulting mixture was stirred at 30–40 °C for 4–40 h (Table 6). Upon completion of the reaction, the catalyst was recovered by centrifugation, washed with MeOH (2 × 1 mL), and reused as such in the subsequent catalytic cycle. The methanol solution was concentrated under reduced pressure, and the resulting residue was dissolved in ethyl acetate (5 mL). The organic phase was washed with water (2 × 2 mL), dried over anhydrous sodium sulfate, and concentrated under reduced pressure. For volatile alkane products, diethyl ether (5 mL) was added to the methanol solution, and the resulting organic mixture was washed with water (2 × 2 mL). The diethyl ether layer was dried over anhydrous sodium sulfate and directly analyzed by GC–MS.

## Supplementary Material



## Notes

Additional data for Ni nanomaterials; comparison of the catalytic activity of Ni@ZrPGly-1 with other Ni catalysts; characterization data for all prepared compounds; copies of the ^1^HNMR spectra and/or GC-MS analysis (PDF)
