# Supplementary material for: Zirconium Carboxy-Aminophosphonate Nanosheets Loaded with Nickel-Boride Nanoparticles as an Efficient and Recyclable Catalyst for Nitroarene and Alkene Hydrogenation
Source: ACS Omega. 2026 Jul 7;11(28):42319–33. doi: 10.1021/acsomega.6c02980 (PMC13393373; doi:10.1021/acsomega.6c02980)
Supplement: Supplementary file 1 [file ao6c02980_si_001.pdf]

SUPPORTING INFORMATION

Zirconium Carboxy-Aminophosphonate Nanosheets Loaded with Nickel-boride Nanoparticles as an Efficient and Recyclable Catalyst for Nitroarene and Alkene Hydrogenation

Martina Pancotto,[a] Anna Donnadio[b], Beatrice Muzzi[c], Morena Nocchetti,[b]* Oriana Piermatti[a]*

[a]*Università degli studi di Perugia, Dipartimento di Chimica Biologia e Biotecnologie, Via Elce di Sotto 8, 06123 Perugia, Italy*

[b]*Università degli studi di Perugia, Dipartimento di Scienze Farmaceutiche, Via del Liceo 1, 06123 Perugia, Italy*

[c]*Institute of Chemistry of Organometallic Compounds—C.N.R., 50019 Florence, Italy*

oriana.piermatti@unipg.it

Table of Contents:

1. General Remarks S1
2. Synthesis of N,N-bis(phosphonomethyl)glycine S2
3. Filtration test S2
4. Additional data for Ni Nanomaterials S3
5. Comparison of catalytic activity of **Ni@ZrPGly-1** with other Ni catalysts S9
6. Characterization data for all prepared compounds S10
7. Copies of the ^{1}HNMR spectra and/or GC-MS analysis S14

General remarks

All chemicals were supplied by Sigma-Aldrich and used without additional purification. For the preparation of N,N-bis(phosphonomethyl)glycine, the protocol developed by Moedritzer and Irani was followed (J. Org. Chem. 1966, 31, 1603−1607)

XRD patterns were recorded using a Philips X'Pert PRO MPD diffractometer operating at 40 kV and 40 mA, with a step size of 0.0334° 2θ and a counting time of 40 s per step, using CuKα radiation and an X'Celerator detector. To avoid preferred orientation effects, the powdered samples were packed into a glass holder using a side-loading technique.

An ICP Varian Liberty inductively-coupled plasma-optical emission spectrometer (ICP-OES) with axial injection was used to determine the content of Zr, P, Ni, and B. To dissolve the solid samples without nickel, a 3 M HF solution was applied. For the nickel-bearing solids, the matrix was first treated with 3 M HF, followed by the addition of a suitable volume of aqua regia to achieve complete dissolution. For the determination of the Ni/B molar ratio, samples were digested with aqua regia and the resulting solution were filtered before the ICP-OES analysis.

A TALOS F200X G2 instrument (Thermo-Fisher Scientific) was utilized to collect High-Angle Annular Dark Field (HAADF) images via Scanning Transmission Electron Microscopy (STEM). Operating with a high-brightness Field Emission Gun (X-FEG, 80–200 keV), this microscope features four in-column SDD Super-X detectors. These detectors enabled Energy-dispersive X-ray spectroscopy (EDX) to map the elemental composition of the NPs. Lastly, the average diameter and size distribution of the NPs were calculated by statistical analysis of STEM images using the ImageJ software package.

A 400 MHz Bruker DRX-ADVANCE spectrometer was used to record ^{1}H-NMR and ^{13}C-NMR spectra at frequencies of 400 MHz and 100.6 MHz, respectively.

Morphological and elemental investigations via SEM and EDX were carried out at the LUNA Laboratory (Department of Physics and Geology, University of Perugia) employing a Zeiss LEO 1525 FE SEM. This system features an Angle selective Backscattered (AsB) detector designed for high-energy backscattered electron imaging. To prepare the specimens, powder samples were deposited on aluminum stub pre-coated with double-sided conductive carbon tape.

Attenuated total reflection-Fourier transform infrared (ATR-FTIR) spectra were recorded using a JASCO FT/IR-4X spectrometer. The spectra were collected in the range 500–4000 cm^{-1} with a resolution of 4 cm^{-1} and 400 accumulated scans.

The X-ray photoelectron spectrometry (XPS) analysis was performed using a Thermo Fisher Nexsa G1 spectrometer equipped with a monochromatic Al Kα source (1486.6 eV). All spectra were collected at a take-off angle of 90°, with an analyzed spot area of approximately 0.4mm. Survey spectra were acquired with a step size of 1.0 eV and an analyzer pass energy of 200 eV, whereas high-resolution spectra were acquired with a step size of 0.1 eV (0.05 eV for C 1s and O 1s) and a pass energy of 20 eV. The binding energy scale was calibrated by setting the C 1s peak corresponding to C–C/C–H bonds at 284.8 eV. A charge neutralizer was employed to compensate for surface charging effects during the measurements. Peak fitting was performed using CasaXPS software after Shirley background subtraction and employing a LA(1.643) peak shape.

GLC analyses were performed using a Hewlett-Packard HP 5890A gas chromatograph equipped with a DB-35MS capillary column (30 m × 0.53 mm), a flame ionization detector (FID), and hydrogen as the carrier gas. Gas chromatography–electron impact mass spectrometry (GC-EIMS) measurements were performed using a Hewlett-Packard HP 6890N Network GC system coupled with a 5975 Mass Selective Detector equipped with an electron impact ionization source operating at 70 eV. Thin-layer chromatography (TLC) analyses were performed on silica gel 60 F_{254} aluminum plates (Fluka). Purification of the products was carried out by column chromatography on silica gel (230–400 mesh) using hexane/ethyl acetate mixtures (98:2–70:30) as eluents. Anilines **2a–n** and alkanes **6a–g** are known compounds.

Synthesis of N,N-bis(phosphonomethyl)glycine

N,N-Bis(phosphonomethyl)glycine $(H_2O_3PCH_2)_2NCH_2COOH$ was synthesized following the procedure of Moedritzer and Irani (*J. Org. Chem.* **1966**, *31*, 1603−1607). In brief, 67 mmol of H_2NCH_2COOH and 11 g of H_3PO_3 (133 mmol) were dissolved in 50 mL of 6 M HCl and heated to reflux. A suspension of 8 g of paraformaldehyde (266 mmol) in 10 mL of water was then added dropwise over 2 h. After the addition was complete, the reaction mixture was refluxed for an additional hour, followed by evaporation of the solvent. The crude product was treated with 2-propanol to yield a white solid, which was collected by vacuum filtration and dried in an oven at 60 °C.

Filtration Test

In a steel vial of 2.5 mL, **Ni@ZrPGly-1** (3 mol%), alkene **2a** (0.2 mmol), $NaBH_4$ (1 equiv.) and MeOH (2 mL) were placed. The reaction mixture was stirred at 30 °C for 15 min. The mixture was then rapidly filtered through Celite to remove the heterogeneous catalyst, 2 equiv. of $NaBH_4$ were subsequently added and the filtrate was stirred under the optimized reaction conditions for an additional 3 h.
The distribution of products was determined by GC analyses.

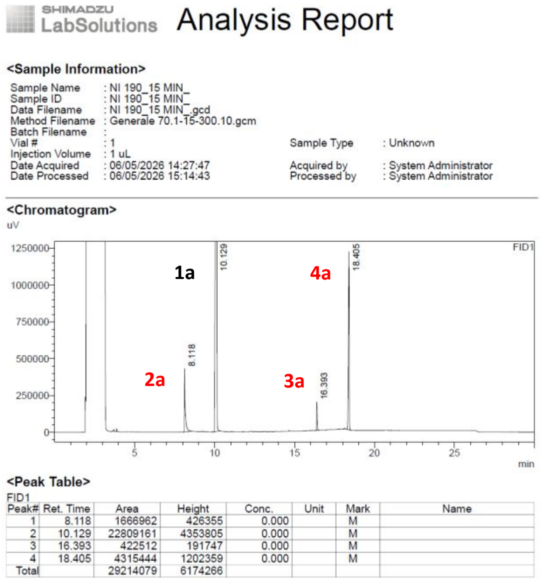
SHIMADZU LabSolutions Analysis Report

<Sample Information>

Sample Name : NI 190_15 MIN_
Sample ID : NI 190_15 MIN_
Data Filename : NI 190_15 MIN_.gcd
Method Filename : Generale 70.1-15-300.10.gcm
Batch Filename :
Vial # : 1
Injection Volume : 1 uL
Date Acquired : 06/05/2026 14:27:47
Date Processed : 06/05/2026 15:14:43
Sample Type : Unknown
Acquired by : System Administrator
Processed by : System Administrator

<Chromatogram>

<Peak Table>

FID1

Peak#	Ret. Time	Area	Height	Conc.	Unit	Mark	Name
1	8.118	1666962	426355	0.000		M	
2	10.129	22809161	4353805	0.000		M	
3	16.393	422512	191747	0.000		M	
4	18.405	4315444	1202359	0.000		M	
Total		29214079	6174266				

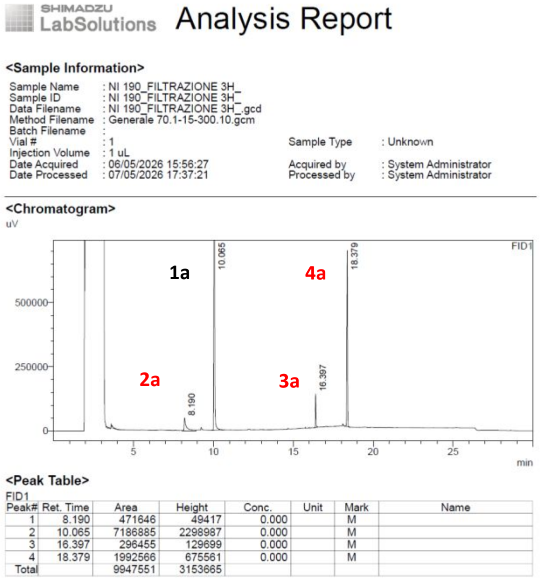
SHIMADZU LabSolutions Analysis Report

<Sample Information>

Sample Name : NI 190_FILTRAZIONE 3H_
Sample ID : NI 190_FILTRAZIONE 3H_
Data Filename : NI 190_FILTRAZIONE 3H_.gcd
Method Filename : Generale 70.1-15-300.10.gcm
Batch Filename :
Vial # : 1
Injection Volume : 1 uL
Date Acquired : 06/05/2026 15:56:27
Date Processed : 07/05/2026 17:37:21
Sample Type : Unknown
Acquired by : System Administrator
Processed by : System Administrator

<Chromatogram>

<Peak Table>

FID1

Peak#	Ret. Time	Area	Height	Conc.	Unit	Mark	Name
1	8.190	471646	49417	0.000		M	
2	10.065	7186885	2298987	0.000		M	
3	16.397	296455	129699	0.000		M	
4	18.379	1992566	675561	0.000		M	
Total		9947551	3153665				

Reaction time 15 min:
1a:2a:3a:4a = 78:6:2:15

Additional 3h after filtration:
1a:2a:3a:4a = 72:5:3:20

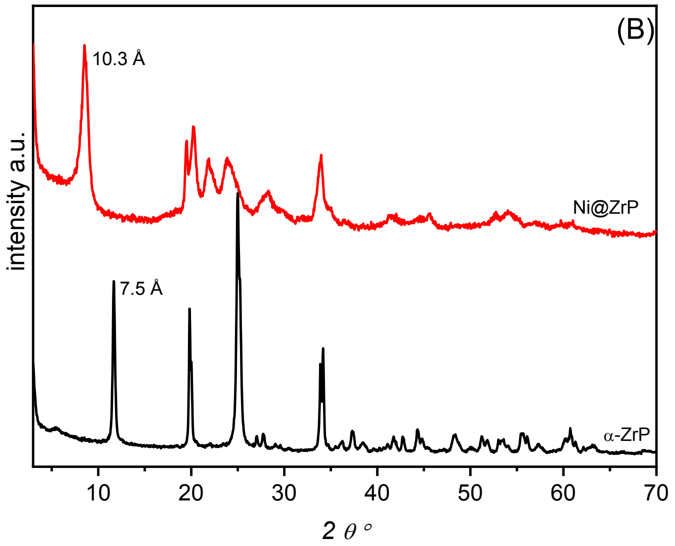

Figure S1. XRD patterns of α-ZrP (black), and Ni@ZrP (red) (B).

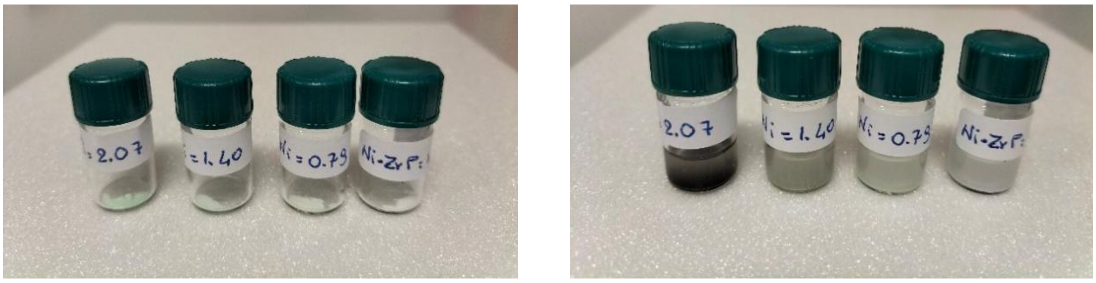

(a) **(b)**

Figure S2. Ni-catalysts powder before reduction **(a)**; Ni catalysts dispersion in MeOH after reduction with $NaBH_4$ **(b)**.

Table S1. Molar fraction of Ni and B in the reduced catalysts, before the reaction. The XPS Binding Energy (BE) are reported for the nickel species.

Sample	NixBy	Ni/B	NiB species	XPS BE (eV) (Ni^0)	XPS BE (eV) (Ni^{2+})
**Ni@ZrPGly-1R**	$Ni_{0.69}B_{0.31}$	2.23	Ni_2B	853.45	855.55
**Ni@ZrPGly-1R** H_2O	$Ni_{0.74}B_{0.26}$	2.85	Ni_3B	853.59	855.98
**Ni@ZrPGly-2R**	$Ni_{0.64}B_{0.36}$	1.78	Ni_2B	853.18	855.79
**Ni@ZrPGly-3R**	$Ni_{0.68}B_{0.32}$	2.13	Ni_2B	853.38	855.57
**Ni@ZrP-R**	$Ni_{0.46}B_{0.53}$	0.87	NiB	853.63	856.43

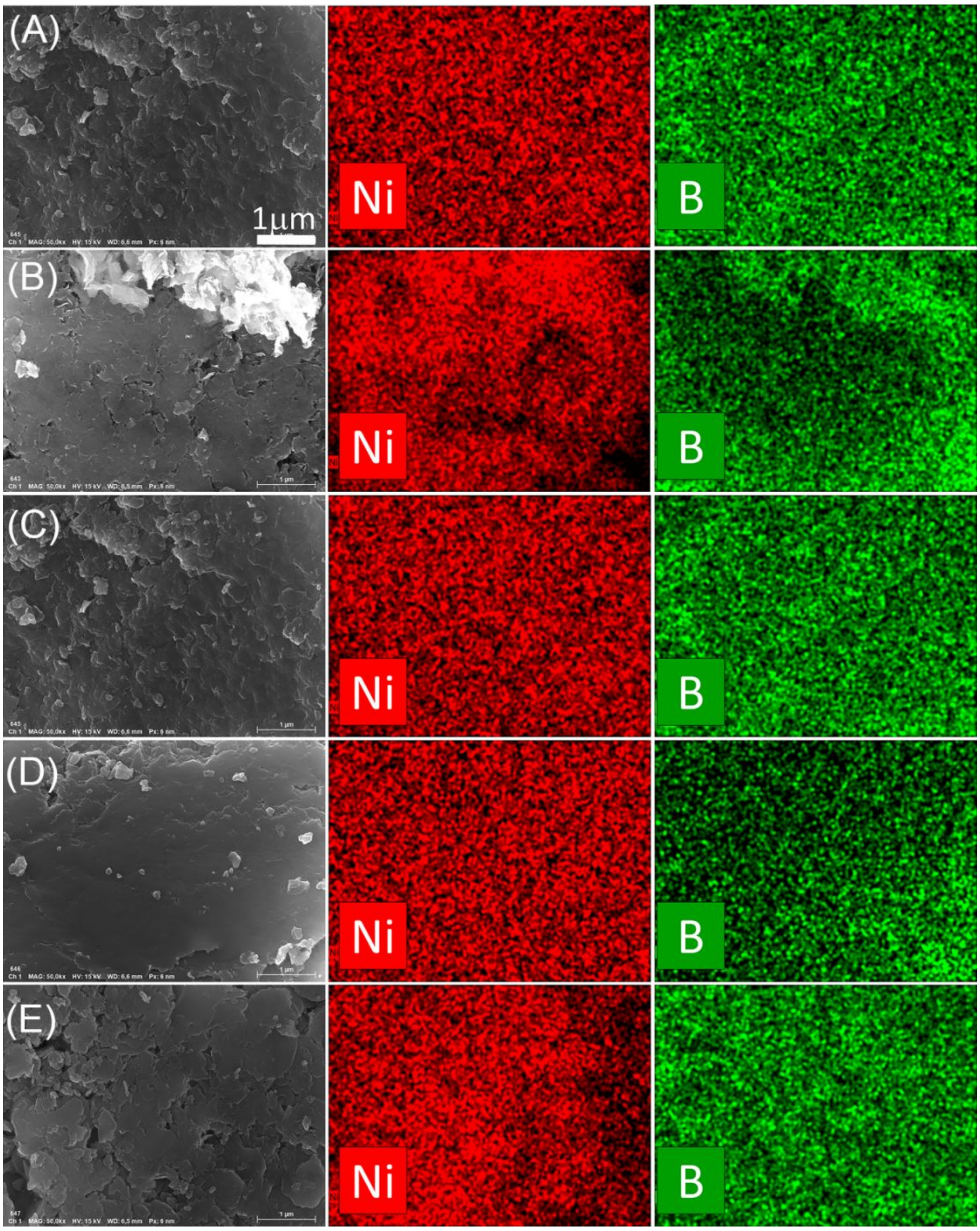

Figure S3. SEM and EDX analysis showing the distribution of Ni and B of: **Ni@ZrPGly-1R** reduced in MeOH (A), **Ni@ZrPGly-1R** reduced in H_2O (B), **Ni@ZrPGly-2R** (C), **Ni@ZrPGly-3R** (D) and **Ni@ZrP-R** (E), reduced in MeOH. The scale bar in the SEM micrograph of (A) applies to all micrographs and EDX mapping panels.

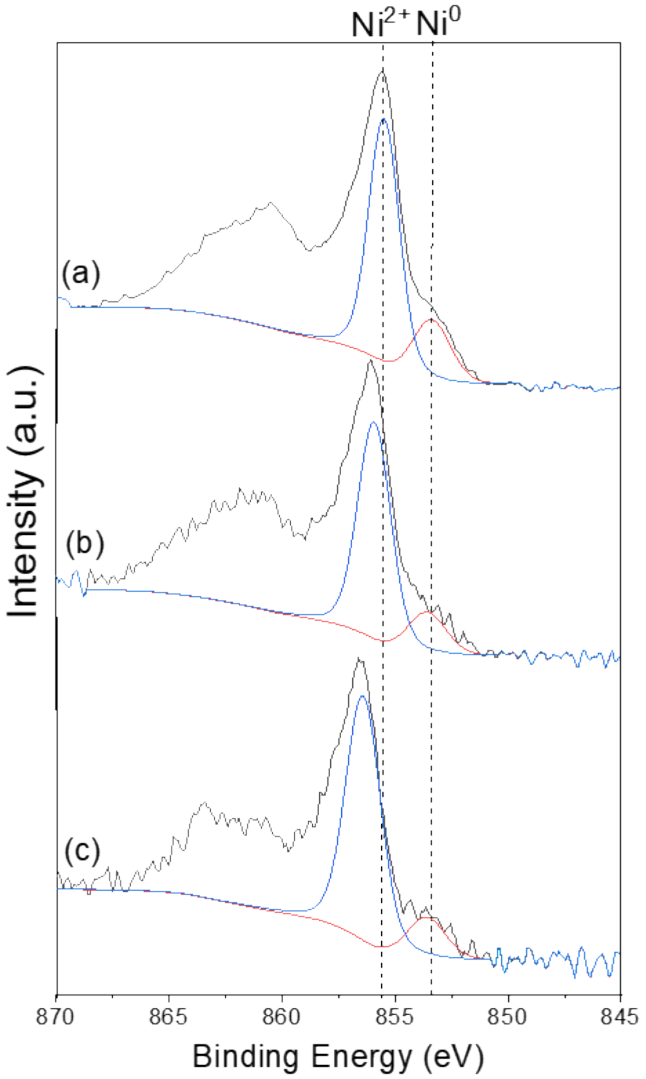

Figure S4. XPS of Ni in **Ni@ZrPGly-1R** in MeOH (a), **Ni@ZrPGly-1R** in H_2O (b), **Ni@ZrP-R** in MeOH(c).

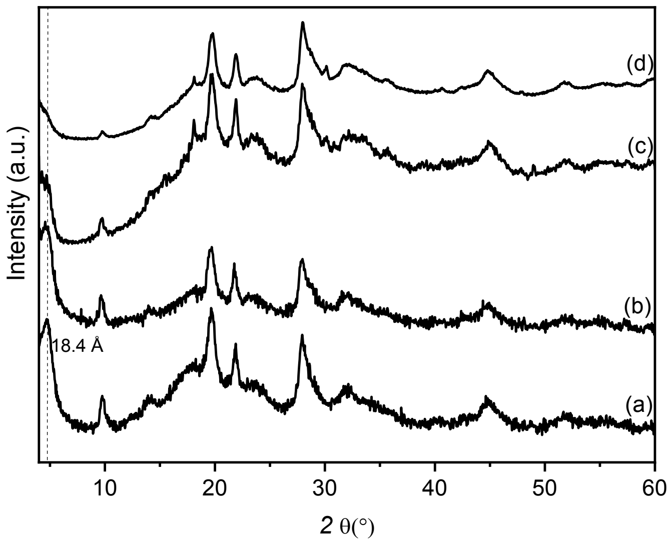

Figure S5. XRD patterns of **Ni@ZrPGly-1** (a), **Ni@ZrPGly-1R** in MeOH (b), **Ni@ZrPGly-1R** in MeOH after five catalytic runs (c) and **Ni@ZrPGly-1R** in H_2O after four catalytic runs (d).

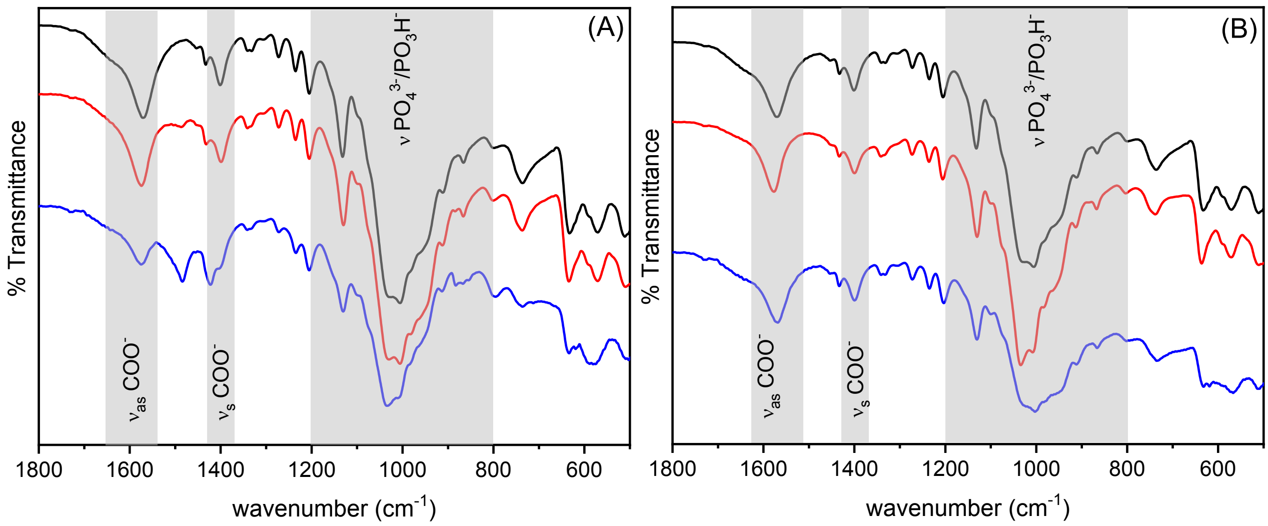

Figure S6. ATR-FTIR spectra of **Ni@ZrPGly-1R** (red line) and **Ni@ZrPGly-1R** after the catalytic runs (blue line) reduced in methanol (A) and water (B) compared with **Ni@ZrPGly-1** (black line).

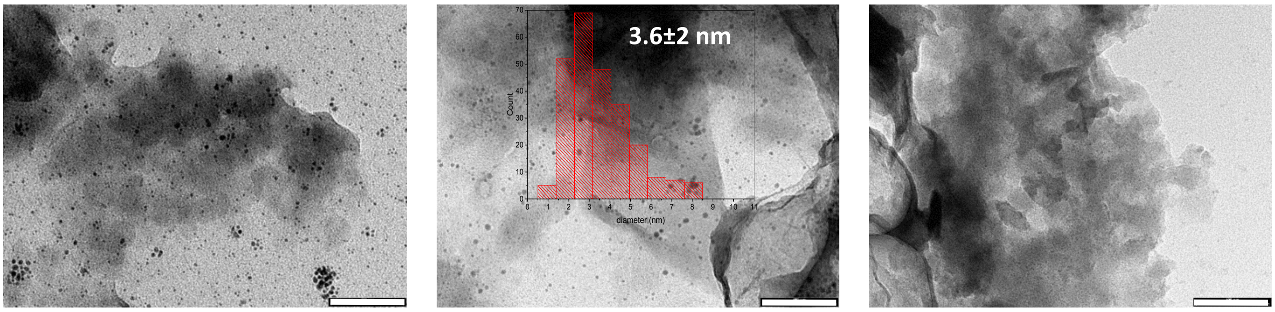

(a) **(b)** **(c)**

Figure S7. TEM[1] image of **Ni@ZrPGly-1R** before catalysis **(a)**; TEM image and Ni_xB particle size distribution of **Ni@ZrPGly-1R** after 5 runs in MeOH **(b)**; TEM image of **Ni@ZrPGly-1R** after 4 runs in H_2O **(c)**. The scale bar in the TEM micrographs corresponds to 100 nm.

[1]Transmission electron microscopy (TEM) analysis was carried out by using a Philips 208 transmission electron microscope, operating at an accelerating voltage of 100 kV. Powders were diluted in methanol, then supported on copper grids (200 mesh) precoated with Formvar carbon films and quickly dried.

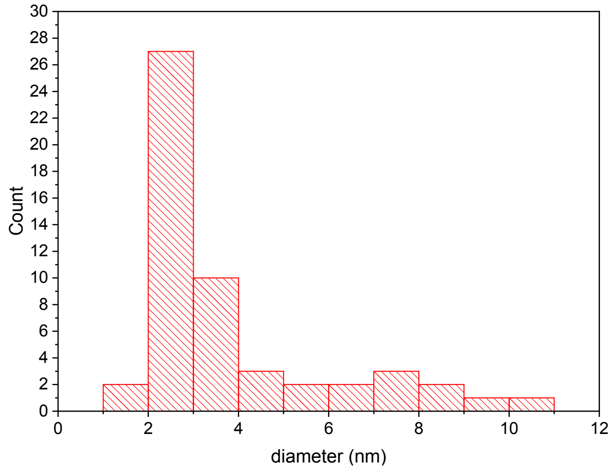

Figure S8. Ni_xB NPs size distribution for Ni@ZrPGly-2R in MeOH.

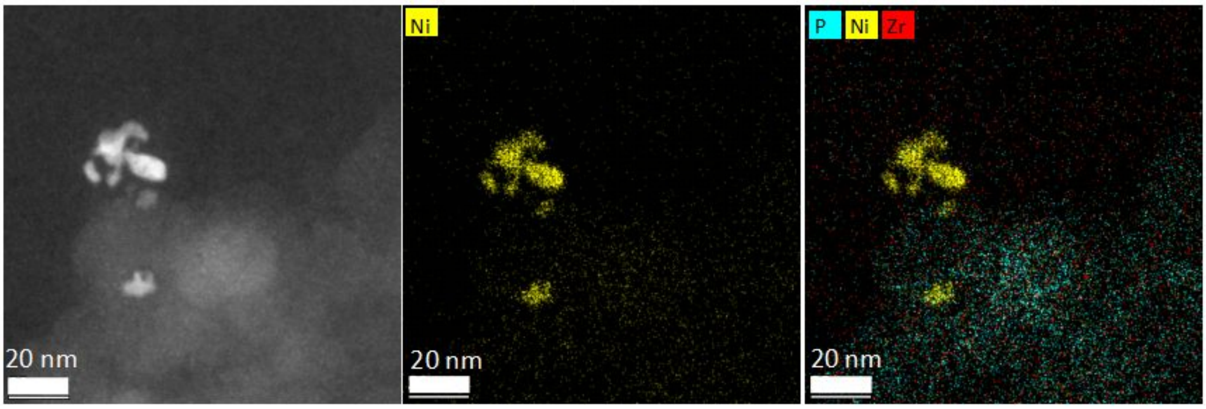

Figure S9. STEM and Ni, P, and Zr mapping performed by EDX analysis for **Ni@ZrPGly-3R**, obtained by reduction with $NaBH_4$ in MeOH

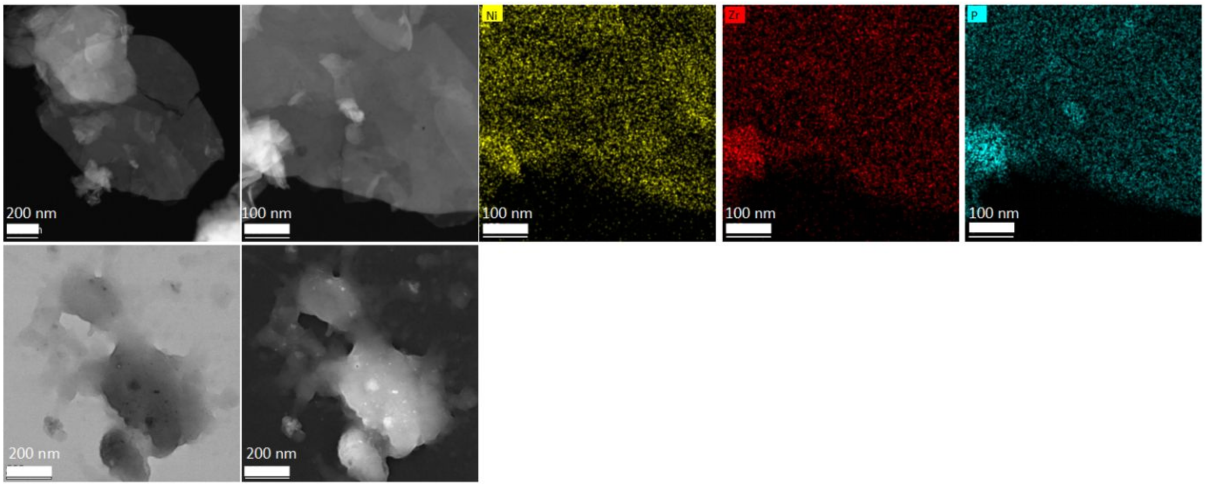

Figure S10. STEM and Ni, P, and Zr mapping performed by EDX analysis for **Ni@ZrP-R**, obtained by reduction with $NaBH_4$ in MeOH.

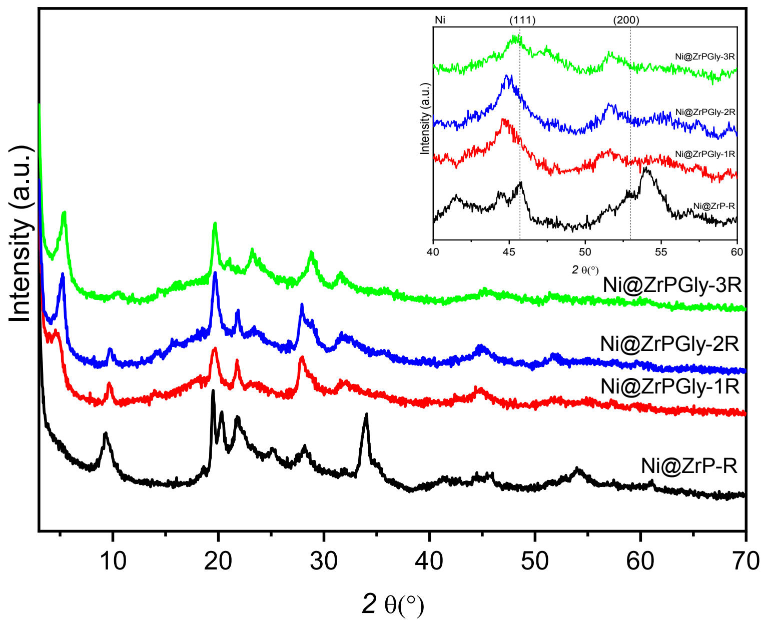

Figure S11. XRD spectra of Ni@ZrP-R (black), Ni@ZrPGly-1R (red), Ni@ZrPGly-2R (blue), and Ni@ZrPGly-3R (green). The inset shows an enlargement of the 2θ region from 40° to 60°, highlighting the (111) and (200) reflections of the cubic phase of metallic nickel.

Table S2. Effect of nickel loading and support structure on the catalytic activity of Ni-based ZrP materials in the reduction of 4-nitroanisole (**1a**) in water as reaction medium.

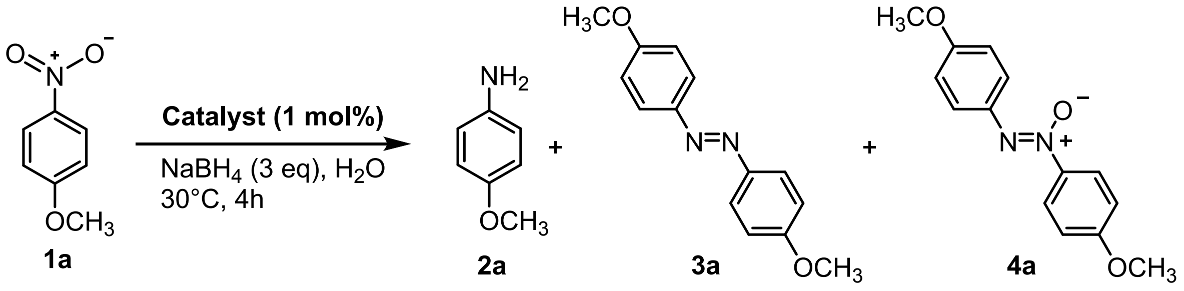

Entry[a]	Catalyst	Ni loading w/w%	Conv %[b]	2a:3a:4a[b]
1	**Ni@ZrPGly-1**	12.15	97	100:0:0
2	**Ni@ZrPGly-2**	8.22	88	100:0:0
3	**Ni@ZrPGly-3**	4.63	27	100:0:0
4	**Ni@ZrP**	7.57	0	-

[a] Reaction conditions: **1a** (0.2 mmol), Catalyst (1 mol%), $NaBH_4$ (3 eq.), H_2O (2 mL) at 30°C for 4h. [b] Conversion and product ratios determined by GLC analyses.

Table S3. Comparison of catalytic activity of **Ni@ZrPGly-1** with other Ni-catalysts in the hydrogenation of nitroarenes.

Catalyst	(Ni mol%)	Reducing agent	Medium	Temp (°C)	Time (h)	Yield %	Ref.
Ni@ZrPGly-1	3	$NaBH_4$ (3 eq)	MeOH	30	1-6	80-98	This work
Ni–NiO@bTiO$_2$	3.5	H_2 (20 bar)	iPrOH	130	6	73-99	[a]
Ni-NHC-olefin	3	H_2 (10 bar)	H_2O (SDS)	80	13	72-99	[b]
Ni/NiFe$_2$O$_4$	70mg/1 mmol	H_2 (0.5 MPa)	H_2O	70	3	82.3-90	[c]
Ni/SiC-B$_{0.5}$	4	H_2 (1 MPa)	EtOH	90	1	70.1-95.5	[d]
Ni@β-CD@Fe$_3$O$_4$NPs	1.5	$NaBH_4$ (4 eq)	H_2O	25	1-4	83-99	[e]
Ni@N–C	6	$NaBH_4$ (4 eq)	MeOH	25	5 min	45-99	[f]
$NiCl_2 \cdot 6H_2O$/dppe	2.5	NH_3BH_3 (3 eq)	EtOH	100	12	72-97	[g]
Ni/meso-SiO$_2$	8.1	H_2 (2 bar)	H_2O/iPrOH	25	24	78-98	[h]
Ni/C	14.3	H_2 (5 bar)	H_2O/MeOH	25	5.5-8.5	92-99	[i]
Raney Ni	14-28 wt%	H_2 (3 bar)	H_2O	25	24-28h	90-98	[k]

[a] Mishra, J., Mrugesh, P., Subramanian, P. S., Pratihar, S. *Catal. Sci. Technol.*, **2025**,*15*, 7173-7189

[b] Avello, M. G., Martínez, J. B., Romero, T., Papaefthimiou, V., Chetcuti, M. J., Ritleng, V., Pham-Huu, C., Oelschlaeger, C., Michon, C. *ACS Sust. Chem. Eng.* **2024**, *12*, 10739-10751.

[c] More, G. S., Kharb, S., Gill, P., Srivastava, R. *Appl. Catal. A: Gen.* **2024**, *681*, 119785.

[d] Zhao, J. X. *Appl. Catal. A: Gen.* **2024**, *678*, 119726

[e] Paymifar, S, Foroozandeh, A, Abdouss, M., Marjani, A. P. *Sci. Rep.* **2024**, *14*, 28493

[f] Cao, L., Abbas, S. A., Jeong, S. H., Seo, D., Nam, K. M., Park, J. K. *Adv. Synth. Catal.* **2023**, *365*, 2230–2239.

[g] Dewangan, C., Kumawat, S., Bhatt, T., Natte, K. *ChemComm*. **2023**, *59*, 14709–14712.

[h] Hu, Y., Liu, M., Bartling, S., Lund, H., Atia, H., Dyson, P. J., Beller, M., Jagadeesh, R.V. *Sci. Adv.* **2023**, *9*,eadj8225.

[i] Tang, Q., Yuan, Z., Jin, S., Yao, K., Yang, H., Chi, Q., Liu, B. *React. Chem. Eng.* **2020**, *5*, 58–65.

[k] Ding, P.; Fayad, E; Abu Ali, O. A.; Hua-Li Qin, H.-L. *Tetrahedron* **2024**, *167*, 134269.

Table S4. Comparison of catalytic activity of **Ni@ZrPGly-1** with other Ni-catalysts in the hydrogenation of alkenes.

Catalyst	(Ni mol%)	Reducing agent	Medium	Temp (°C)	Time (h)	Yield %	Ref.
Ni@ZrPGly-1	3	$NaBH_4$ (3 eq)	MeOH	30	4-40	98-100	This work
Ni single atom	13	$NaBH_4$ (8 eq)	MeOH	25	20 min	52	[a]
Ni-NHC-olefin	3	H_2 (10 bar)	H_2O (SDS)	30-80	13-62	82-100	[b]
Ni/meso-SiO$_2$	8.1	H_2 (2 bar)	H_2O/iPrOH	25	24	82-99	[c]
$[Ni_2(dpaa)_2(bpy)_2(SO_4)(H_2O)]CH_3OH$	1	$NaBH_4$ (6 eq)	MeOH	40	6	1.4-98	[d]
Ni@C-450	8	H_2 (1 bar)	MeOH	25	8-16	37-99	[e]
Raney Ni	100 wt%	Et_3SiH (2.5 eq)	MeOH	25	1-12	61-98	[f]

[a] Lomhual, K., Phetwarotai, W., Phusunti, N. *J. Energy Inst.* **2025**, *121*, 102132.

[b] Avello, M. G., Martínez, J. B., Romero, T., Papaefthimiou, V., Chetcuti, M. J., Ritleng, V., Pham-Huu, C., Oelschlaeger, C., Michon, C. *ACS Sust. Chem. Eng.* **2024**, *12*, 10739-10751.

[c] Hu, Y., Liu, M., Bartling, S., Lund, H., Atia, H., Dyson, P. J., Beller, M., Jagadeesh, R.V. *Sci. Adv.* **2023**, *9*, eadj8225.

[d] Kani, I., Unver, H. *Polyhedron* **2020**, *187*, 114649

[e] Gao, J.; Ma, R.; Feng, L.; Liu, Y.; Jackstell, R.; Jagadeesh, R.V.; Beller, M. *Angew. Chem. Int. Ed.* **2021**, *60*, 18591–18598

[f] Liua, C.-H.; Xua, M.; Luoa, Q.; Wanga, Z.; Tana, W.; Zhaoa, X.; Jia, X. *Tetrahedron* **2024**, *160*, 134040.

Characterization data for all prepared compounds

4-methoxyaniline (2a) [1-3]

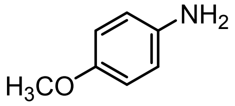

^{1}H-NMR ($CDCl_3$, 400 MHz) δ: 3.21 (s broad, 2H), 3.74 (s, 3H), 6.64 (d, 2H, J = 8.8 Hz), 6.75 (d, 2H, J = 8.8 Hz).
GC-MS (m/z): 123 (70), 109 (7), 108 (100), 80 (53), 65 (5), 53 (15).

Aniline (2b) [1-5]

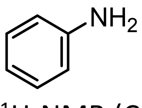

^{1}H-NMR ($CDCl_3$, 400 MHz) δ: 3.47 (s broad, 2H), 6.70 (m, 2H), 6.77 (m, 1H), 7.16 (m 1H).
GC-MS (m/z): 93 (100), 77 (7), 66 (35), 65 (21), 61 (10), 51 (10).

4-chloroaniline (2c) [1-5]

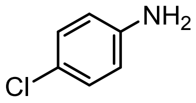

^{1}H-NMR ($CDCl_3$, 400 MHz) δ: 3.99 (s broad, 2H), 6.61 (d, 2H, J = 8.8 Hz), 7.10 (d, 2H, J = 8.8 Hz).
GC-MS (m/z): 129 (33), 127 (100), 102 (2), 100 (9), 92 (13), 65 (12), 63 (5).

4-bromoaniline (2d) [1-5]

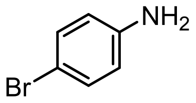

^{1}H-NMR ($CDCl_3$, 200 MHz) δ: 3.52 (s broad, 2H), 6.56 (d, 2H, J = 8. 8 Hz), 7.24 (d, 2H, J = 8.8 Hz).
GC-MS (m/z): 173 (100), 171 (100), 145 (11), 143 (11), 119 (8), 117 (8), 92 (100), 91 (42), 66 (31), 65 (100), 64 (44%), 63 (70), 62 (36), 61 (23), 52 (29), 51 (13), 50 (23).

3-bromoaniline (2e) [5]

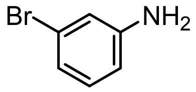

^{1}H-NMR ($CDCl_3$, 200 MHz) δ: 3.70 (s broad, 2H), 6.59 (dd, 1H, J = 8.0, 1.5 Hz), 6.83 (t, 1H, J = 2.0 Hz), 6.87 (d broad, 1H, J = 7.9 Hz), 7.00 (t, 1H, J = 8 Hz).
GC-MS (m/z): 173 (100), 171 (100), 145 (3), 143 (3%), 92 (84), 65 (85), 52 (11).

2-bromoaniline (2f) [5]

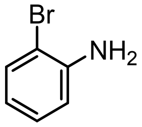

^{1}H-NMR ($CDCl_3$, 200 MHz) δ: 4.07 (s broad, 2H), 6.62 (td, 1H, J = 8.6, 1.3 Hz), 6,76 (dd, 1H, J = 8.0, 1.3 Hz), 7.10 (td, 1H, J = 7.6, 1.2 Hz), 7.39 (dd, 1H, J = 8.0, 1.0 Hz).
GC-MS (m/z): 173 (100), 171 (100), 145 (2), 143 (2), 92 (66), 65 (74), 52 (14).

3-iodoaniline (2g) [6,7]

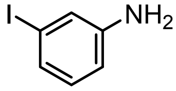

^{1}H-NMR ($CDCl_3$, 200 MHz) δ: 3.65 (s broad, 2H), 6.62 (dd, 1H, J = 8.0, 1.6 Hz), 6,86 (t, 1H, J = 7.9 Hz), 7.02-7.10 (m, 2H).
GC-MS (m/z): 219 (100), 127 (100), 92 (47), 65 (47), 52 (8).

4-methylaniline (2h) [2-5]

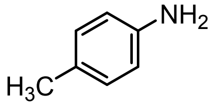

^{1}H-NMR ($CDCl_3$, 400 MHz) δ: 2.24 (s, 3H), 3.30 (s broad, 2H), 6.62 (d, 2H, J = 7.9 Hz), 6.97 (d, 2H, J = 7.9 Hz).
GC-MS (m/z): 107 (84), 106 (100), 89 (8), 79 (15), 77 (19), 63 (6), 52 (9), 51 (10).

3-ethylaniline (2i) [8,9]

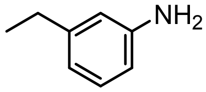

^{1}H-NMR ($CDCl_3$, 400 MHz) δ: 1.23 (t, 3H, J 7.6 Hz), 2.57 (q, 2H, J = 7.6 Hz), 3.61 (s broad, 2H), 6.53 (d, 1H, J = 7.9), 6.55 (s, 1H), 6.63 (d, 1H, J = 7.5 Hz), 7.09 (t, 1H, J = 7.7 Hz).
GC-MS (m/z): 121 (100), 106 (100), 93 (12), 91 (47), 77 (60), 65 (25), 51 (15%).

1-(3-(2-(3-ethylphenyl)hydrazino)phenyl)ethanol (3i)

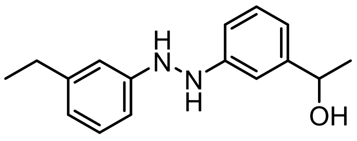

^{1}H-NMR ($CDCl_3$, 400 MHz) δ: 1.17 (t, 3H, J= 7.6 Hz), 1.48 (d, 3H, J= 6.7 Hz), 1.56 (s broad, 1H), 2.52 (q, 2H, 7.6 Hz), 3.66 (s broad, 2H), 4.38 (q, 1H, J =6.7 Hz), 6.33 (d, 1H, J =8.0 Hz), 6.40 (s broad, 1H), 6.51 (d, 1H, J =7.4 Hz), 6.55 (d, 1H, J =7.7 Hz), 6.72 (s, 1H), 6.77 (d, 1H, J =7.5 Hz), 7.01 (t, 1H, J= 7.8 Hz), 7.11 (t, 1H, J= 7.7 Hz).

3-(1-idroxyethyl)aniline (2j) [1,2,10]

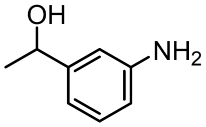

^{1}H-NMR ($CDCl_3$, 200 MHz) δ: 1.47 (d, 3H, J = 6.4 Hz), 2.73 (s broad, 2H), 4.81 (q, 1H, J = 6.4 Hz), 6.57-6.63 (m, 1H), 6.72-6.77 (m, 2H), 7.14 (m, 1H).
GC-MS (m/z): 137 (44), 122 (23), 94 (100), 93 (17), 91 (6), 77 (26), 65 (15), 51 (5).

4-(metoxycarbonyl)aniline (2k) [1,2,4]

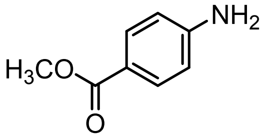

^{1}H-NMR ($CDCl_3$, 200 MHz) δ: 3.85 (s, 3H), 4.06 (s broad, 2H), 6.63 (d, 2H, J = 8.2 Hz)), 7.85 (d, 2H, J = 8.2 Hz).
GC-MS (m/z): 151 (100), 121 (50), 120 (100), 108 (9), 93 (2), 92 (100), 66 (210, 65 (100%), 63 (37), 52 (18), 51 (6).

(4-aminophenyl)methanol (2l) [1,11]

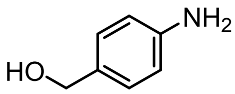

1H-NMR ($CDCl_3$, 400 MHz) δ: 1.64 (s broad, 1H), 3.67 (s broad, 2H), 4.55 (s ,2H), 6.67 (d, 2H, J = 8.2 Hz), 7.16 (d, 2H, J = 8.2 Hz)
GC-MS (m/z):123 (98), 122 (73), 106 (100), 94 (83), 77 (57), 65 (27), 51 (15)

(3-aminophenyl)methanol (2m) [1,11]

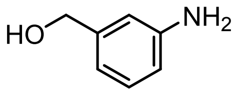

^{1}H-NMR ($CDCl_3$, 400 MHz) δ: 1.72 (s broad, 1H), 3.68 (s broad, 2H), 4.60 (s, 2H), 6.61 (dd, J =7.9, 1.7 Hz, 1H), 6.70 (s, 1H), 6.74 (d, 1H, J =7.6 Hz), 7.14 (t, 1H, J =7.7 Hz)
GC-MS (m/z): 123 (92), 122 (14), 106 (22), 94 (100), 77(47), 65 (22), 51 (10)

6-aminoquinoline (2n) [2,5]

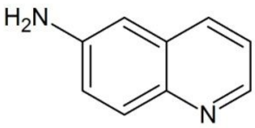

^{1}H-NMR ($CDCl_3$, 200 MHz) δ: 3,92 (s broad, 2H), 6.89 (d, 1H, J = 2.6 Hz), 7.15 (dd, 1H, J = 9.0, 2.6 Hz), 7.26 (dd, 1H, J = 8.4, 4.2 Hz), 7.89 (dd, 1H, J = 8.4, 1.6 Hz), 7.90 (d, 1H, J = 9.0 Hz), 8.65 (dd, 1H, J = 4.2, 1.6 Hz).
GC-MS (m/z): 145 (100), 144 (100), 127 (8), 118 (53), 117 (100), 116 (100), 91 (27), 90 (100), 89 (100), 72 (46), 63 (75), 52 (32), 51 (21).

Ethylbenzene (6a) [13]
GC-MS (m/z): 106 (52), 91 (100), 78 (15), 77 (17), 65 (19), 51 (21%)

1-Chloro-4-ethylbenzene (6b) [13]
GC-MS (m/z): 142 (12), 140 (37), 127 (33), 125 (100), 105 (52), 89 (22), 77 (23), 63 (18), 51 (19)

4-Ethylphenol (6c) [13]
GC-MS (m/z): 122 (36), 107 (100), 91 (6), 77 (22), 65 (6), 51 (6)

4,4'-dimethoxybibenzyl (6d) [13,14]
^{1}H-NMR ($CDCl_3$, 400 MHz) δ: 2.83 (s, 4H), 3.79 (s, 6H), 6.82 (d, 1H, J =8.4 Hz), 7.08 (d, 1H, J =8.4 Hz)
GC-MS (m/z): 242 (13), 121 (100), 106 (3), 91 (8), 78 (15), 77 (13), 65 (3), 51 (3)

Methyl 3-phenylpropanoate (6e) [13]
GC-MS (m/z): 164 (25), 104 (100), 91 (69), 79 (27), 78 (39), 77 (42), 65 (18), 59 (17), 51 (19)

Cyclohexylbenzene (6f) [13]
GC-MS (m/z): 160 (91), 131 (12), 117 (84), 115 (39), 104 (100), 91 (52), 78 (14), 65 (7), 51 (6)

Cyclooctane (6g) [13]
GC-MS (m/z): 112 (69), 97 (11), 84 (31), 83 (59), 70 (57), 69 (58), 56 (100), 55 (97)

Figure S12: 1H-NMR spectrum of **2a**: R = 4-OMe

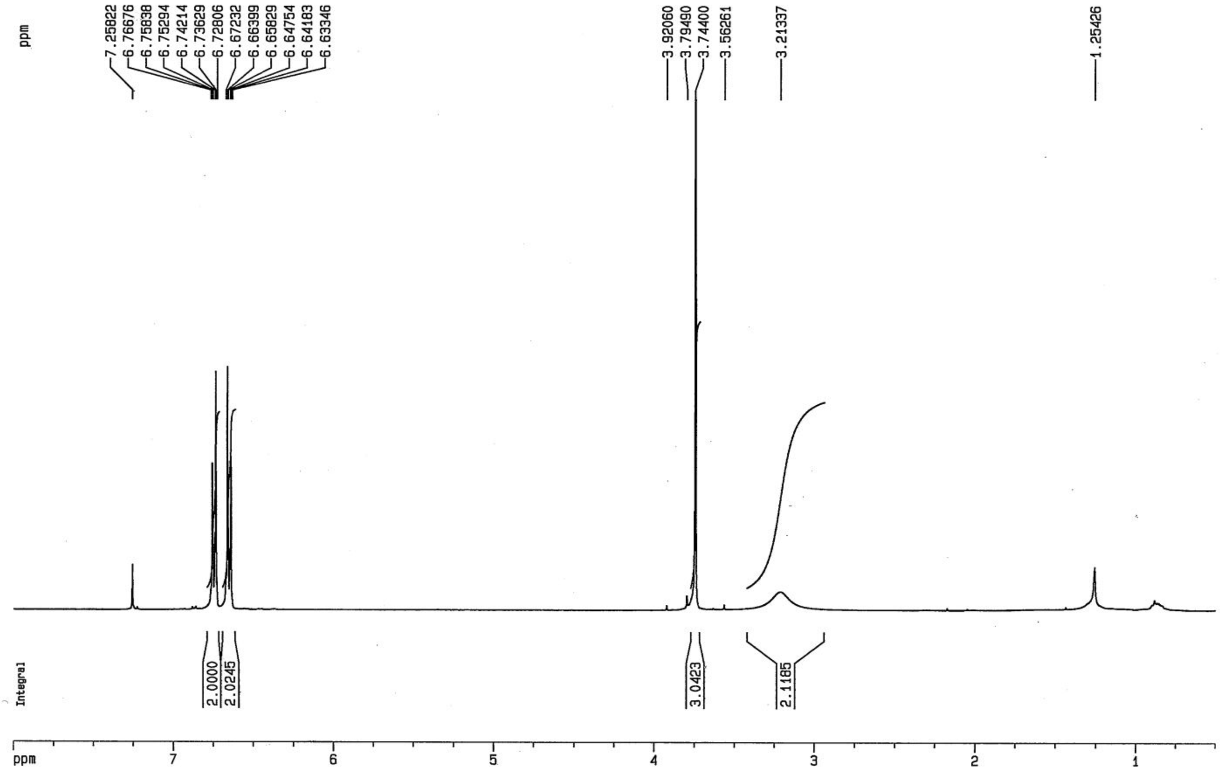

Figure S13: 1H-NMR spectrum of **2b**: R = H

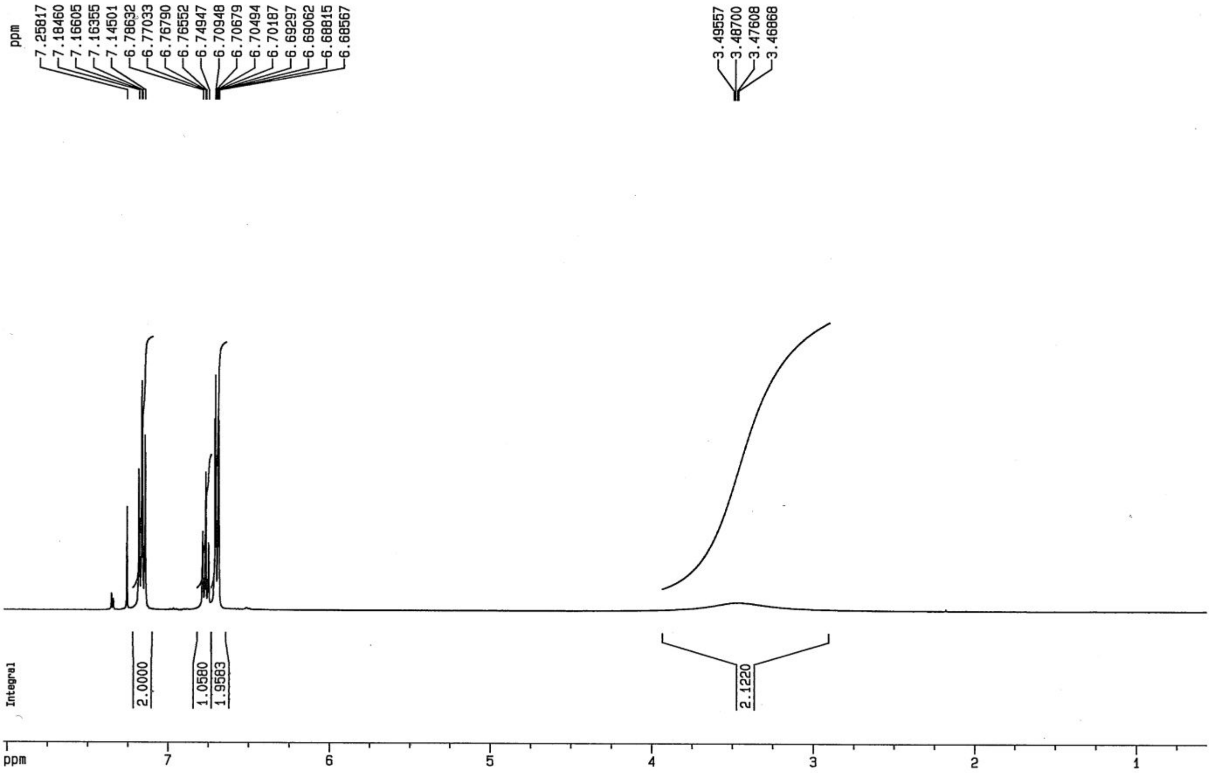

Figure S14: 1H-NMR spectrum of **2c**: R = 4-Cl

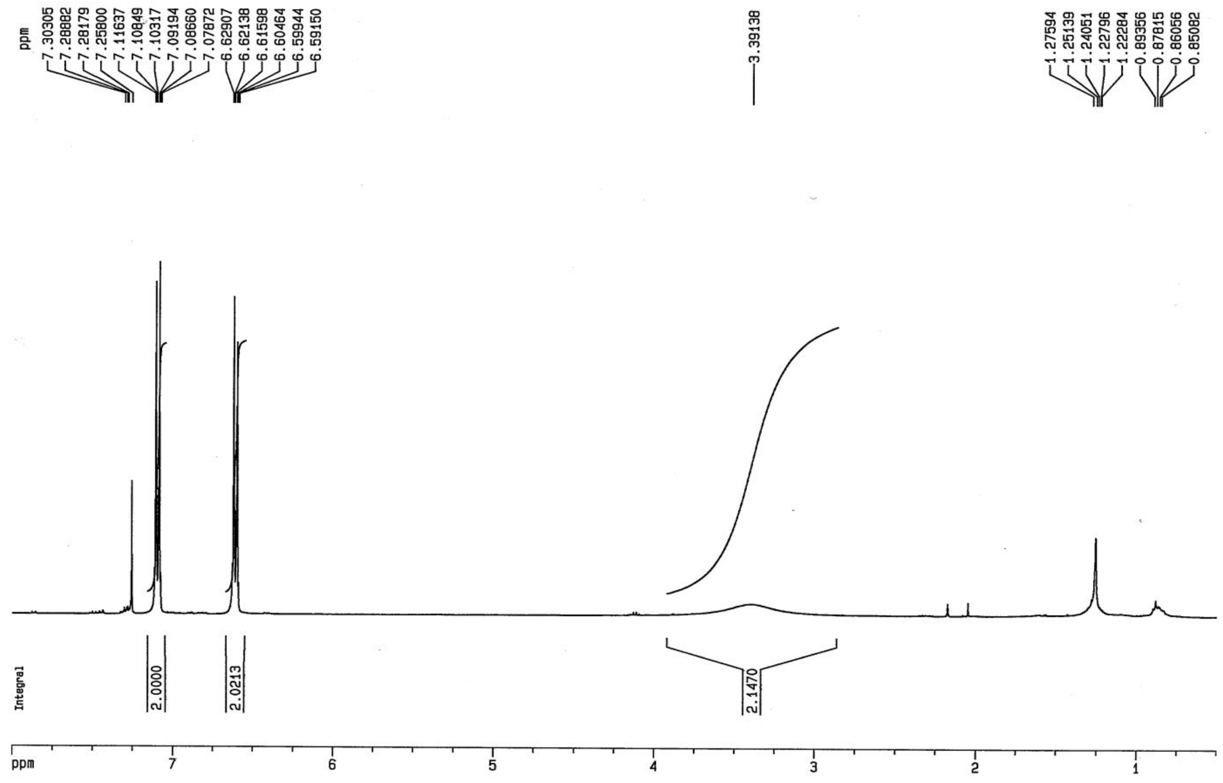

Figure S15: 1H-NMR spectrum of **2d**: R = 4-Br

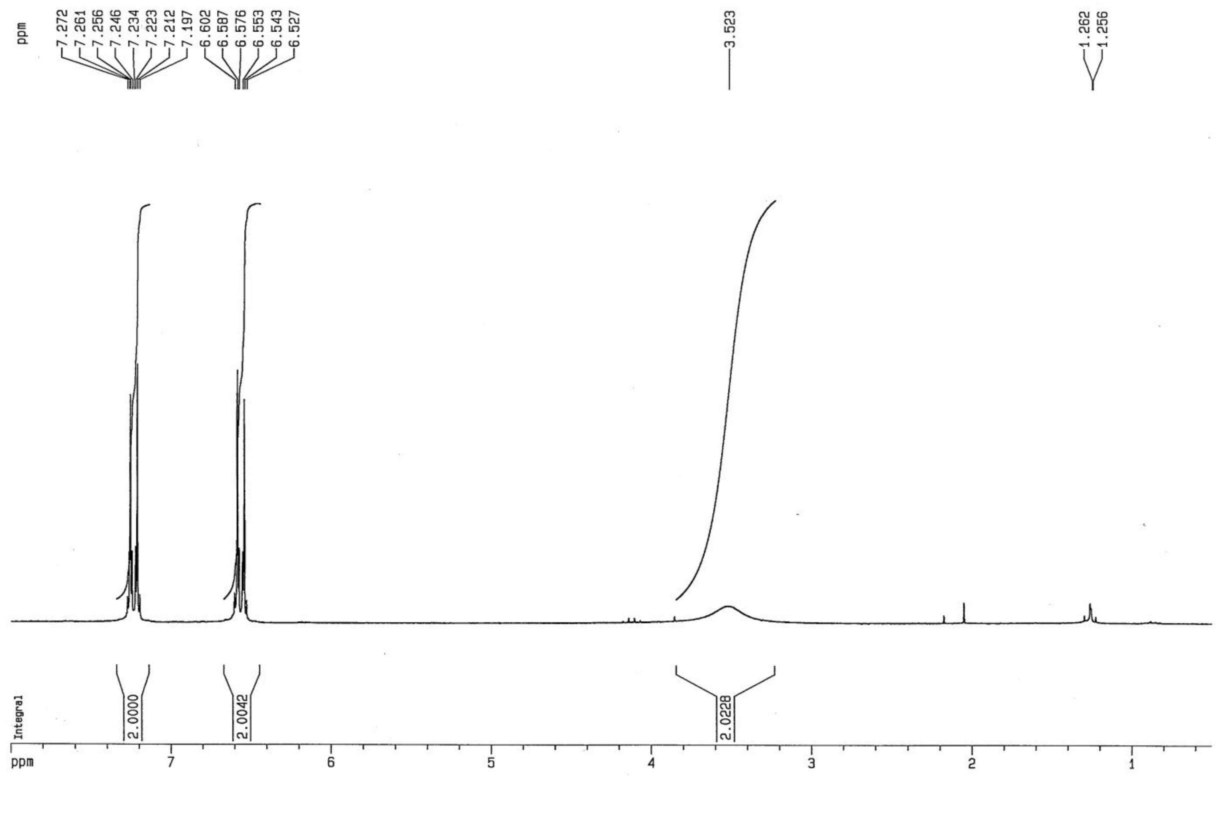

Figure S16: 1H-NMR spectrum of **2e**: R = 3-Br

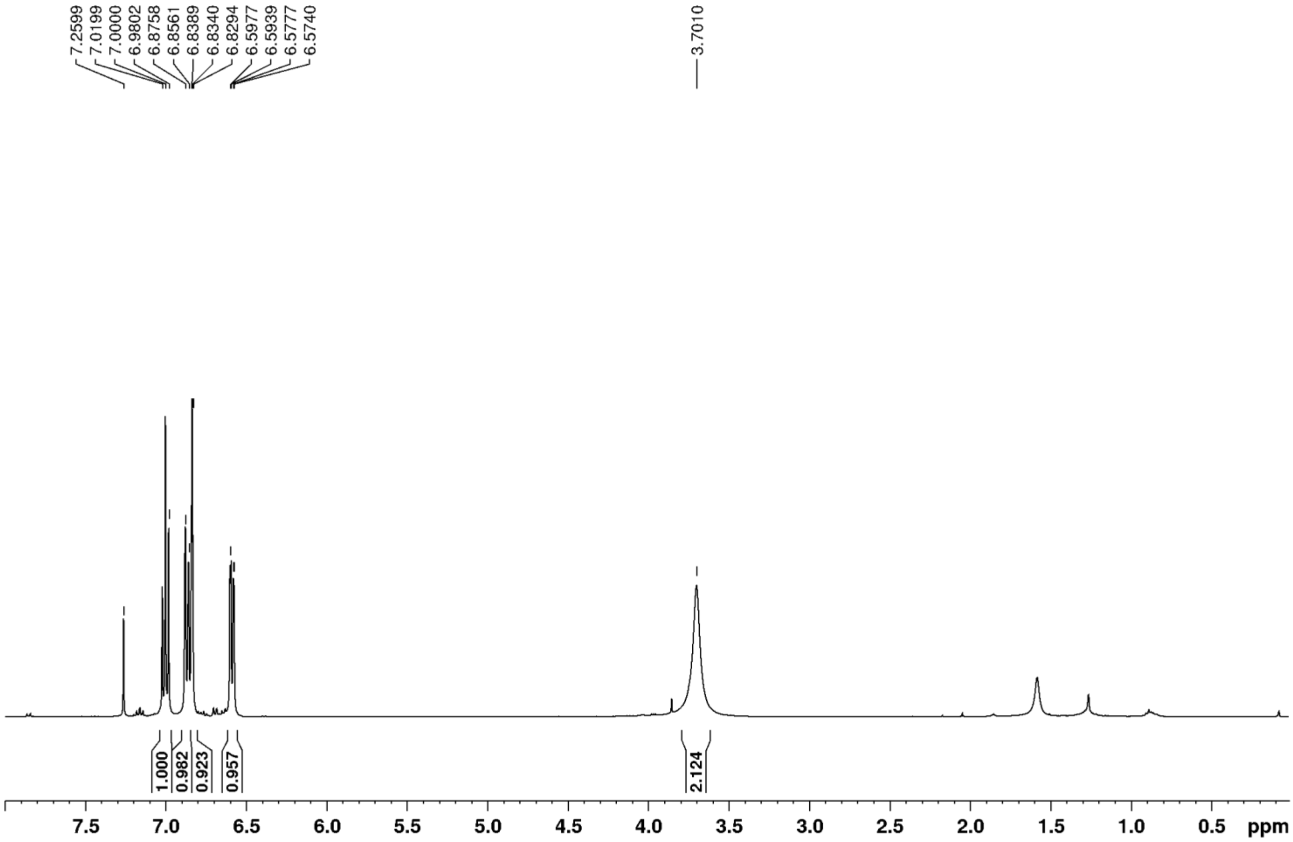

Figure S17: 1H-NMR spectrum of **2f**: R = 2-Br

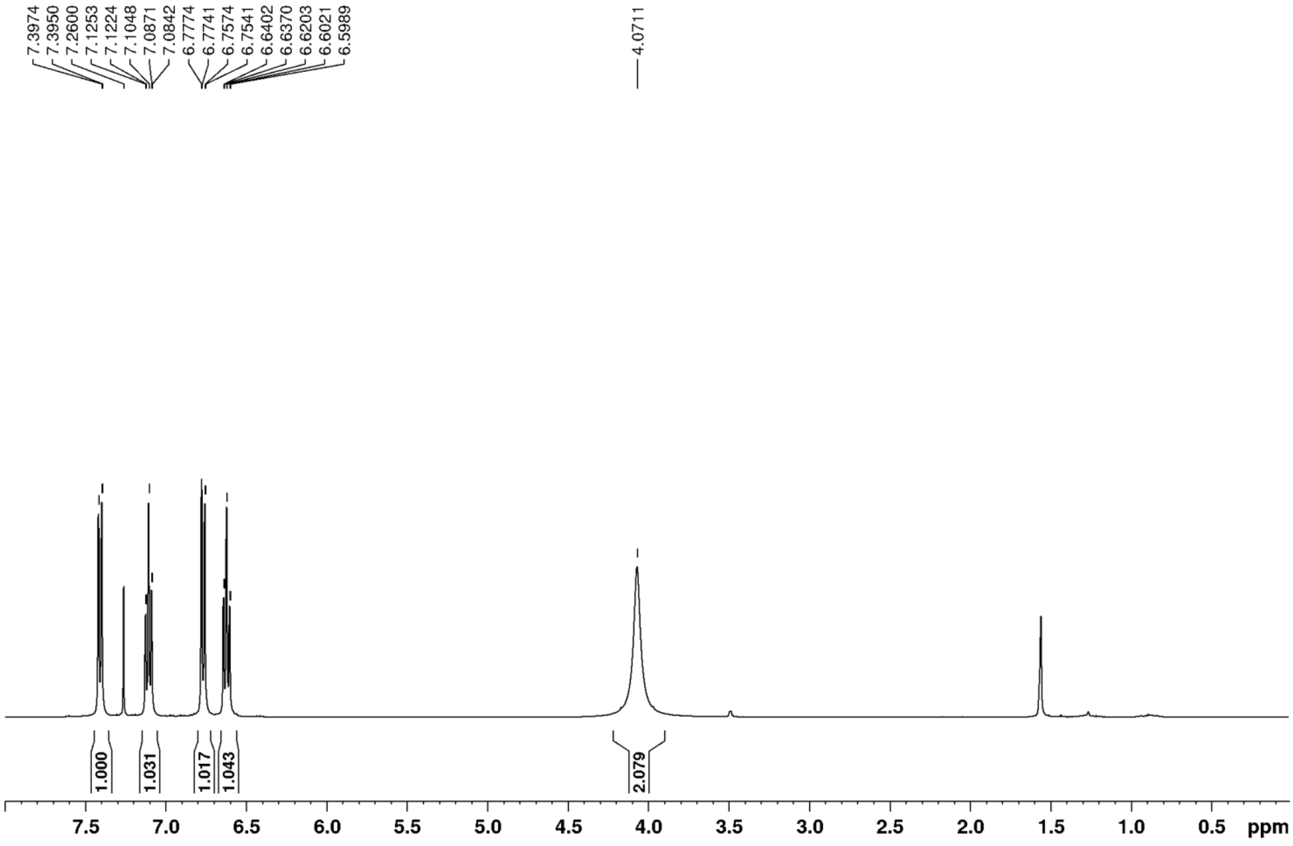

Figure S18: 1H-NMR spectrum of **2g**: R = 3-I

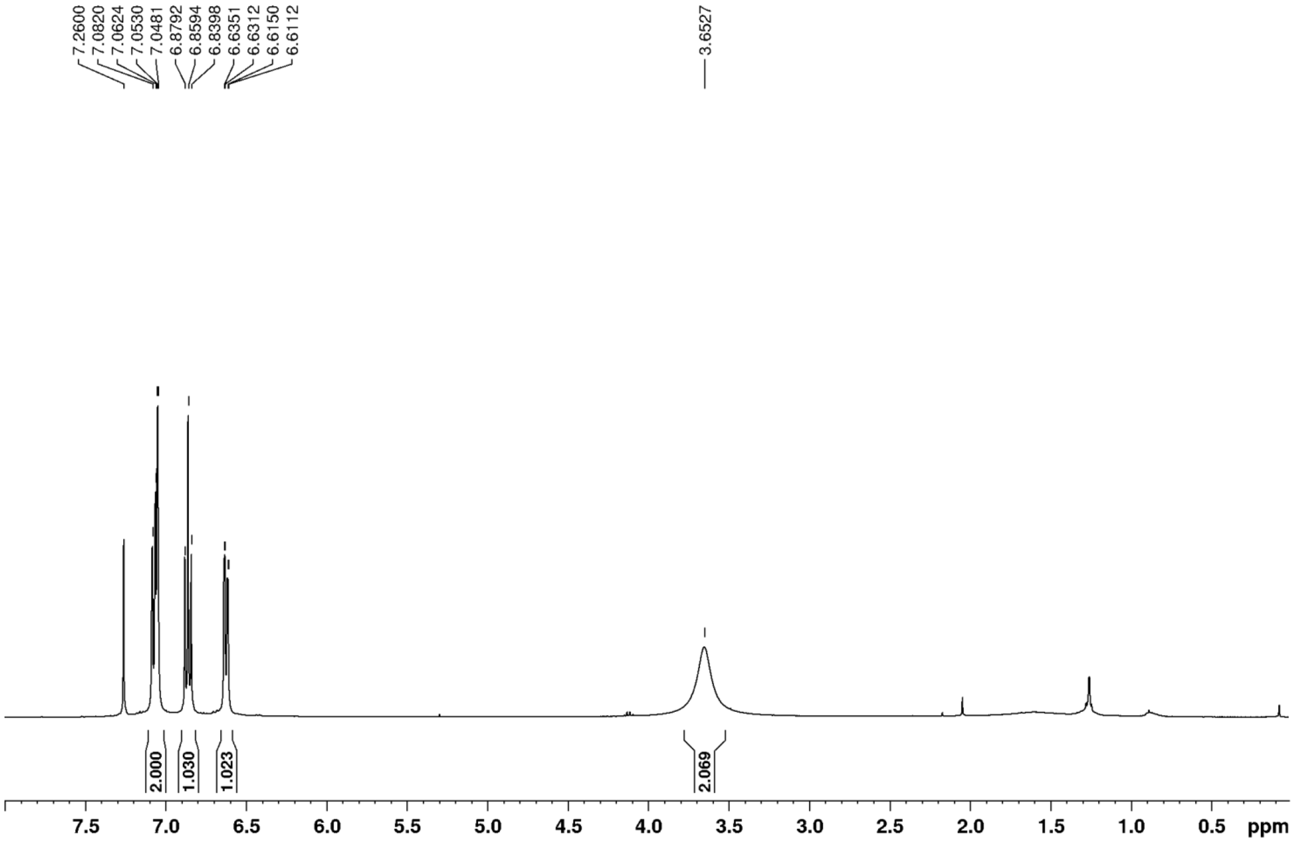

Figure S19: 1H-NMR spectrum of **2h**: 4-Me

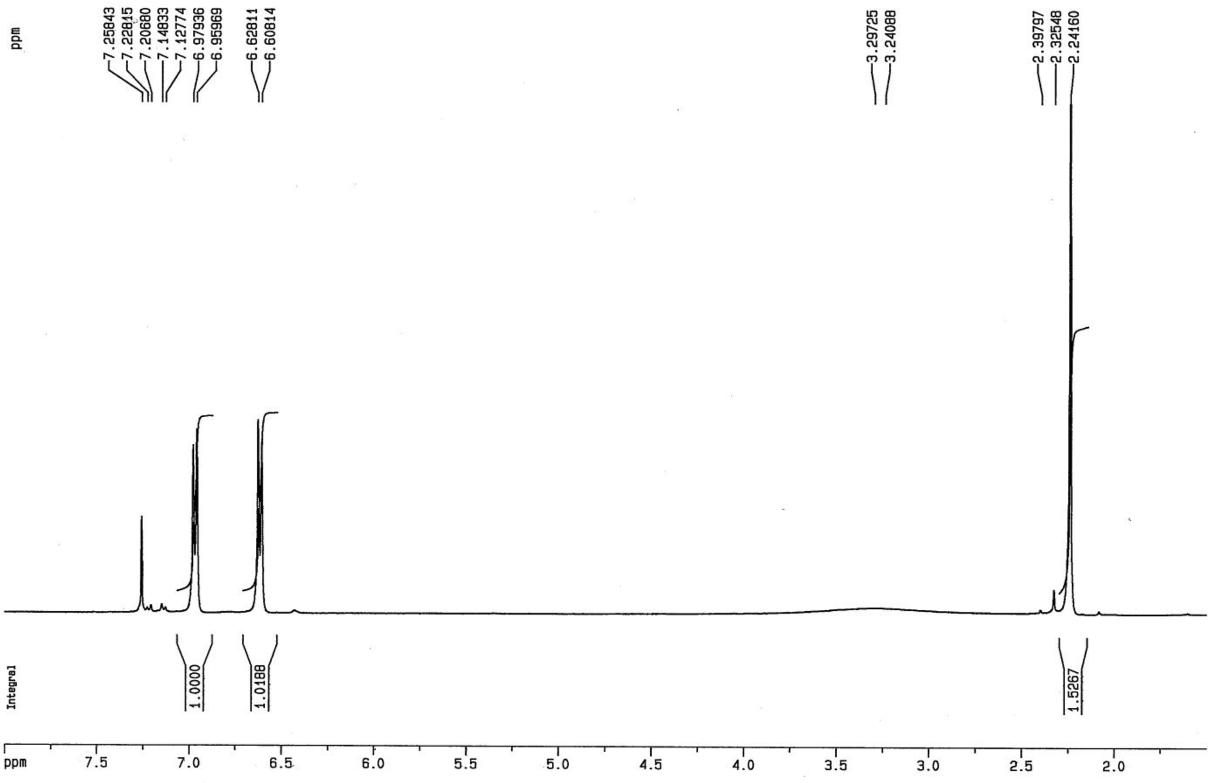

Figure S20: 1H-NMR spectrum of **2i**: R = 3-CH_2CH_3

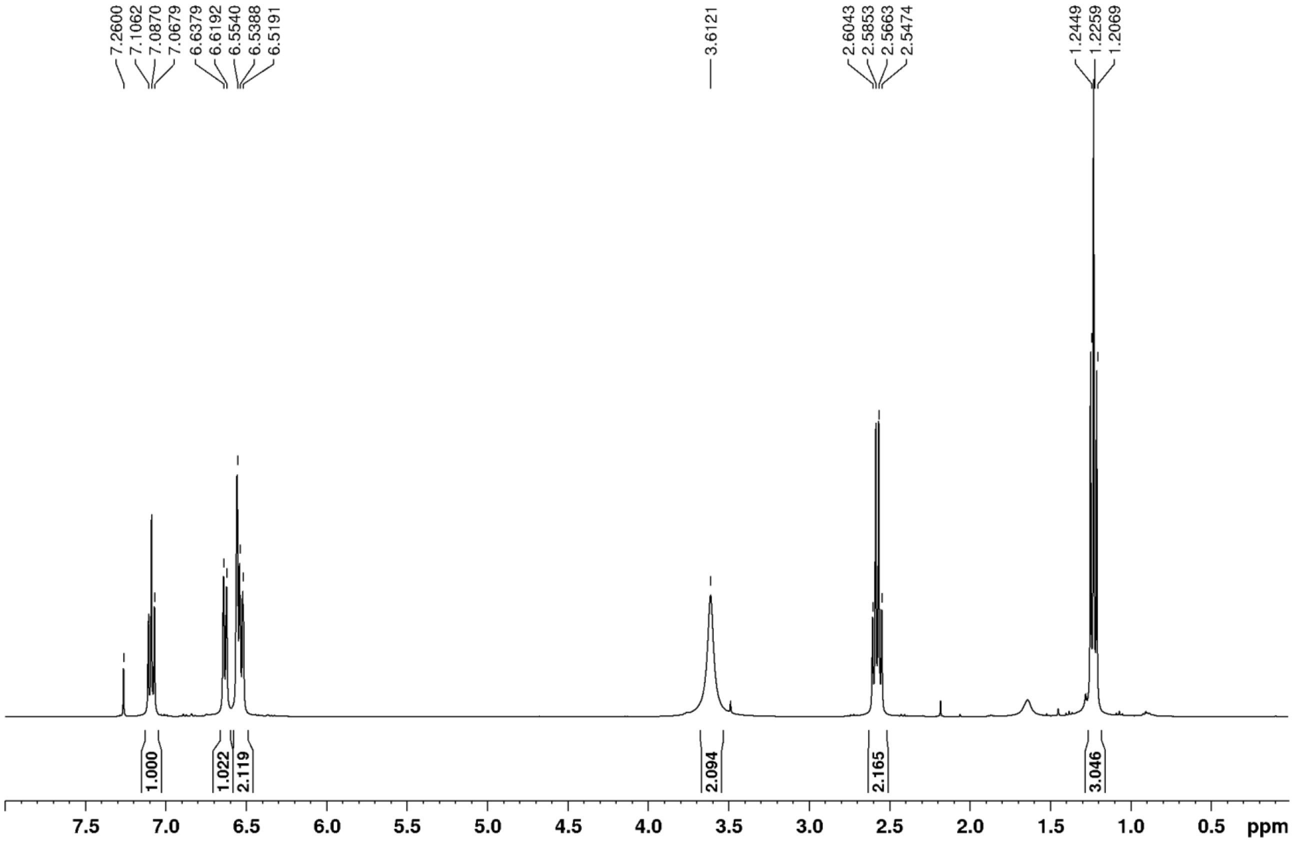

Figure S21: 1H-NMR spectrum of **3i**: diazo-compound

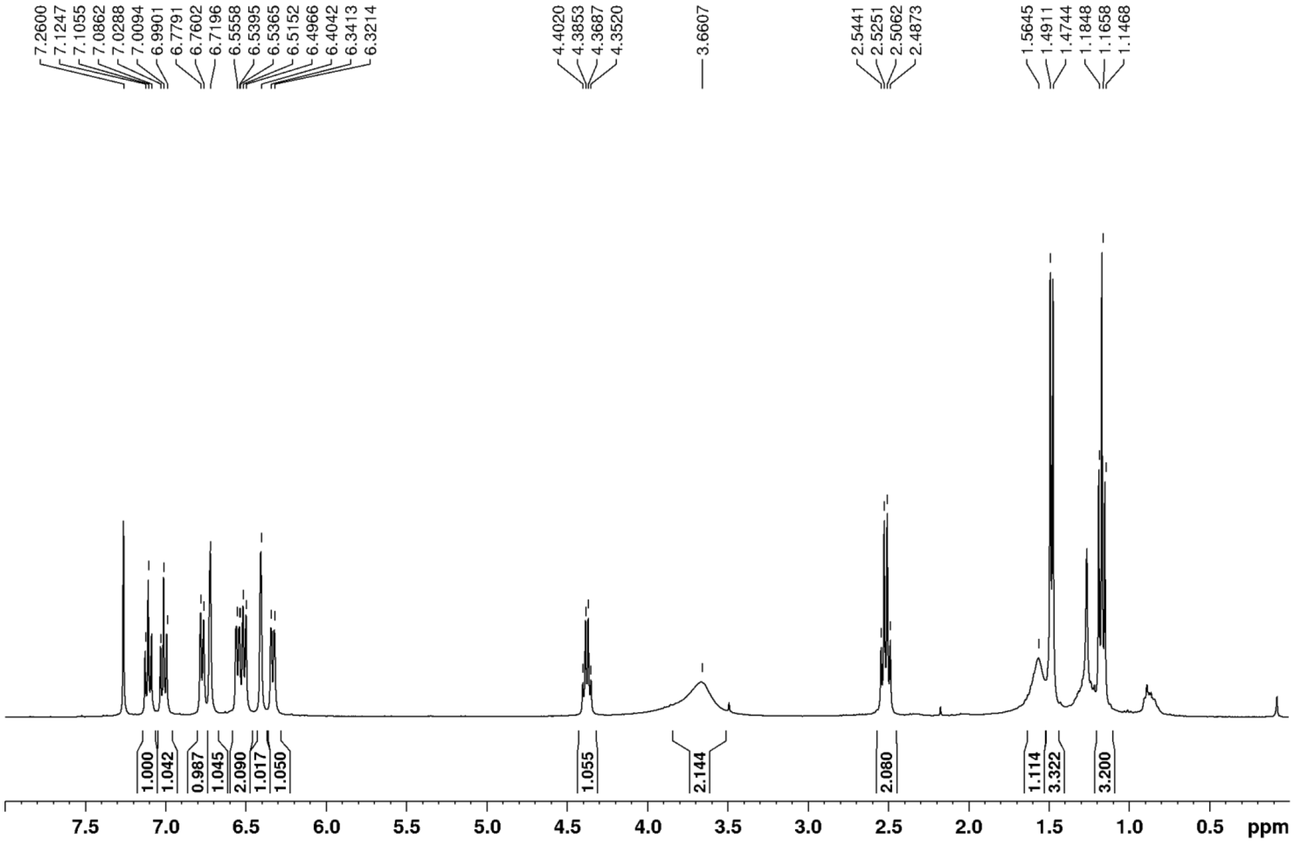

Figure S22: 1H-NMR spectrum of **4j**: R = 3-CH(OH)Me

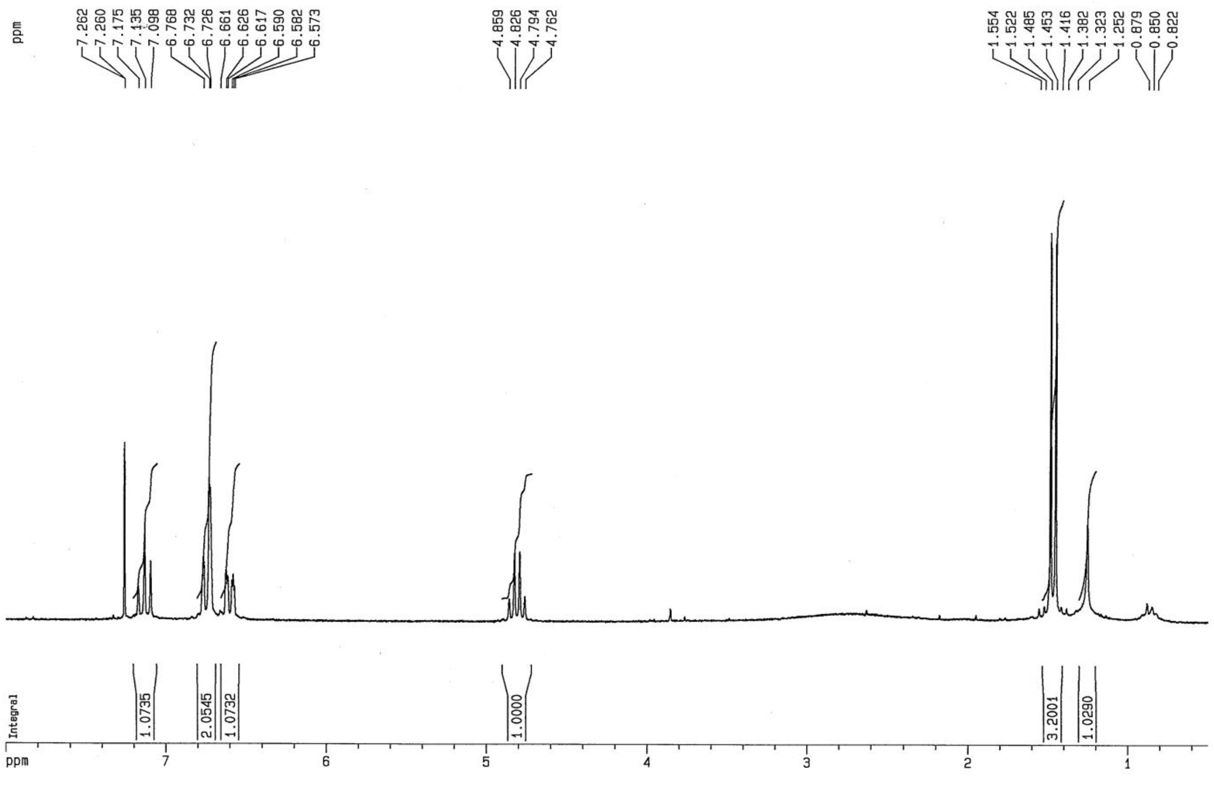

Figure S23: 1H-NMR spectrum of **4k**: R = 4-COOMe

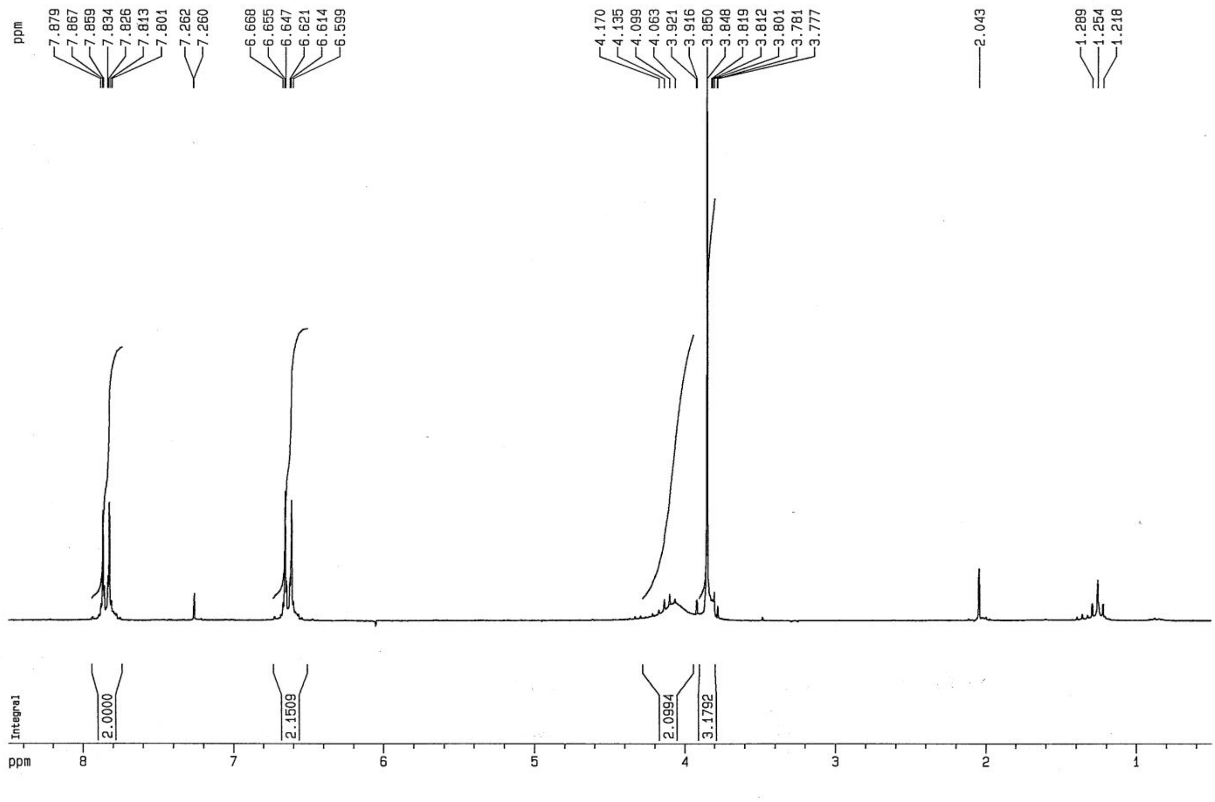

Figure S24: 1H-NMR spectrum of **4l**: R = 4-CH$_2$OH

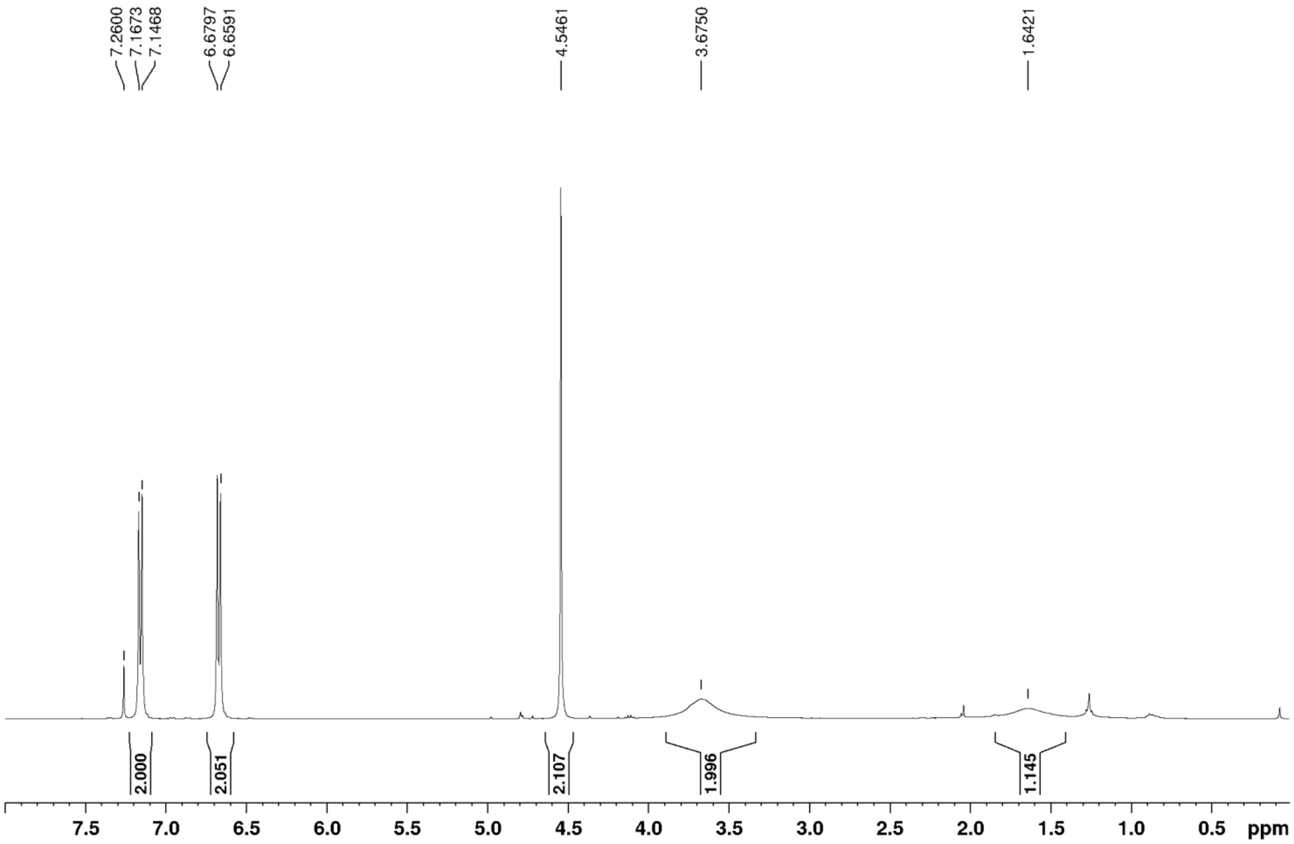

Figure S25: 1H-NMR spectrum of **4m**: R = 3-CH$_2$OH

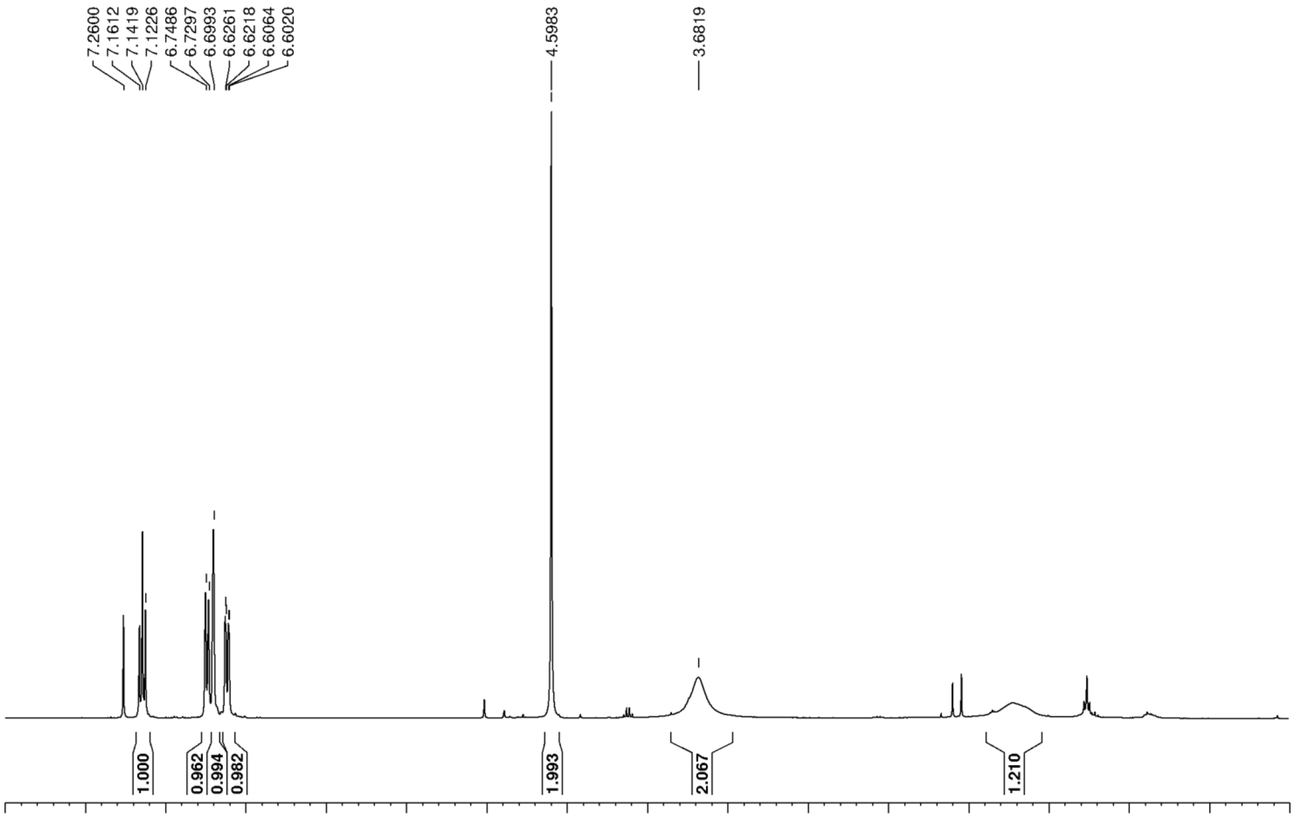

Figure S26: 1H-NMR spectrum of **4n**: 6-nitroquinoline

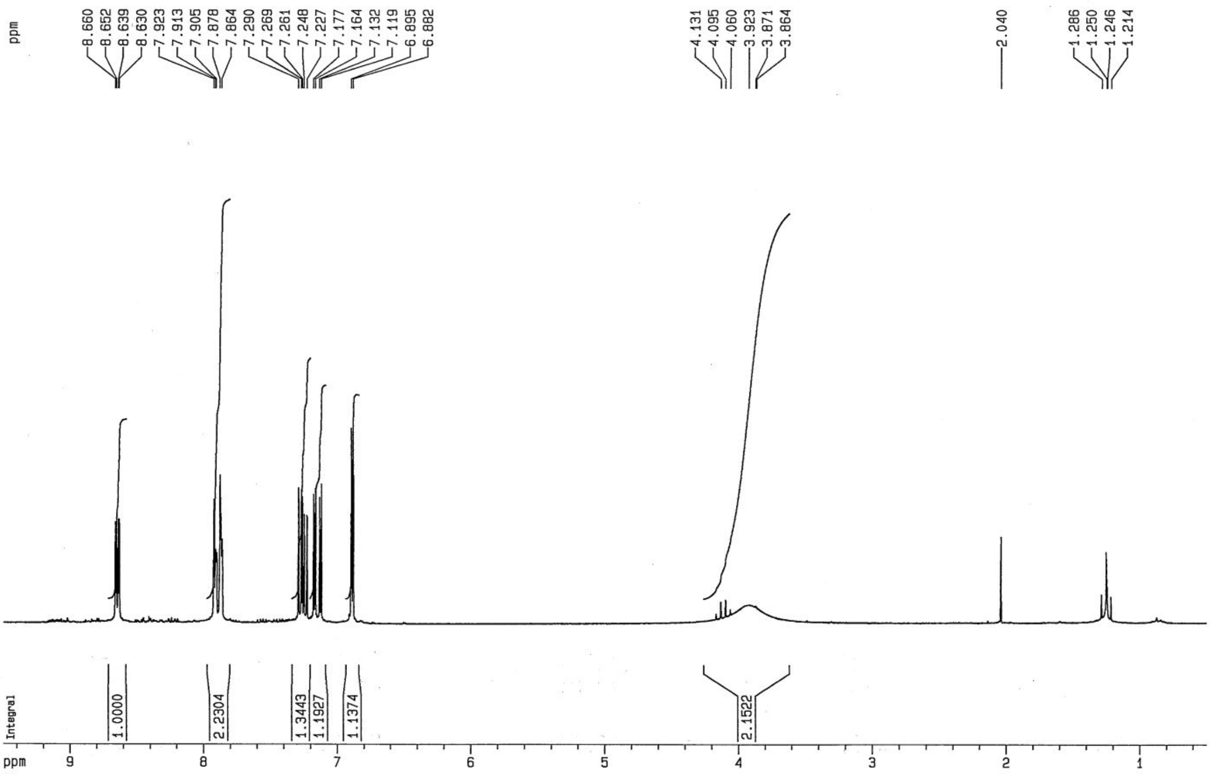

Figure S27: Hydrogenation of styrene (**5a**): MS spectrum of ethylbenzene (**6a**)

Analysis Report

Agilent

Sample Information

**Sample Name**	Ni163C_STIRENE_1	**Data File Path**	D:\MassHunter\GCMS\1\data\GreenSOC2\GreenSOC_627.D
**Sample ID**		**Acq Time (Local)**	10/28/2025 3:32:57 AM (UTC+01:00)
**Instrument**	8890-5977C	**Acq Method Path**	D:\MassHunter\GCMS\1\methods\501103005SD3.M
**MS Type**	Q	**Acq SW Version**	MassHunter GC/MS Acquisition 10.2.530.3 21-Nov-2023 Copyright © 1989-2023 Agilent Technologies, Inc.
**Inj Vol (ul)**	0	**IRM Status**	
**Sample Position**	1	**DA Method Path**	D:\MassHunter\GCMS\1\data\GreenSOC2\GreenSOC_627.D\Results\Qual\Version4\default.m
**Plate Position**		**Target Source Path**	
**Acq Operator**	ORIANA	**Result Summary**	

Sample Chromatograms

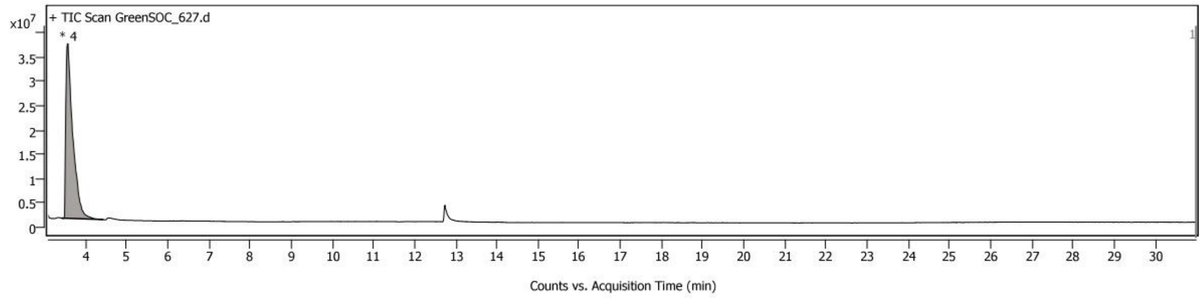

Chromatogram Peaks

Peak	Start	RT	End	Height	Area	Area %	SNR
1	3.411	3.573	4.436	35829798	425879336	100.00	

Sample Spectra

+ Scan (rt: 3.583 min)

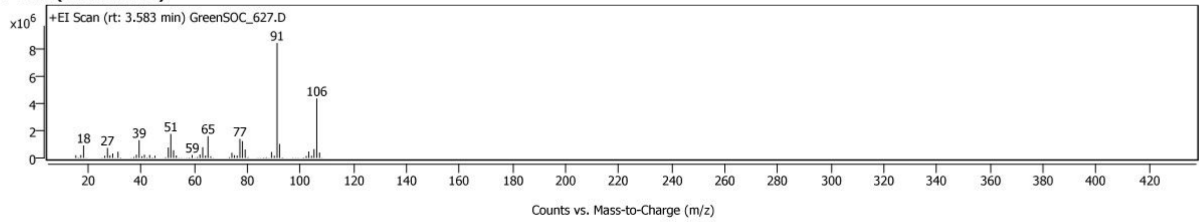

Figure S28: Hydrogenation of 4-chlorostyrene (**5b**): MS spectrum of 1-chloro-4-ethylbenzene (**6b**)

File :C:\msdchem\1\data.6\GREENSOC_947.D
Operator : ORIANA
Acquired : 23 Feb 2024 19:35 using AcqMethod METHODA.M
Instrument : greensoc
Sample Name: NI-178
Misc Info :
Vial Number: 1

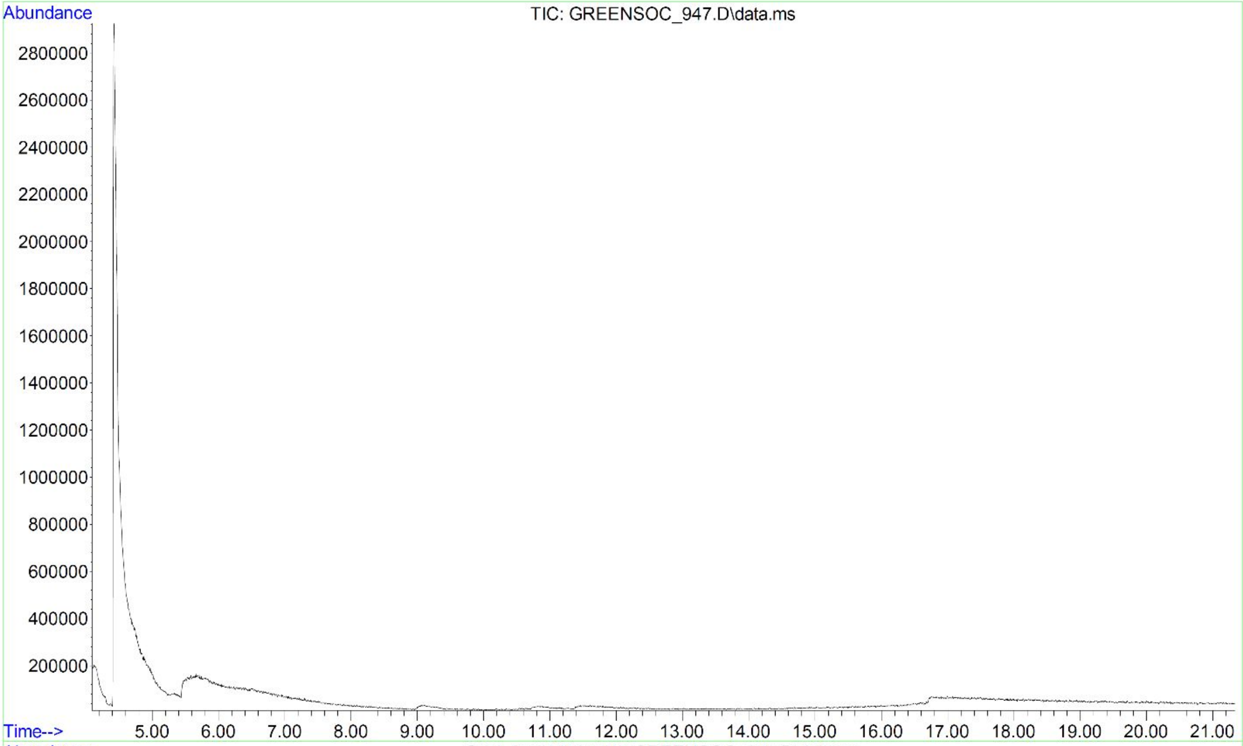

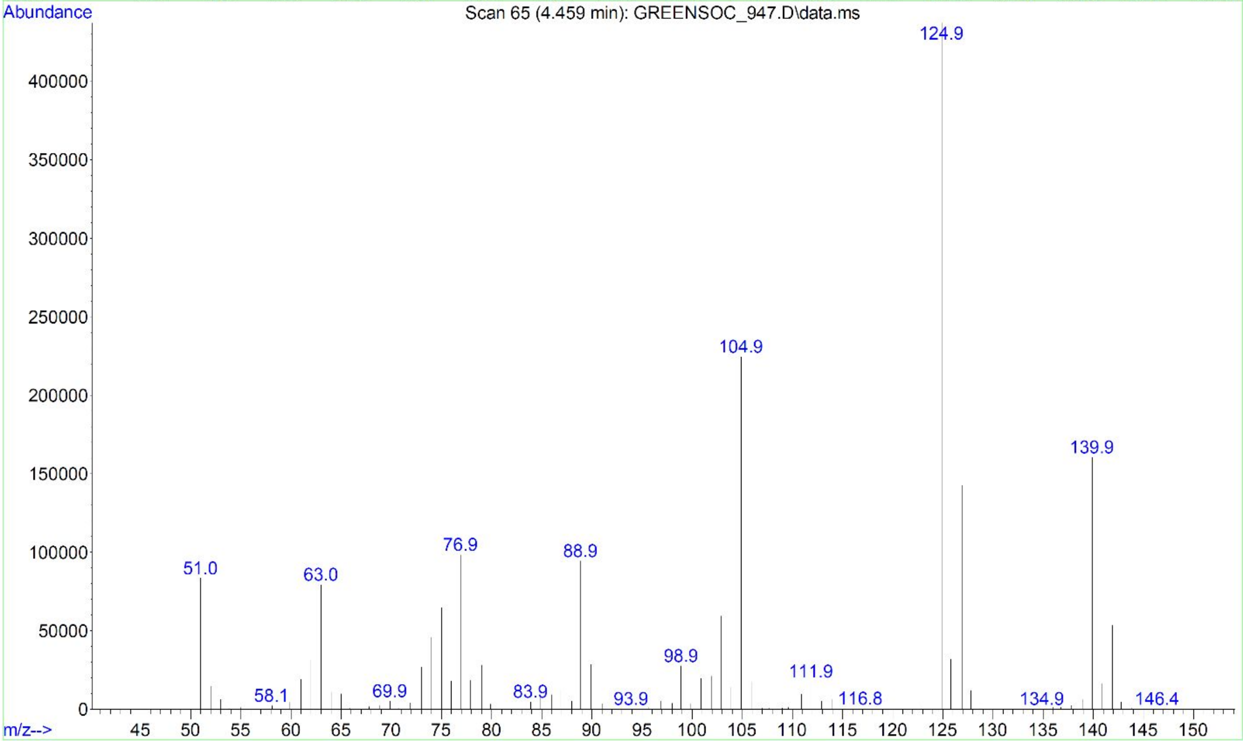

Figure S29: Hydrogenation of 4-Acetoxystyrene (**5c**): MS spectrum of 4-ethylphenol (**6c**)

File :C:\msdchem\1\data.6\GREENSOC_948.D
Operator : ORIANA
Acquired : 23 Feb 2024 20:13 using AcqMethod METHODA.M
Instrument : greensoc
Sample Name: NI-180
Misc Info :
Vial Number: 1

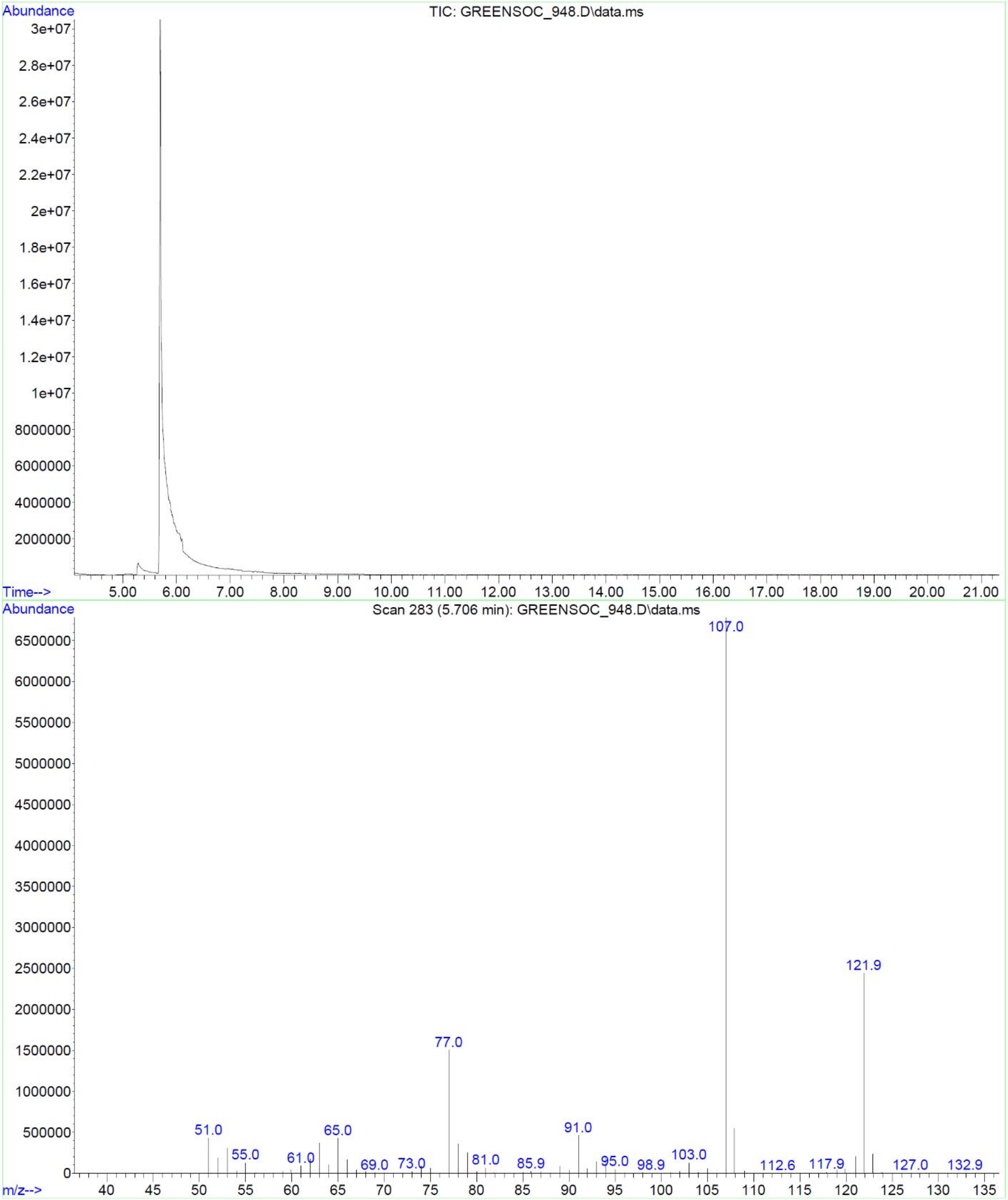

Figure S30: Hydrogenation of 4,4'-Dimethoxystilbene (**5d**): MS spectrum of 4,4'-dimethoxybibenzyl (**6d**)

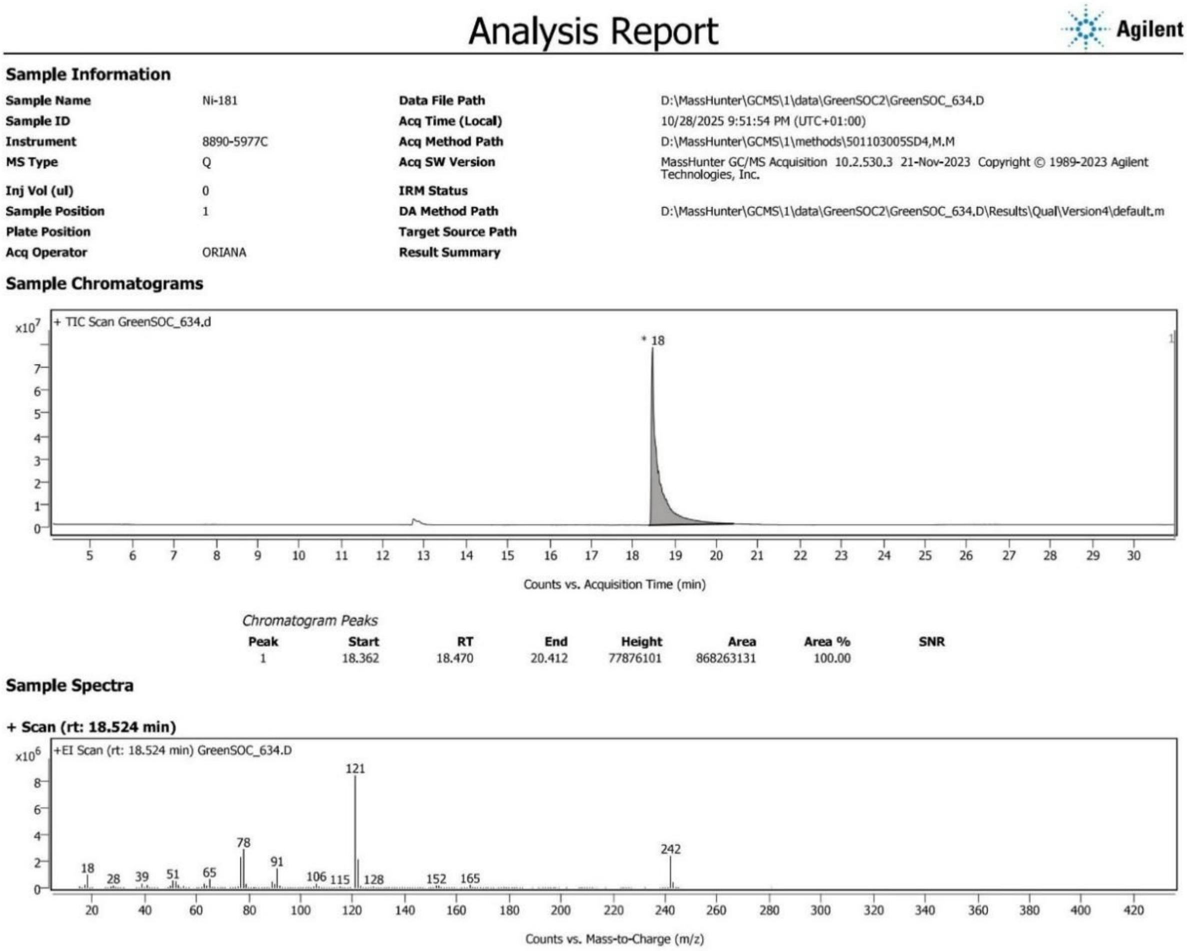

Figure S31: 1H-NMR spectrum of 4,4'-dimethoxybibenzyl (**6d**)

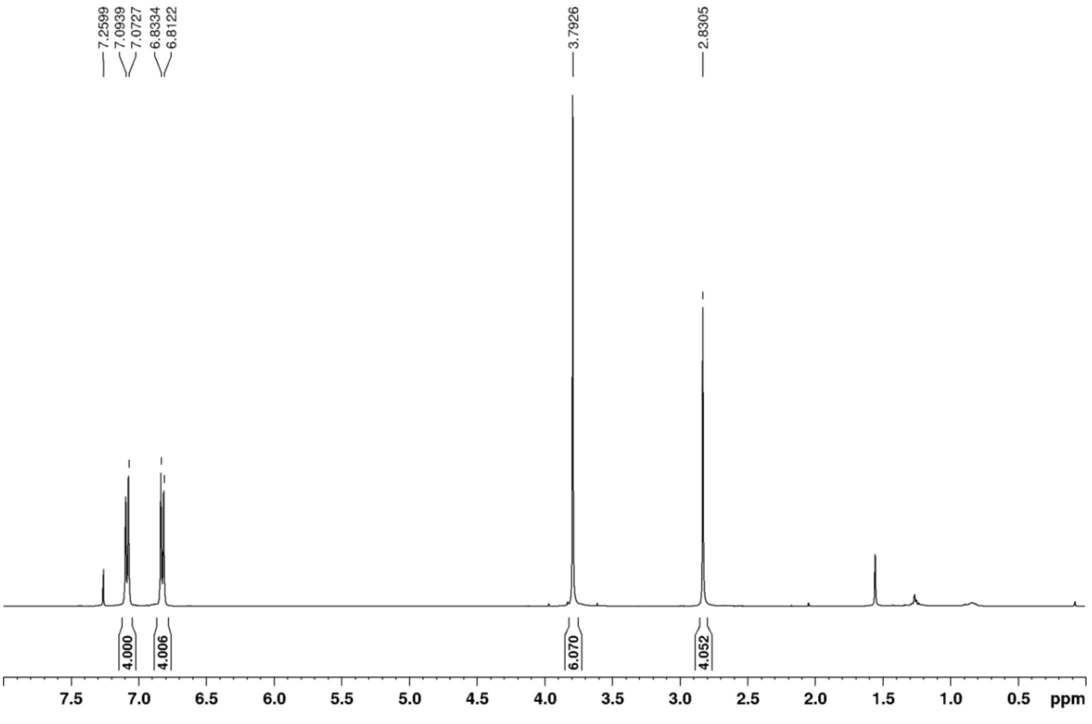

Figure S32: Hydrogenation of methyl 3-phenylacrylate (**5e**): MS spectrum of methyl 3-phenylpropanoate (**6e**)

File :C:\MSDCHEM\1\DATA.7\Snapshot\GREENSOC_620.d
Operator : ORIANA
Acquired : 26 Mar 2025 15:19 using AcqMethod METHODA.M
Instrument : greensoc
Sample Name: Ni-182 RID FENILACRILATO
Misc Info :
Vial Number: 1

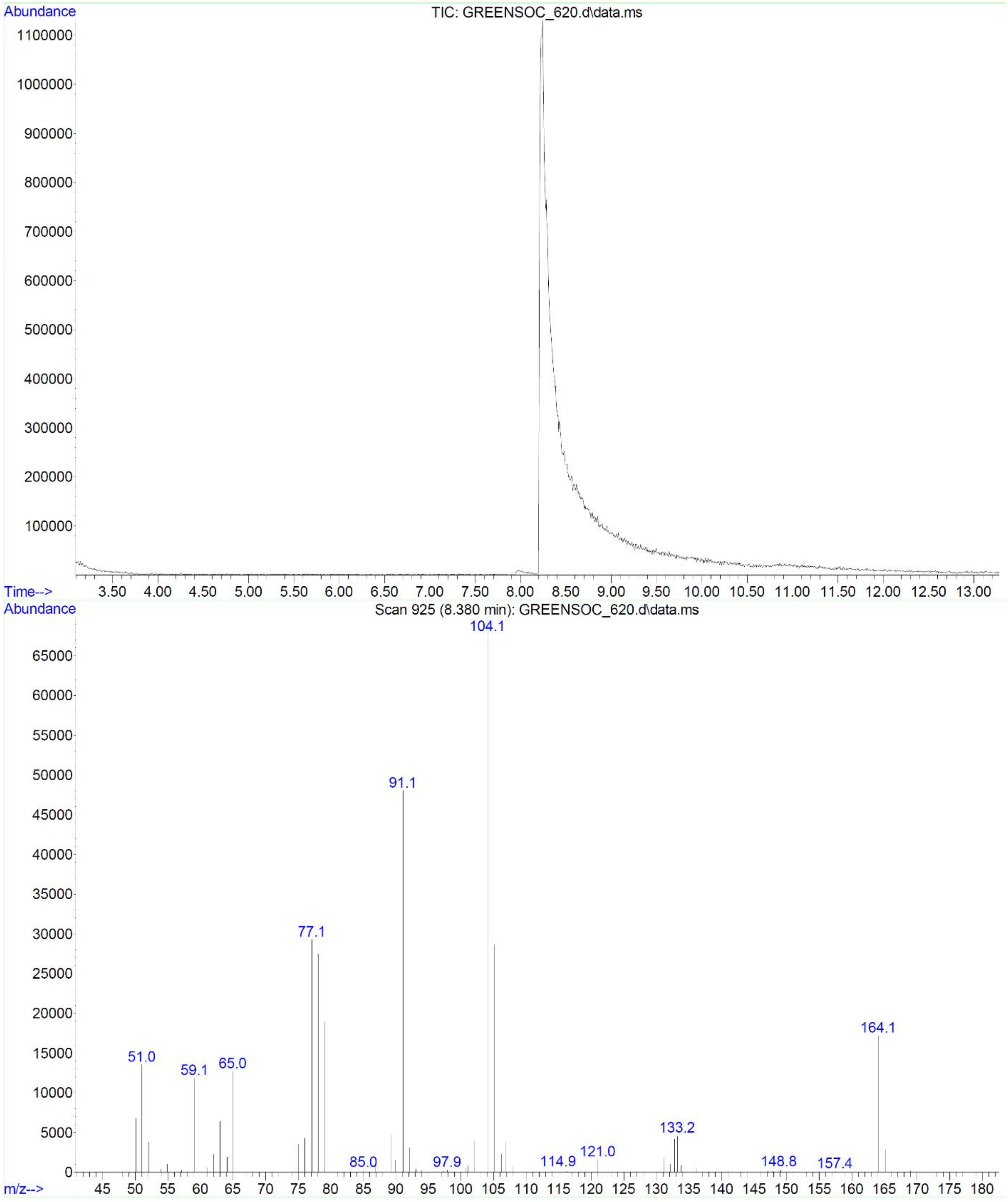

Figure S33: Hydrogenation of 1-phenylcyclohexene (**5f**): MS spectrum of cyclohexylbenzene (**6f**)

File :C:\MSDCHEM\1\DATA.7\Snapshot\GREENSOC_618.d
Operator : ORIANA
Acquired : 26 Mar 2025 14:07 using AcqMethod METHODA.M
Instrument : greensoc
Sample Name: Ni- RID FENILCICLOESENE
Misc Info :
Vial Number: 1

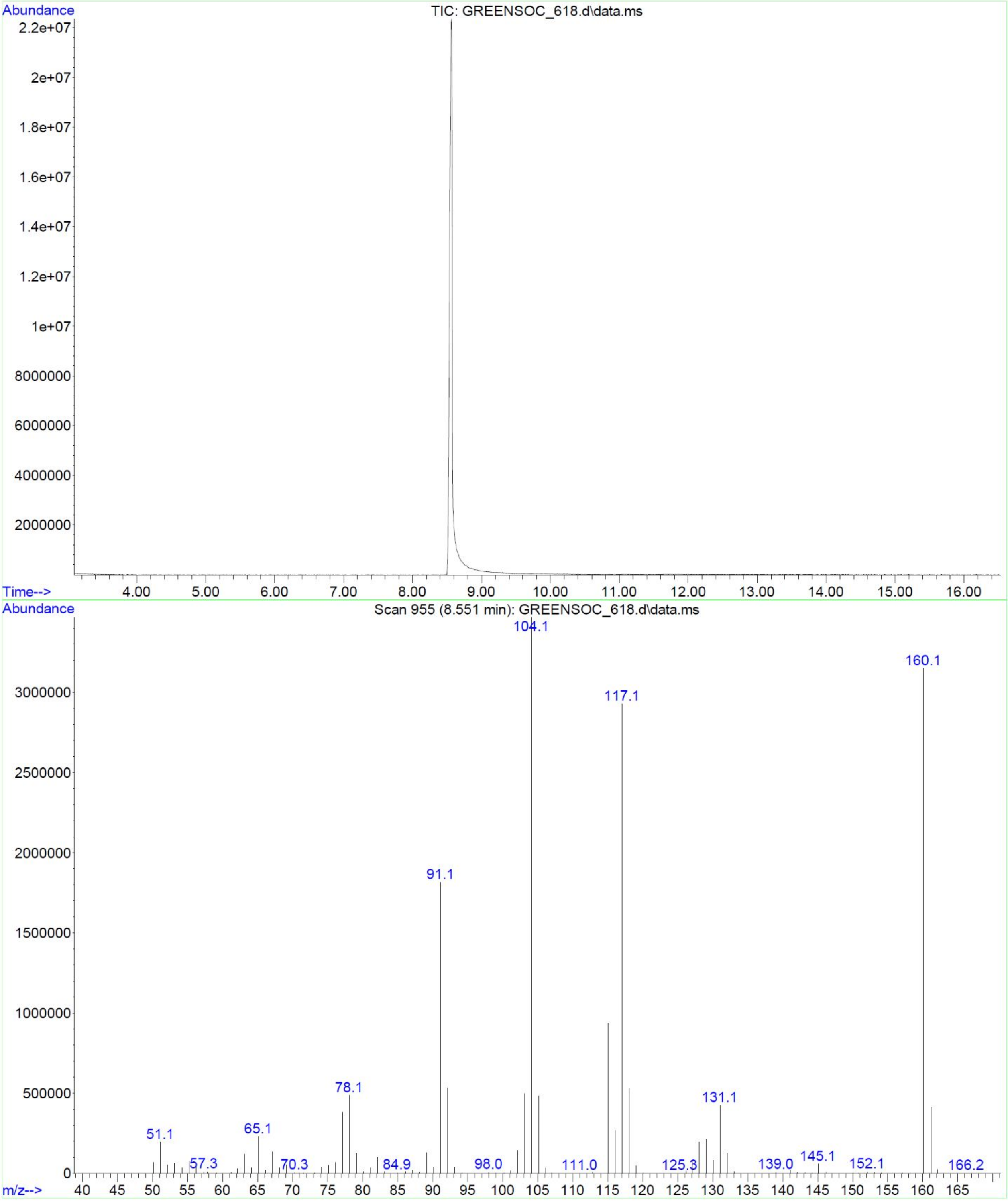

Figure S34: Hydrogenation of cyclooctene (**5g**): MS spectrum of cyclooctane (**6f**)

File :C:\MSDCHEM\1\DATA.7\Snapshot\GREENSOC_106.d
Operator : ORIANA
Acquired : 28 Mar 2024 17:30 using AcqMethod METHODA.M
Instrument : greensoc
Sample Name: NI 184
Misc Info :
Vial Number: 1

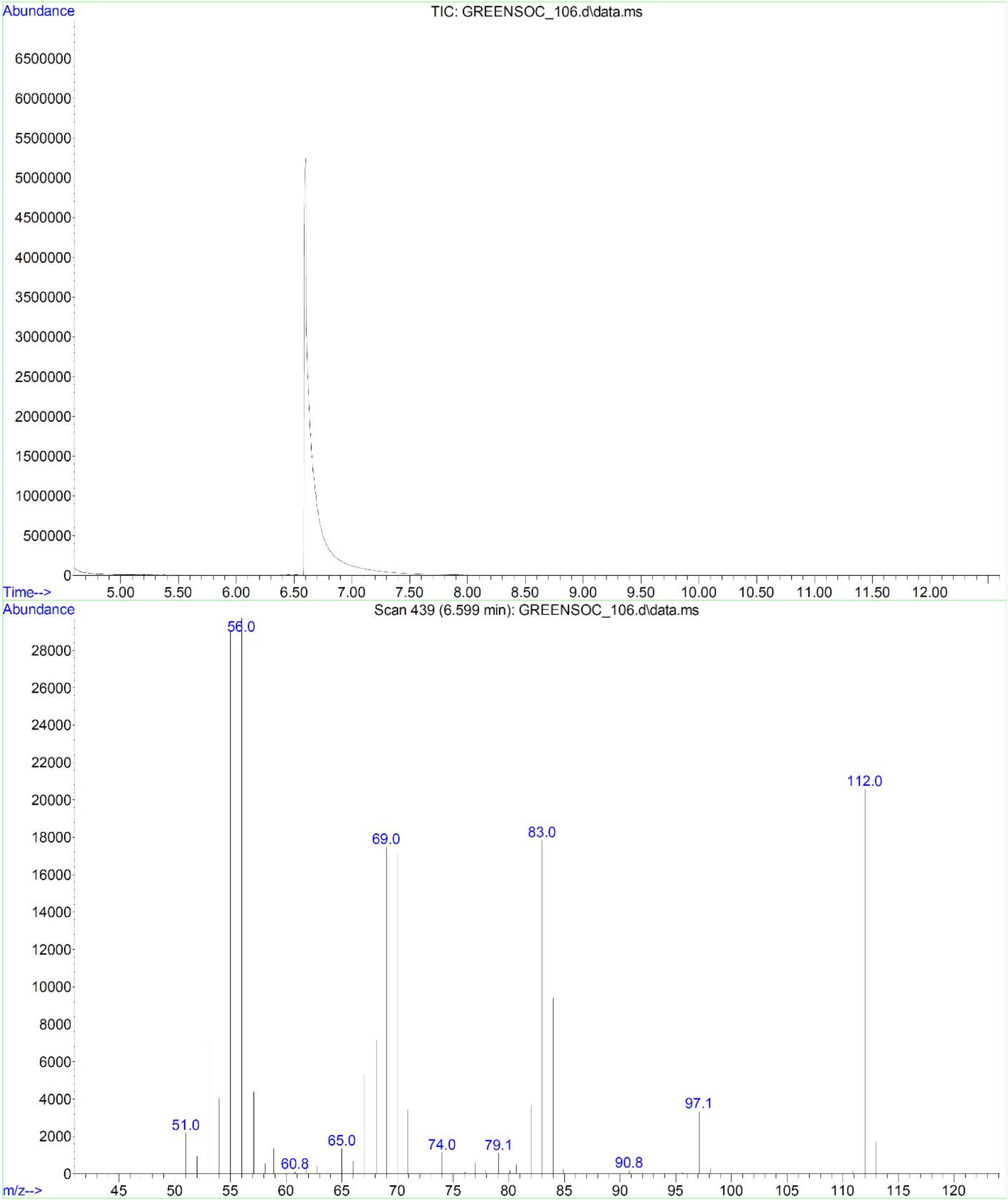

[1] S. Fountoulaki, V. Daikopoulou, P. L. Gkizis, I. Tamiolakis, G. S. Armatas, I. N. Lykakis, *ACS Catal.* **2014**, *4*, 3504-3511

[2] X.-B. Lou, L. He, Y. Qian, Y.-M. Liu, Y. Cao, K.-N. Fan, *Adv. Synth. Catal*. **2011**, *353*, 281-286.

[3] W.-G. Jia, H. Zhang, T. Zhang, D. Xie, S. Ling, En-H. Sheng, *Organometallics* **2016**, *35*, 503-512.

[4] X. Liu, S. Ye, H.-Q. Li, Y.-M. Liu, Y. Cao, K.-N. Fan, *Catal. Sci. Technol.* **2013**, *3*, 3200-3206.

[5] P. S. Rathore, R. Patidar, T. Shripathic, S. Thakore, *Catal. Sci. Technol.* **2015**, *5*, 286-295.

[6] Q. Shuai, J. Li, F. Zhao, W. Su, G. Deng, *Chem. Paper* ***2019**, 73*, 965–975.

[7] I. Sorribes, L. Liu, A. Corma, *ACS Catal.* **2017**, *7*, 2698–2708

[8] Z. S. Qureshi, P. B. Sarawade, M. Albert, V. D'Elia, M.N. Hedhili, K. Kçhler, J.-M. Basset, *ChemCatChem* **2015**, 7, 635 – 642.

[9] M. Espinal-Viguri, S. E. Neale, N. T. Coles, S. A. Macgregor, R. L. Webster, J. Am. Chem. Soc. **2019**, *141*, 572–582

[10] C.-Z. Zhang, H. Yang, D.-L. Wu, G.-Y. Lu, *Cinese J. Chem.* **2007**, *25*, 653-660.

[11] M. Gholinejad, E. Oftadeh, M. Shojafar, J. M. Sansano, B. H. Lipshutz, *ChemSusChem* **2019**, **12**, 4240-4248

[13] Mass spectra were identified by comparison with the **NIST/EPA/NIH Mass Spectral Library (NIST 20)**, National Institute of Standards and Technology.

[14] Y. Liu, S. Xiao, Y Qi, F. Du, *Asian J. Org. Chem.* **2017**, *12*, 673-678.
